# Plasma proteomics improves risk prediction in heart failure and reveals unique biology in chronic chagas cardiomyopathy

**DOI:** 10.1371/journal.pntd.0014370

**Published:** 2026-06-08

**Authors:** José S. L. Patané, Fernando R. Giugni, Rogério S. Rosa, Fabiana G. Marcondes-Braga, Alfredo J. Mansur, Alexandre C. Pereira, Jose E. Krieger

**Affiliations:** 1 Instituto do Coracao (InCor), Hospital das Clinicas HCFMUSP, Faculdade de Medicina, Universidade de Sao Paulo, Sao Paulo, Brazil; 2 Brigham and Women’s Hospital, Harvard Medical School, Boston, Massachussets, United States of America; INGEBI, ARGENTINA

## Abstract

**Background:**

Chronic Chagas cardiomyopathy (CCC) remains a major cause of heart failure (HF)–related mortality in Latin America and is increasingly recognized as a global health concern. Prognostic models developed in non-Chagas populations often perform poorly in CCC, highlighting the need for etiology-specific risk stratification.

**Methodology/principal findings:**

We applied high-throughput plasma proteomics to evaluate 2-year mortality risk in CCC compared with other HF etiologies. Baseline plasma from 1,212 adults with heart failure with reduced ejection fraction (HFrEF; LVEF <50%) was analyzed to quantify 734 circulating proteins. CCC was confirmed in 191 participants (16%) by dual *Trypanosoma cruzi* serology. Two-year mortality was higher in CCC than in the overall HF cohort (26% vs. 16%, *p* < 0.01). Feature-selection methods identified a nine-protein panel (P9: C1QA, CCL4, REN, EGLN1, COL9A1, GP1BA, ITM2A, CNPY2, NT-proBNP) that improved risk classification compared with NT-proBNP alone, increasing F1-macro by 20% (0.674 vs. 0.560) and integrated time-dependent discrimination for 2-year mortality (iAUC) by 6%. Performance gains varied by HF etiology. Improvements were greatest in hypertensive (+40%) and ischemic (+21%) HF, whereas in CCC the P9 panel underperformed NT-proBNP alone (−16%), suggesting distinct underlying disease biology. External validation in the UK Biobank confirmed generalizability: compared with NT-proBNP, P9 improved F1-macro by 18% and iAUC by 7.4%, reaching an F1-macro of 0.612 in the highest-risk tertile. Pathway enrichment identified 14 CCC-specific pathways, mainly related to fibrosis, integrin signaling, immune dysregulation, and impaired protein trafficking. Exploratory analyses also highlighted potential pathway-linked therapeutic targets consistent with distinct CCC mechanisms.

**Conclusions/significance:**

The P9 proteomic panel improved mortality risk prediction beyond NT-proBNP and the MAGGIC clinical score across most HF etiologies and showed consistent performance in an independent population-based cohort. In contrast, in CCC P9 underperformed NT-proBNP alone, highlighting the distinct biological features of this disease. These findings underscore the limitations of universal biomarker models in CCC and support the need for etiology-specific risk stratification strategies.

## Author summary

Chagas disease remains a major cause of heart failure in Latin America and is increasingly recognized in other parts of the world. Heart failure occurs when the heart can no longer pump blood effectively, and doctors often rely on clinical scores and blood tests to estimate a patient’s risk of complications or death. However, most of these tools were developed in patients with other types of heart failure and may not fully reflect the biological processes involved in Chagas cardiomyopathy. To address this question, we measured 734 proteins circulating in the blood of 1,212 patients with heart failure, including 191 with Chagas disease. We identified a group of nine proteins that improved prediction of death over two years in heart failure overall and confirmed this finding in an independent population cohort. When we examined patients with Chagas cardiomyopathy more closely, however, we observed a distinct set of protein signals linked to processes such as tissue scarring, immune system activation, and abnormalities in cellular protein handling. These results suggest that Chagas cardiomyopathy follows different biological pathways from other forms of heart failure and may require disease-specific biomarkers and treatment strategies. Understanding these differences may help guide more precise approaches to diagnosis and care for this neglected disease.

## Introduction

Heart failure (HF) is a prevalent, heterogeneous, and debilitating syndrome affecting over 55 million people globally, with an annual mortality approaching 20% despite therapeutic advances [1,2]. Prognosis varies widely and is shaped by demographic, clinical, and physiological factors, including advanced age, male sex, Black ethnicity, ischemic etiology, reduced body mass index, lower systolic blood pressure, and impaired left ventricular systolic function (LVEF) [3–7]. These variables form the basis of established risk scores such as the Heart Failure Survival Score [3], Seattle Heart Failure Model [5], CHARM risk score [7], and MAGGIC score [6], which are designed to inform prognosis and clinical decision-making.

The integration of circulating biomarkers, especially natriuretic peptides like NT-proBNP, has enhanced the predictive accuracy of these models [8,9]. Current HF guidelines recommend their use for diagnosis, prognostication, and therapeutic monitoring [10]. Nevertheless, risk scores often perform suboptimally in real-world settings and are infrequently incorporated into routine care, in part due to limited discrimination, generalizability, and mechanistic insight [11]. Notably, most existing tools were developed in predominantly White populations with ischemic or idiopathic HF, limiting their utility in diverse populations and non-ischemic etiologies [12].

Advances in high-throughput plasma proteomics now allow the simultaneous quantification of hundreds to thousands of proteins from small blood volumes, enabling the discovery of novel biomarkers linked to disease biology and outcomes [13–15]. Proteomic signatures offer potential for improved phenotyping, risk stratification, and identification of therapeutic targets in HF. Prior studies have demonstrated associations between specific protein panels and mortality in HF cohorts [16,17]. However, these studies have largely focused on European ancestry populations and provided limited insight into etiology-specific pathophysiology, particularly in neglected diseases such as Chagas cardiomyopathy.

Chronic Chagas cardiomyopathy (CCC), caused by *Trypanosoma cruzi* infection, is a leading cause of non-ischemic dilated cardiomyopathy in Latin America and an emerging global health concern due to migration [18]. CCC is associated with disproportionately high mortality compared to other HF etiologies, driven by distinct mechanisms including immune-mediated myocardial injury, diffuse fibrosis, arrhythmias, and thromboembolic events [19]. Despite its clinical severity, CCC remains underrepresented in HF research, and existing risk scores fail to capture its unique risk profile. There is an urgent need for robust, CCC-specific prognostic tools to improve patient stratification and guide precision management strategies.

To address this critical gap, we leveraged deep plasma proteomic profiling in a large and etiologically diverse Brazilian heart failure with reduced ejection fraction (HFrEF) cohort (GENIUS-HF) [20,21]. Our aims were threefold: (I) to develop sparse, multi-protein models optimized for predicting all-cause mortality in HFrEF including CCC; (II) to benchmark their performance against standard biomarkers such as NT-proBNP across HF etiologies, particularly CCC; and (III) to identify CCC-specific molecular pathways, providing insight into disease mechanisms and potential therapeutic targets. We hypothesized that a multi-protein model would have better prognostic performance than existing tools across HFrEF etiologies and that CCC would present unique biological pathways.

## Methods

### Ethics statement

The study protocol was approved by the Institutional Review Board at Hospital das Clínicas da Faculdade de Medicina da Universidade de Sao Paulo (HC-FMUSP) (CAAE - 70162117.0.0000.0068), and all participants provided written informed consent.

### Study design and cohort

This single-center prospective cohort study included HF patients with LVEF below 50% referred to the cardiology clinic at the Heart Institute (InCor) of the Hospital das Clínicas, University of São Paulo Medical School (HC-FMUSP), Brazil, between August 2012 and January 2015 [20,21]. Blood samples were collected, and echocardiograms were performed by board-certified specialists as part of the first patients’ visit to the cardiology clinic. Patients with LVEF < 50% assessed were enrolled.

Baseline clinical characteristics of the participants were recorded. Sex and ethnicity were self-reported, with ethnic categories following the Brazilian Institute of Geography and Statistics (IBGE) 2022 Census definitions: White, Brown, Black, Yellow, or Indigenous. Hypertension, diabetes, smoking status, occupation, and marital status were self-reported. Low socioeconomic status was defined as having completed up to primary school. Blood pressure, heart rate, height, and weight were measured using standardized protocols. Medication use was gathered from prescription records at clinic visits. Glomerular filtration rate was estimated using the CKD-EPI formula. Socioeconomic variables were self-reported. Low educational level was defined as illiteracy or up to elementary school education. Equivalized income was calculated as the monthly family income divided by the square root of the number of dependents, and converted to US dollars using the exchange rate (1 Brazilian Real = 0.20 USD).

HF etiology was determined based on clinical records and confirmed by a board-certified cardiologist. Patients were followed for two years and deaths were ascertained continuously through hospital electronic records and the National Death Records repository, as all deaths in Brazil are legally required to be reported. Chagas disease etiology was confirmed by serological evidence of IgG antibodies to *Trypanosoma cruzi* by immunoenzymatic and indirect immunofluorescence assays. Ischemic HF etiology was defined as a history of myocardial infarction, coronary artery bypass graft surgery, or obstructive coronary artery disease. Medication dosage was recorded from the most recent prescriptions at enrollment.

### Descriptive analyses

Statistical differences were evaluated using one-way ANOVA for continuous variables and the chi-square test for categorical variables. Post-hoc analyses involved Tukey’s HSD for continuous variables, or standardized residuals for categorical variables, at a nominal α = 0.05.

### Pre-processing

Samples with less than 99% protein data or missing covariates were excluded. Missing values were imputed using column medians. Median imputation was selected due to the presence of skewed distributions and extreme values in our clinical features. Unlike mean or linear regression-based imputation (e.g., common in MICE workflow), the median is resistant to outliers, ensuring that imputed values remain within a plausible clinical range and do not artificially inflate the variance of predictors. Furthermore, the number of missing values across the dataset prior to imputation was ~ 2% and of a sparse nature (i.e., without clear signs of clustering of columns within same rows bearing missing data). Biomarker levels were *z*-scored for proper comparisons.

### Proteomic panel quantification

Proteomic analysis was conducted using the Olink Explore 384 Cardiometabolic and Inflammation panels, to assess 734 different proteins in baseline plasma samples. Peripheral venous blood was collected into EDTA tubes, centrifuged to obtain plasma, aliquoted to avoid repeated freeze–thaw cycles, stored at -80 °C, and shipped on dry ice for biomarker quantification. Olink methodology has been previously described [22]. Briefly, it uses Proximity Extension Assay, where a matched pair of antibodies labelled with unique complimentary oligonucleotides bind to the respective target protein in a sample, leading to probe hybridization and enabling DNA amplification of the protein signal through next generation sequencing (NGS). Counts of known barcode sequences were thereafter translated into normalized protein expression (NPX) units, a relative protein quantification unit with values on a log2 scale. Data generation of NPX consists of three main steps: normalization to the extension control (known standard), log2-transformation, and level adjustment using the plate control (plasma sample). Quality-control steps include: average matched counts for each sample (≥500 counts to pass QC); deviation from the median value of the incubation- and amplification controls for each individual sample (≤ + /-0.3 NPX); and deviation of the median value of the negative controls from a predefined value set for each assay (≤5 SD). All samples were collected and processed at a single center and shipped together for analysis in a single Olink project run; therefore, no additional post-hoc batch effect correction beyond Olink’s standard NPX normalization was applied. NT-proBNP measurements reported in the analyses were based on Olink measurements, as part of the Cardiometabolic panel. Further details on the Olink technology are available at https://www.olink.com/. Information on all quantified proteins is provided in S1 Table.

### Testing for batch effects

All analyses were performed using python [23] and R [24] scripts. Principal component analysis (PCA) was applied to the protein markers, and the resulting components were visualized with markers colored according to their Olink panel. This analysis was used to assess whether proteins cluster by panel, which would indicate potential panel-related batch effects.

### Survival curve estimation

Kaplan-Meier survival curves were generated for the whole cohort and by HF etiology using the *R* libraries *survival* v3.8.3 [25] and *survminer* v0.4.9 [26]. Hazard ratios for each etiology were calculated using Cox proportional hazards models (*coxph* function). Proportional hazard assumptions were validated using Schoenfeld residuals.

### Train and test sets

The dataset was split into training (70%; 848 patients) and testing (30%; 364 patients) subsets using the *R caret* v6.0.94 package [27], ensuring balanced proportions of the variables age, sex, NT-proBNP, LVEF, and body mass index (BMI). All analyses and model optimization procedures were performed using the training dataset, primarily through Monte Carlo approaches (1,000 bootstrap resamples or permutations, as specified for each analysis). Model parameters and performance metrics were evaluated on a validation set obtained from a further 70% train/30% validation split of the training data, and summary statistics (e.g., median F1-macro) were subsequently computed across iterations in the validation set.

Within this train/validation framework, we conducted analyses of biomarker associations, feature selection, 2-year outcome prediction, prediction across longitudinal follow-up time points, and pathway enrichment. To assess the generalizability of the findings, the models demonstrating the best performance in the train/validation phase were subsequently applied to the held-out test dataset (the 364 patients previously unseen by the fitted models). Finally, external generalizability was evaluated by applying the selected models to an independent cohort, the UK Biobank (UKBB). Detailed descriptions of each analysis are provided in the corresponding sections below.

### Biomarker informativeness

To identify individual biomarkers associated with HF etiology, logistic regression was applied, adjusting for age, sex, and LVEF. Proteins with FDR-corrected *p* ≤ 0.05 were considered significant.

### Classification model selection

A total of ten machine learning models were tested using scikit-learn v2.2.3 [28] for the development of multi-marker subsets (logistic regression, ridge classifier, SVM, random forest, XGBoost, naive Bayes, K-nearest neighbors, linear discrimination analysis, adaboost, and gradient boosting), for both mortality and mortality + heart transplantation (composite) outcomes. The metric assessed for performance was F1-macro (average between F1 obtained for alive and for the deceased) to optimize its performance at predicting both classes; furthermore, F1-macro is less affected by imbalanced classes (as deaths inflicted ~16% of the patients). Variability was assessed by 1,000 random permutations of the train set, generating a 70% train/30% validation set at each replicate, where model was fitted on train set and the F1-macro scored on the validation set.

### Events per variable

The EPV (Events Per Variable), here estimated as the number of deaths in the training set (where all analyses and models were estimated) divided by the number of predictor variables (i.e., independent variables and covariates), was checked for NT-proBNP and P9 models, to check for statistical power of the model.

### Feature selection

We aimed at identifying a smaller, yet equally effective or potentially superior, subset of markers for outcome prediction, thereby aiming at both performance and model simplicity. First, a genetic algorithm (GA) was employed, using library *geneticalgorithm* v1.0.2 [29]. It identified an optimal subset from the 734 proteins while allowing inclusion of age, gender, and ejection fraction as clinical covariates. F1-macro score of the best machine learning classifier was maximized over 1,000 generations, utilizing uniform crossover and a 0.15 mutation probability to navigate the combinatorial search space. The aim of this first step was to remove noisy features, but without limiting the final number of proteins in the model.

Second, Bayesian stochastic search variable selection (SSVS) was implemented with library *millipede* v0.2.0 [30], to retrieve posterior inclusion probabilities (PIP) of the features selected in the previous step. Marker sets including either the top ranked protein alone, or the two best, and so on with successively inclusive models, were assessed by F1-macro. The goal was to obtain a minimal set of proteins that could generate a final F1-macro score higher or at least equal to the model with all pre-selected features from GA.

### Assessment of overfitting

Optimism correction of P9 model was performed using 1,000 bootstrap resampling, with each bootstrap used to train the model and then evaluated on both the bootstrapped and original train sets, separately. The difference in F1-macro scores provided an optimism estimate, averaged across iterations and subtracted from the apparent performance to obtain the corrected F1-macro. Validation was carried out on the test set.

### Prediction across follow-up time points

Cox proportional-hazards (CPH) models were employed for time-to-event analysis, using the optimal protein marker set identified through the feature selection procedure. Covariates included sex, age, and LVEF, along with the relative date of death for deceased patients. Model fitting was performed using the function *CoxPHSurvivalAnalysis* in *sksurv* v0.23.1 [31] python library. Individuals who survived beyond the follow-up period were assigned a value of 730 days (two years) in the “time” variable (time-to-event) and a value of 0 in the “death” variable (outcome). Although the study design involved administrative right-censoring at two years, no individuals were lost to follow-up. This reflects the characteristics of the cohort, which comprised patients with relatively severe cardiac dysfunction (LVEF < 50%); consequently, participants were likely to maintain clinical contact with the healthcare system, either through hospital visits or follow-up reporting, given the need for ongoing management and treatment of their condition.

We employed a cumulative/dynamic (C/D) framework to compute the time-dependent ROC curves underlying the iAUC (integrated area under the curve across time). In this scenario, at each time point *t*, individuals who have experienced the event up to time *t* are considered cases (cumulative cases), while individuals who remain event-free beyond *t* are considered controls (dynamic controls). The time-dependent AUC therefore measures the probability that the model assigns a higher predicted risk to an individual who experiences the event before time *t* than to one who survives beyond *t*. By repeating this comparison across multiple time points during follow-up and integrating the resulting AUC values, the iAUC is obtained. The latter provides a measure of discrimination across the two-year follow-up, with higher values indicating better risk ranking [32].

Models were fitted on train set, then applied on the test set for prediction. Extrapolation to the test was done by applying the partial hazard estimates from the train set. The 95% interval and p-value were estimated via non-parametric bootstrapping (1,000 resamples). For each replicate, we calculated the difference in integrated AUC (ΔiAUC = P9 - BNP). The 95% highest density interval (HDI) and the p-value, defined as the empirical probability P (P9 ≤ BNP), a measure of superiority of P9 compared to NT-proBNP in case significant at p = 0.05, were then derived from the bootstrap distribution.

The analysis was restricted to time points with events occurring ≥ 50 days after baseline, ensuring sufficient numbers of cases and controls for reliable estimation of the time-dependent discrimination.

### Effect size estimation

Cohen’s d_AV_ [33] was chosen to quantify performance differences between models as it is well-suited for paired comparisons where results are correlated across the same resampling splits. It standardizes the mean difference using the average of the models’ standard deviations, providing a balanced measure of effect size.

### Calibration and decision curve analysis

We employed *CalibratedClassifierCV* function from scikit-learn for calibration analysis. The training dataset was partitioned into a 5-fold cross-validation (CV) scheme. During the CV process, the model was iteratively trained on four folds and used to generate predicted values (scores) for the remaining held-out fold. Upon completion of all folds, these out-of-sample predictions were aggregated into a single set of calibrated values for all training samples. Subsequently, thresholding was applied to this combined, calibrated model to optimize the decision boundary for classification. Finally, the calibrated model with the optimized classification threshold was applied to the unseen test set. The predictive performance was evaluated by plotting test-set predicted values against their ground truth values, from which the final error metrics and the percentage of error reduction were derived (using Brier scores).

We further implemented Decision Curve Analysis (DCA), using the *dcurves* v1.1.7 library [34] to evaluate the clinical utility of two predictive models (BNP and P9) for 2-year mortality. Predicted probabilities of death were generated for the test cohort and used to compute net benefit across threshold probabilities ranging from 0 to 50%. Net benefit was calculated as the proportion of true positives adjusted for false positives weighted by the odds of the threshold probability. DCA curves were plotted for each model alongside “treat all patients” and “treat none” strategies.

### Including other covariates

Equivalized income and NYHA (New York Heart Association) categories were separately included as covariates in the models, to test for further model improvement.

### External validation

We externally validated the P9 model using data from the UK Biobank under Application Number [519993], a population-based cohort of approximately 500,000 individuals aged 40–69 years recruited between 2006 and 2010 across England, Wales, and Scotland [35]. Among 54,219 participants with baseline plasma proteomic profiling (Olink Explore 3072), 431 presented HF, defined by ICD-10 code I50 in medical records prior to enrollment, and available proteomic measurements. Participants were followed through linkage to electronic health records. Because the UKBB is a community-based cohort with generally milder HF than the GENIUS-HF cohort, analyses were stratified by tertiles of the MAGGIC risk score, with sub analyses focusing on individuals in the highest tertile of disease severity. To account for right-censoring, time-dependent area under the curve (AUC_t_) was estimated using the inverse probability of censoring weighting (IPCW) method of Uno et al [32]. Additional evaluation metrics included F1-macro score, integrated AUC (iAUC), sensitivity, specificity, positive predictive value (PPV), and net reclassification improvement (NRI) for events and non-events.

### Protein and pathway enrichment analyses

To investigate differential enrichment in specific etiologies among HFrEF patients, we performed a multi-layered enrichment analysis. From a total of 733 protein markers analyzed (disregarding NT-proBNP, as it is included as a covariate for protein enrichment), we sought those exclusively associated with each etiology, as a means of investigating the biological implications of dysregulated proteins.

Differential pathway enrichment by GSEA (Gene Set Enrichment Analysis), using library *gseapy* v1.1.9 [36], was employed for each set of etiology-associated proteins, using their corresponding regression coefficients (betas) as the pre-ranking metric. Nine databases included in the library were screened in the train set (KEGG_2021_Human, GO_Biological_Process_2025, Reactome_Pathways_2024, WikiPathways_2024_Human, MSigDB_Hallmark_2020, BioPlanet_2019, GO_Cellular_Component_2025, GO_Molecular_Function_2025, and Human_Phenotype_Ontology), including 1,806 pathways. A total of 1,000 permutations were performed per etiology. Genes present in a pathway being analyzed, but absent from our dataset, were removed from the former prior to the pathway’s p-value estimation. Only pathways with FDR ≤ 0.05 were kept for subsequent screening on the test set. Significant pathways occurring in both train and test data were deemed biologically relevant and further discussed.

### Screening potential therapeutic targets

To further prioritize key molecular players, we built a network of genes that appeared in two or more of these enriched pathways in the test set (for each etiology). We screened this resulting network for druggable targets by identifying gene hubs (nodes with ≥3 connections) and cross-referencing them against five curated pharmacological databases: DGIdb (Drug-Gene Interaction Database; [37], ChEMBL [38], Pharos [39], DrugBank [40], and Therapeutic Target Database [41]. Only approved drugs were kept for biological discussion.

## Results

### Baseline characteristics

We enrolled 1,212 HFrEF patients, classified by etiology as idiopathic (27%), hypertensive (24%), ischemic (20%), CCC (16%), alcoholic (8%), and “other” (6%). Table 1 details mortality rates, demographics, clinical status, therapies, and socioeconomic factors. Overall mortality was 16%, highest in CCC (26%) and ischemic (19%) patients. CCC patients showed other distinct features, including lower mean BMI (25.8 kg/m²), systolic/diastolic blood pressures, fewer comorbidities (hypertension, diabetes) but more advanced HF symptoms (22% NYHA III/IV) and higher mortality rate, compared to overall HF. Participants with CCC also displayed greater socioeconomic vulnerability (83% low education; mean income USD 165 ± 122; 20% employed). Guideline-directed medical therapy uses at the time of the study exceeded 90% across all groups (Table 1).

**Table 1:** Baseline Characteristics of HFrEF patients.

	TOTAL	Idiopathic	Hypertensive	Ischemic	Chagas disease	Alcoholic	Other
	N = 1212	N = 322	N = 292	N = 243	N = 191	N = 92	N = 72
**Mortality**							
Mortality rate, n(%)	189 (0.16)	45 (0.14)	36 (0.12)	45 (0.19)	50 (0.26)*	9 (0.10)	4 (0.06)
Mortality LVEF ≥ 30, n(%)	89 (0.07)	18 (0.06)	24 (0.08)	27 (0.11)*	15 (0.08)	3 (0.03)	2 (0.03)
Mortality LVEF < 30, n(%)	100 (0.08)	27 (0.08)	12 (0.04)*	18 (0.07)	35 (0.18)*	6 (0.07)	2 (0.03)
**Demographics**							
Age, y	56 ± 12	54 ± 13*	57 ± 11	61 ± 10*	57 ± 11	52 ± 11*	50 ± 15
Women, n(%)	424 (35%)	124 (39%)	113 (39%)	58 (24%)*	85 (45%)*	2 (2%)*	42 (58%)
Caucasian, n(%)	443 (36%)	131 (41%)*	87 (30%)*	113 (46%)*	52 (27%)*	27 (29%)	33 (45%)
Afro-American, n(%)	180 (15%)	44 (14%)	59 (20%)*	23 (9%)*	25 (13%)	24 (26%)*	5 (7%)
Admixed, n(%)	577 (47%)	141 (44%)	143 (49%)	106 (43%)	113 (59%)*	41 (45%)	33 (45%)
**Marital status**							
Married, n(%)	697 (57%)	166 (52%)*	174 (60%)	151 (62%)	110 (58%)	52 (57%)	44 (60%)
Single, n(%)	226 (19%)	74 (23%)*	49 (17%)	35 (14%)*	34 (18%)	21 (23%)	13 (18%)
Divorced, n(%)	132 (11%)	37 (12%)	22 (8%)	30 (12%)	22 (12%)	15 (16%)*	6 (8%)
Widower, n(%)	119 (10%)	33 (10%)	34 (12%)	26 (11%)	19 (10%)	1 (1%)*	6 (8%)
**Clinical characteristics**							
BMI, kg/m2	27.8 ± 5.9	27.4 ± 5.5	29.9 ± 7.2*	27.5 ± 5.3	25.8 ± 4.6*	26.8 ± 4.9	28.5 ± 6.1
Hypertension, n(%)	778 (64%)	154 (48%)*	292 (100%)*	178 (73%)*	81 (42%)*	51 (55%)	39 (53%)
Diabetes mellitus, n(%)	355 (29%)	69 (22%)*	107 (37%)*	109 (45%)*	41 (21%)*	17 (18%)*	12 (17%)
Current smoker, n(%)	122 (10%)	30 (9%)	29 (10%)	27 (11%)	14 (7%)	18 (20%)*	4 (5%)
Ejection fraction, %	33 ± 9	31 ± 8	34 ± 9	33 ± 8	32 ± 9	30 ± 9*	36 ± 9
Functional class II (NYHA)	730 (61%)	201 (63%)	167 (58%)	143 (60%)	116 (61%)	55 (60%)	48 (67%)
Functional class III/IV (NYHA)	228 (19%)	54 (17%)	56 (19%)	50 (21%)	42 (22%)	13 (14%)*	13 (18%)
Systolic BP, mmHg	124 ± 24	119 ± 21*	136 ± 25*	125 ± 24	114 ± 20*	126 ± 22	118 ± 21
Diastolic BP, mmHg	76 ± 15	74 ± 14*	82 ± 16*	76 ± 16	71 ± 13*	80 ± 14	73 ± 13
Heart rate	71 ± 14	72 ± 14	73 ± 15	70 ± 13	66 ± 12*	74 ± 16	72 ± 13
Serum Creatinine, mg/dL	1.24 ± 0.73	1.19 ± 0.76	1.38 ± 1.04*	1.32 ± 0.62*	1.15 ± 0.33	1.11 ± 0.25	1.04 ± 0.35
eGFR	68.5 ± 23	72.4 ± 23*	63.7 ± 23*	62.4 ± 21*	67.3 ± 20	79.7 ± 17*	79.5 ± 34
**Medical treatment at baseline**							
ACEi/ARB, n(%)	1095 (90%)	291 (91%)	262 (90%)	205 (84%)*	179 (94%)	90 (98%)*	68 (93%)
Beta blocker, n(%)	1170 (97%)	310 (97%)	281 (97%)	239 (98%)	180 (95%)	90 (98%)	70 (96%)
MR antagonist, n(%)	818 (67%)	218 (68%)	188 (64%)	149 (61%)	135 (71%)	74 (80%)*	54 (74%)
Digitalis, n(%)	249 (21%)	89 (28%)*	55 (19%)	27 (11%)*	31 (16%)	27 (29%)	20 (27%)
GDMT, n(%)*	1213 (100%)	321 (100%)	292 (100%)	245 (100%)	190 (99%)	92 (100%)	73 (100%)
**Socioeconomic/occupation status**							
Low education, n(%)	820 (68%)	181 (57%)*	206 (71%)	166 (68%)	159 (83%)*	78 (85%)*	30 (41%)
Employed, n(%)	325 (27%)	95 (30%)	95 (33%)*	50 (21%)*	37 (20%)*	33 (36%)*	15 (21%)
Unemployed, n(%)	261 (22%)	72 (22%)	57 (20%)	52 (21%)	39 (21%)	21 (23%)	20 (27%)
Retired/Away, n(%)	621 (51%)	153 (48%)	138 (48%)	141 (58%)*	113 (60%)*	38 (41%)*	38 (52%)
Equivalised income (U$) ‡	192 ± 149	207 ± 183	186 ± 130	205 ± 134	165 ± 122*	163 ± 143*	204 ± 164

Baseline characteristics of HFrEF patients, describing demographic, clinical, therapeutic, and socioeconomic variables for 1,212 patients stratified by HF etiology (idiopathic, hypertensive, ischemic, Chagas cardiomyopathy, alcoholic, and other conditions). Data are presented as mean ± SD for continuous variables and n (%) for categorical count data; the “Other” category (n = 72) was excluded from all comparative statistical calculations. Statistical differences between the five main etiologies were assessed using One-Way ANOVA for continuous parameters and the Chi-Square test for categorical counts. Asterisks (*) denote specific classes identified via post-hoc analysis (Tukey’s HSD or standardized residuals) as significantly deviating from the respective expected distribution (p = 0.05).

### Survival analysis

**Fig 1:**
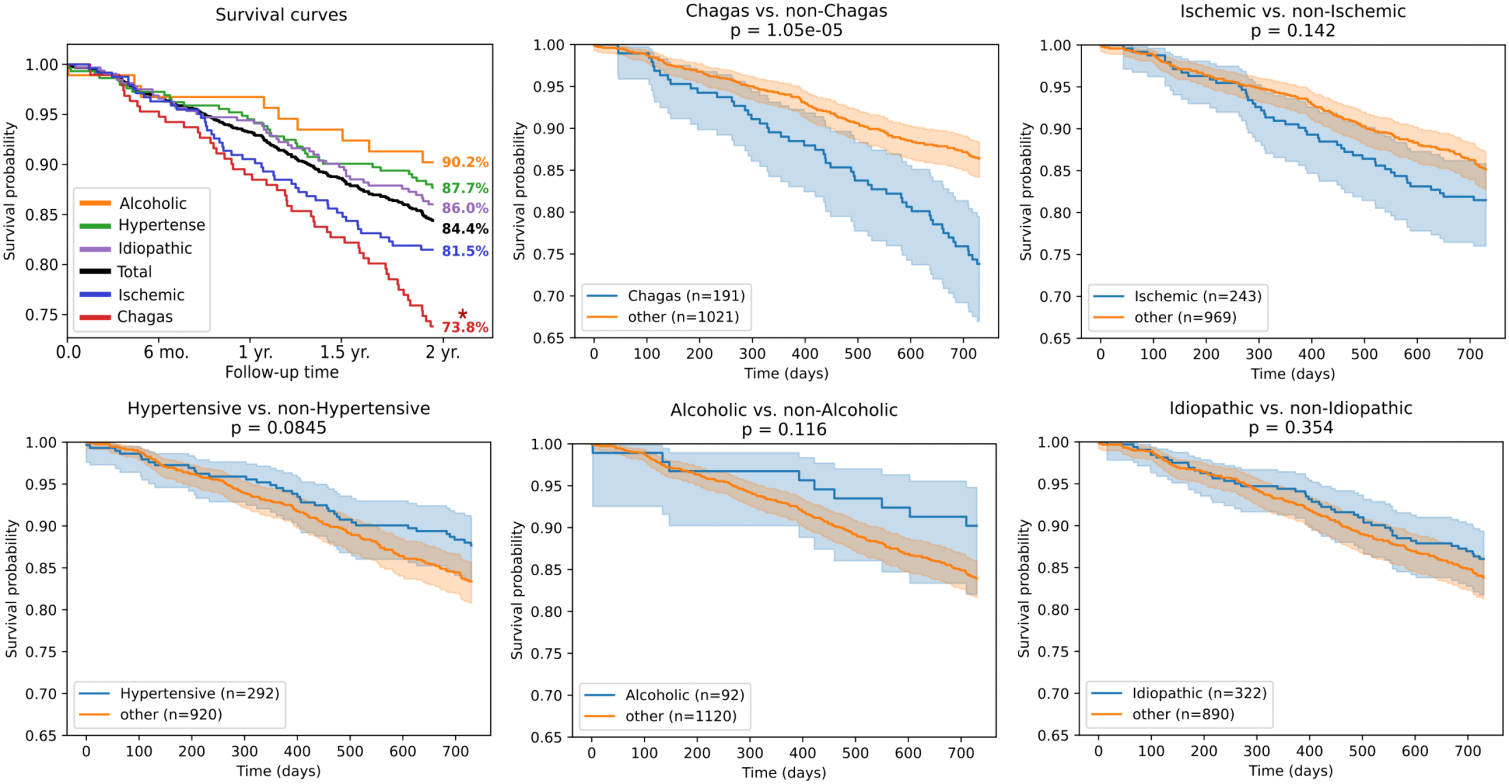
Kaplan–Meier survival by heart failure etiology. Kaplan-Meier survival by heart failure etiology. **(A)** Overall two-year survival curves for all etiologic subgroups (idiopathic, hypertensive, ischemic, Chagas cardiomyopathy, alcoholic, and the whole set of patients), with proportion of deaths shown to the right of each curve; significance (“*”) assessed by log-rank test. **(B)** Survival curve comparing Chagas versus non-Chagas etiology, demonstrating a significantly steeper decline in Chagas patients (p = 1.05e-05). **(C–F)** Separate survival comparisons for each non-Chagas etiology versus the remaining cohort: ischemic, hypertensive, alcoholic, and idiopathic HF curves.

Kaplan–Meier curves (Fig 1) revealed marked heterogeneity in survival across heart failure etiologies. CCC showed the lowest two-year survival (74%), significantly lower than ischemic (81%), idiopathic (86%), hypertensive (88%), and alcoholic (90%) HF (Fig 1A). Direct comparison between CCC and non-CCC patients confirmed a significantly steeper decline in survival for CCC (*p* = 1.05 × 10 ⁻ ⁵), whereas the survival curves of the remaining etiologies did not significantly differ from that of the overall cohort (Fig 1B–1F). A detailed survival risk table, reporting the number of patients at risk from baseline through the entire follow-up period at six-month intervals, is provided in S2 Table.

### Proteomic panel QC

A total of 98% of the datapoints passed QC for the Cardiometabolic panel and 97% for the Inflammation panel, with an average intra-assay coefficient of variation (CV) of 9% and inter-assay CV of 17%. High-throughput profiling of 734 plasma proteins revealed marked heterogeneity. Markers from the Inflammation and Cardiometabolic panels were broadly intermixed after PCA, with no clear separation or clustering by panel, suggesting that panel-related batch effects are not a major contributor to the observed variability in the data (S1 Fig).

### Etiology-specific proteins

S3 Table and S2 Fig (etiology-associated markers) showed CCC had the largest number of uniquely associated proteins (128), followed by hypertensive [41] and ischemic [24] HF; idiopathic and alcoholic groups displayed no significant markers.

### Machine learning model

Although the naive Bayes model initially showed slightly better performance (S3A Fig), we ultimately selected logistic regression for the final analyses. Naive Bayes assumes conditional independence among predictors, an assumption rarely met in biological datasets, potentially leading to biased probability estimates. In contrast, logistic regression directly models the conditional probability of the outcome and is generally more robust to correlated features while providing better-calibrated risk estimates. Accordingly, all downstream analyses were conducted using logistic regression. For the final evaluation on the test set, confidence intervals were estimated using 100 bootstrap resamples to assess model performance. When all ten machine learning models were evaluated using the final marker set obtained after feature selection, logistic regression was identified as a strong-performing model across three different metrics (S3B Fig).

### P9: A generalizable HFrEF model

We evaluated mortality prediction using the full proteomic dataset and progressively sparser protein subsets. The feature-selection workflow, combining a genetic algorithm followed by Bayesian stochastic search variable selection (feature-selection code in S2–S3 Files), is illustrated in S4 Fig. The GA step first reduced the feature space to 322 candidate proteins (S4A Fig), which were subsequently ranked using SSVS (S4B Fig). Model performance was then assessed by F1-macro using progressively larger subsets, starting with the top-ranked protein and expanding sequentially up to the top 50 proteins (S4C Fig).

**Fig 2:**
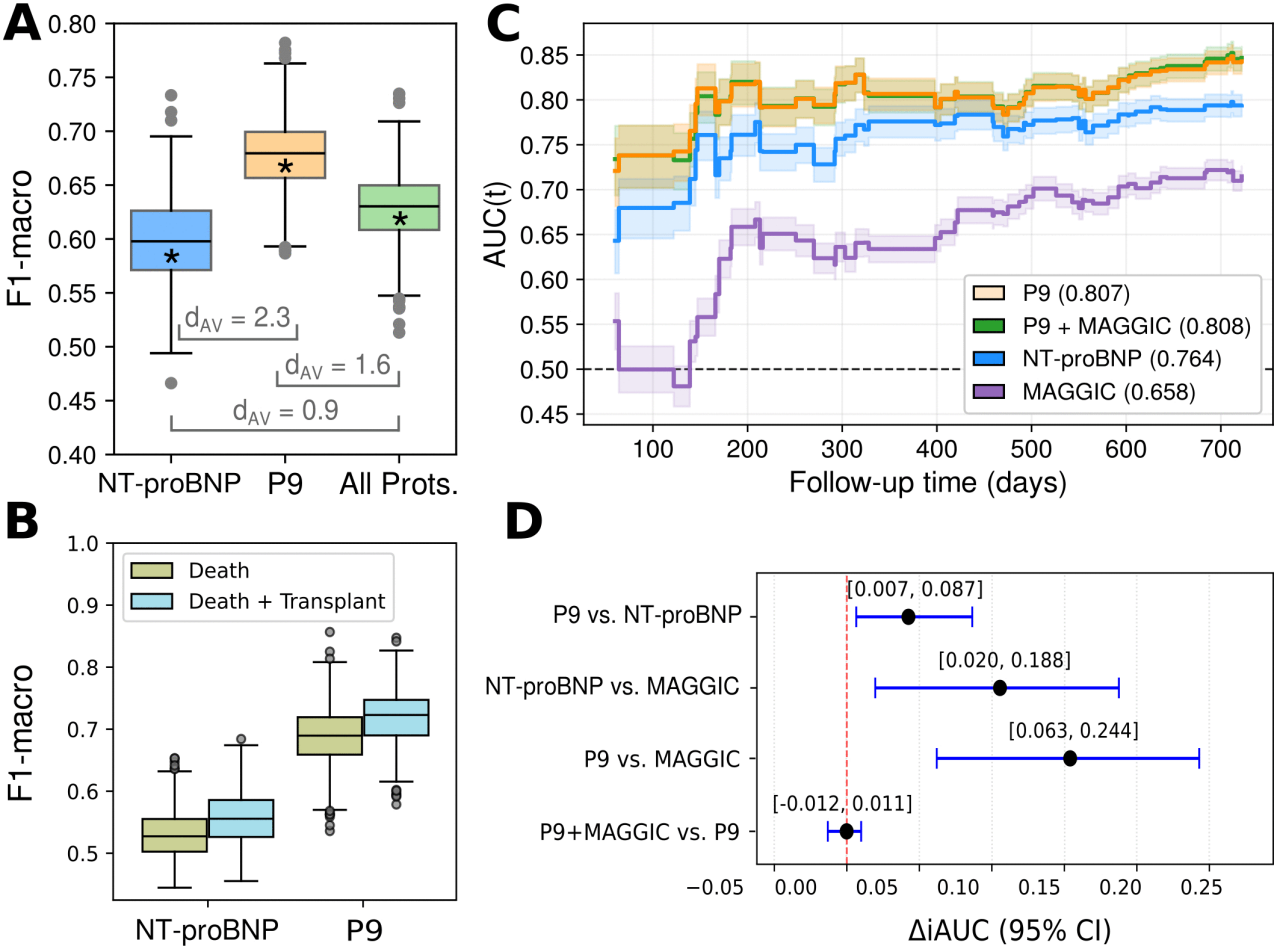
Distribution of predictive performance across biomarker models. Distribution of predictive performance across biomarker models. **(A)** Boxplots show the F1-macro scores obtained from 1,000 random train-validation permutations (70/30 split, employing only the original train set) using logistic regression classifiers built with three feature sets: NT-proBNP (“BNP”), the 9-protein panel (“P9”), and the full proteomic panel with 734 proteins (“All Prots.”). **(B)** Predictive performance for the endpoint of all-cause mortality (“Death”) and for the composite outcome of death or heart transplantation between marker sets. Boxplots depict the distribution of F1-macro scores across 1,000 test set bootstraps using logistic regression models built with NT-proBNP (BNP) and the 9-protein panel (P9). A two one-sided tests (TOST) procedure was applied to formally assess equivalence between models for the same outcome (distributions were not significantly equivalent). **(C)** Time-dependent discrimination of biomarker models P9, P9 + MAGGIC, BNP, and MAGGIC for right-censored Cox proportional hazards survival prediction in the test set, under a cumulative/dynamic framework. Stepwise curves show AUC(t) with 95% highest density intervals (HDI) derived from 1,000 bootstraps, restricted to events occurring ≥ 50 days. **(D)** Forest plot comparing iAUC between models in the test set. Horizontal bars denote 95% highest density intervals (HDI), deemed significant in case they exclude zero (red dotted line).

This process identified a nine-protein panel P9 (C1QA, CCL4, REN, EGLN1, COL9A1, GP1BA, ITM2A, CNPY2, and NT-proBNP), that achieved significantly higher F1-macro for 2-year mortality in the validation set than either NT-proBNP alone or the full 734-protein model (Fig 2A). Estimated paired-sample effect sizes (Cohen’s d_AV_) [42,43] indicated large differences between models: P9 vs. NT-proBNP = 2.3; P9 vs. all proteins = 1.6; all proteins vs. NT-proBNP = 0.9 (Fig 2A). A comparison of P9 against other random subsets of nine proteins (one of them always being NT-proBNP) in the train set showed that its F1-macro was indeed superior to any of the 9-marker-sets tested (S4D Fig). Standardized coefficients for all nine markers of the P9 model were also obtained, highlighting the relative importance of each protein to the model (S4E Fig).

The P9 panel also showed superior performance in the independent test set for both mortality and the composite outcome of mortality or heart transplantation (Fig 2B). Bootstrap optimism correction for P9 demonstrated limited overfitting, as evidenced by the small correction obtained (averaged ΔF1-macro difference of 0.013, based on a 1,000 bootstrap resampling strategy). The test set performance (0.674) was within the corrected bootstrap estimate (95% IC = [0.603, 0.699]).

We also observed similar performance between mortality alone and the composite outcome for both NT-proBNP and P9 models, suggesting robustness of the panel across outcomes. However, because there were only 14 heart transplantation cases for the composite endpoint, subsequent analyses focused on mortality alone.

Time-dependent discrimination analyses demonstrated that P9 improved integrated AUC (iAUC) compared with both NT-proBNP (+5.6%; ΔiAUC = 0.043 [0.007–0.087]) and the MAGGIC clinical score (+23.8%; ΔiAUC = 0.155 [0.063–0.244]) (Fig 2C–2D). Adding MAGGIC to the P9 model did not further improve discrimination (ΔiAUC = 0.001 [−0.012–0.011]), indicating that clinical variables provided little incremental value beyond the P9 proteomic panel.

Event-per-variable estimates indicated adequate statistical power for both models. The NT-proBNP model yielded an EPV of 47.25, and the P9 model an EPV of 15.75, both exceeding the commonly accepted threshold of 10 events per variable, suggesting adequate statistical power for model estimation.

Calibration metrics indicated better agreement between predicted and observed risks for the P9 model than for NT-proBNP alone. In the full cohort, P9 had CITL and calibration slope values closer to the ideal references of 0 and 1, respectively (CITL: −0.210 vs. −0.500; slope: 0.870 vs. 0.663). In the Chagas subgroup, both models showed positive CITL values and slopes >1, suggesting systematic calibration deviation with under-dispersed predictions. In the non-Chagas subgroup, both models showed negative CITL values and slopes <1, indicating risk miscalibration with overfitted predictions; however, these deviations were less pronounced for P9 (CITL = −0.463; slope = 0.761) than for NT-proBNP alone (CITL = −0.833; slope = 0.514) (S5A Fig).

Nevertheless, calibration did not improve model performance relative to the raw models. The raw P9 model showed the lowest Brier score (0.0977), slightly better than the calibrated P9 (0.0984) and both NT-proBNP versions (raw: 0.1116; calibrated: 0.1119) (S5B–S5C Fig). In Decision Curve Analysis, the P9 model consistently provided greater net benefit than NT-proBNP and the default strategies of treating all or no patients (S5D Fig). Although NT-proBNP showed modest benefit over the “treat all” strategy at lower thresholds, it remained inferior to P9 across most clinically relevant ranges.

Comparing threshold probabilities between 5% and 40%, P9 demonstrated a positive Δ net benefit relative to NT-proBNP, with 95% bootstrap confidence intervals (1,000 resamples) remaining above zero for most thresholds between 10% and 35% (S5E Fig). The largest gains occurred between 25% and 35%. Trade-off estimates indicated that 26–65 additional patients would need to be evaluated with P9 rather than NT-proBNP to correctly identify one additional true positive (death within two years) within this threshold range. Because calibration did not improve model performance, all subsequent analyses were conducted using the raw P9 and NT-proBNP models.

**Fig 3:**
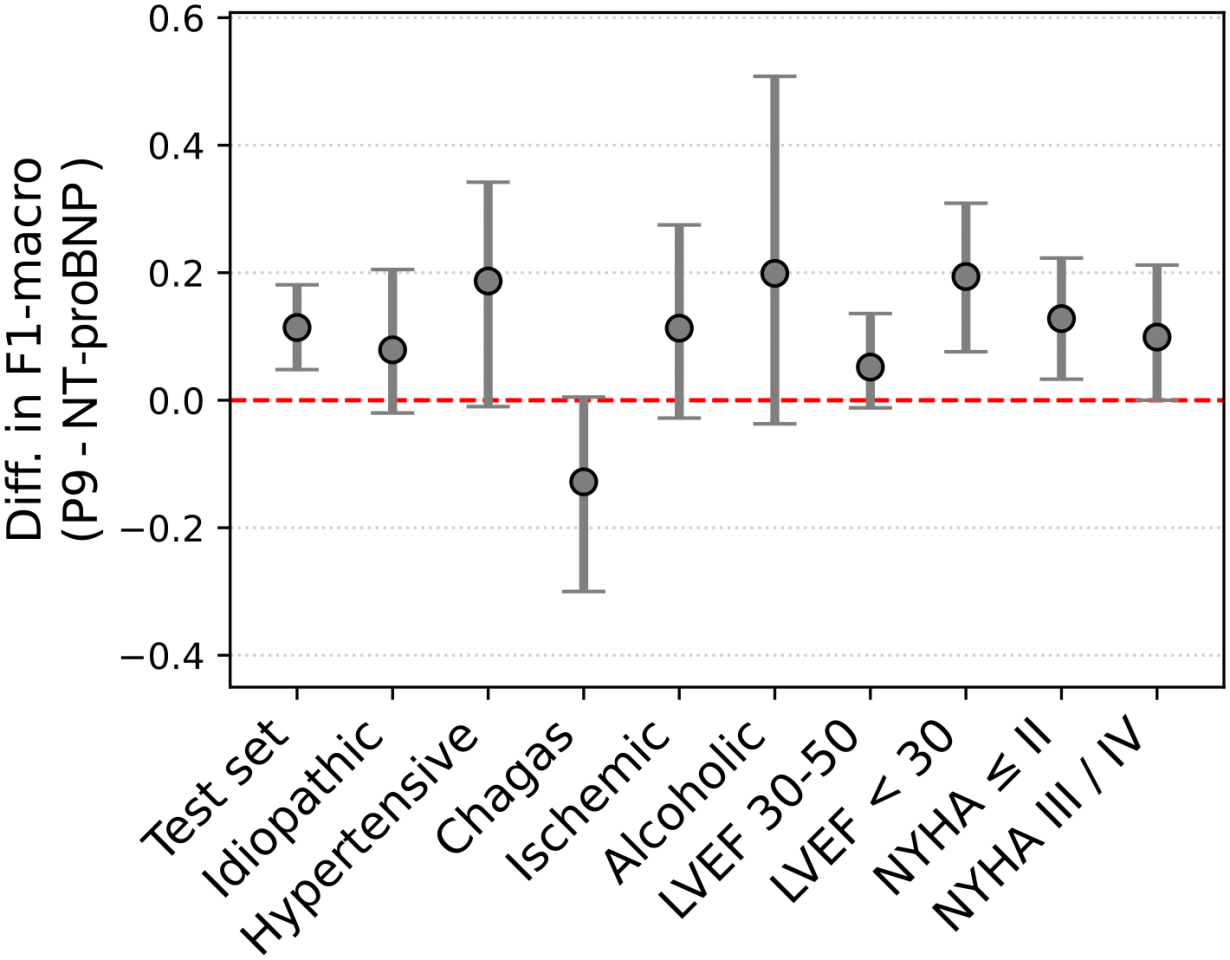
Subgroup analysis of predictive performance differences between the P9 panel and NT-proBNP. Subgroup analysis of predictive performance differences between the P9 panel and NT-proBNP models. Points represent the absolute difference in F1-macro (P9 minus BNP) across the overall test set and clinically relevant subgroups, including etiology, LVEF strata, and NYHA class. Vertical error bars denote 95% confidence intervals based on 1,000 bootstraps on test set. Interrogation mark denotes small power due to sample size (alcoholic on test set: N = 31, deaths = 3).

Fig 3 presents a graphical comparison of F1-macro 95% confidence intervals across etiologies, highlighting the distinct pattern observed in CCC, which contrasts with other HF classes. As shown in Table 2, the P9 panel remained informative in CCC (F1-macro = 0.685), whereas NT-proBNP exhibited unusually higher classification performance in this subgroup (F1-macro = 0.813) relative to other etiologies.

**Table 2:** Discriminatory performance of the P9 panel compared with BNP model.

	F1-macro							iAUC						
	BNP	P9	Δ F1-macro (95%)	% F1-macro increase	P-value	N (train/test)	deaths (train/test)	BNP	P9	Δ iAUC (95%)	% iAUC increase	P-value	N (train/test)	deaths (train/test)
***Test set (unseen data)***	0,560	0,674	0.114 [0.048, 0.181]	20,35%	0.000*	848/364	132/57	0,764	0,807	0.043 [0.007, 0.087]	5,63%	0.018*	848/364	132/57
***Etiologies***														
Idiopathic	0,512	0,591	0.079 [-0.020, 0.205]	15,43%	0,138	848/100	132/16	0,677	0,779	0.102 [0.012, 0.167]	15,07%	0.005*	848/100	132/16
Hypertensive	0,468	0,655	0.187 [-0.010, 0.342]	39,96%	0.046*	848/84	132/10	0,778	0,759	-0.019 [-0.119, 0.049]	-2,44%	0,681	848/84	132/10
Chagas	0,813	0,685	-0.128 [-0.300, 0.005]	-15,74%	0.041*	848/62	132/15	0,928	0,922	-0.006 [-0.081, 0.060]	-0,65%	0,540	848/62	132/15
Ischemic	0,531	0,644	0.113 [-0.028, 0.275]	21,28%	0,111	848/66	132/10	0,721	0,755	0.034 [-0.039, 0.141]	4,72%	0,189	848/66	132/10
Alcoholic	0,475	0,674	0.199 [-0.037, 0.508]	41,89%	0,366	848/31	132/3	0,847	0,909	0.062 [-0.028, 0.237]	7,32%	0,083	848/31	132/3
***LVEF***														
30-50	0,540	0,592	0.052 [-0.012, 0.136]	9,63%	0,138	848/232	132/26	0,725	0,793	0.068 [-0.009, 0.137]	9,38%	0.040*	848/232	132/26
< 30	0,534	0,728	0.194 [0.076, 0.309]	36,33%	0.001*	848/132	132/31	0,786	0,841	0.055 [0.004, 0.105]	7,00%	0.017*	848/132	132/31
***NYHA***														
≤ II	0,501	0,629	0.128 [0.033, 0.223]	25,55%	0.004*	842/295	132/29	0,754	0,814	0.060 [-0.003, 0.118]	7,96%	0.025*	842/295	132/29
III/IV	0,585	0,684	0.099 [0.000, 0.212]	16,92%	0.026*	842/63	132/26	0,718	0,722	0.004 [-0.048, 0.061]	0,56%	0,451	842/63	132/26

Discriminatory performance of the P9 panel compared with BNP model across the overall test cohort and clinically relevant subgroups. F1-macro (mean of F1-scores between deceased and alive, treating minority and majority groups with equal importance regardless of their frequency in the data) and integrated Area Under the Curve (iAUC) are shown for each data partition, along with the absolute differences (Δ F1-macro, Δ iAUC), respective 1,000 bootstrap-associated 95% highest density intervals (95% HDI), and unidirectional p-values for P(BNP ≥ P9) in the test set. Subgroup analyses included etiology, left ventricular ejection fraction (LVEF) classes, and NYHA functional categories.

S6 Fig presents time-dependent cumulative/dynamic ROC curves at 6 months, 1 year, and 2 years, further confirming the superior discrimination of P9 compared with NT-proBNP for mortality prediction. The AUC at 2 years and the integrated AUC across the full follow-up (Fig 2 and Table 2) differ slightly because AUC(t) reflects model performance at a specific time point, whereas iAUC represents a weighted average of discrimination across all time points up to the two-year horizon.

We then evaluated whether additional covariates could improve the P9 model. Including equivalized income as a covariate increased the F1-macro in the test set to 0.688, a 1.5% improvement, suggesting that socioeconomic variables may modestly enhance performance when available. A smaller effect was observed in the UKBB cohort, where standardized income increased F1-macro by 0.9% (0.575 to 0.580). In contrast, adding NYHA functional class produced negligible changes, affecting F1-macro only at the fourth decimal place.

Finally, we examined whether estimated glomerular filtration rate (eGFR), a well-established prognostic marker in HF, could improve prediction. As shown in S7 Fig, eGFR alone demonstrated modest discrimination (iAUC = 0.677), substantially lower than P9 (iAUC = 0.807). Although eGFR was statistically inferior to P9 (Cohen’s d_AV_ = 0.955; p < 0.001), combining the two did not improve performance, as the P9 + eGFR model closely overlapped with P9 alone. These results suggest that the P9 panel already captures much of the prognostic information associated with renal function, supporting its robustness as a generalizable HFrEF model.

### External validation

A total of 431 UKBB participants with prevalent HF at baseline were included (S4 Table), of whom 28 died within two years. The same covariates used in the primary analyses were applied, except for LVEF, which is not available at baseline in UKBB. To ensure comparability, the BNP and P9 models were refitted in the GENIUS-HF training set using only age and sex as covariates.

**Fig 4:**
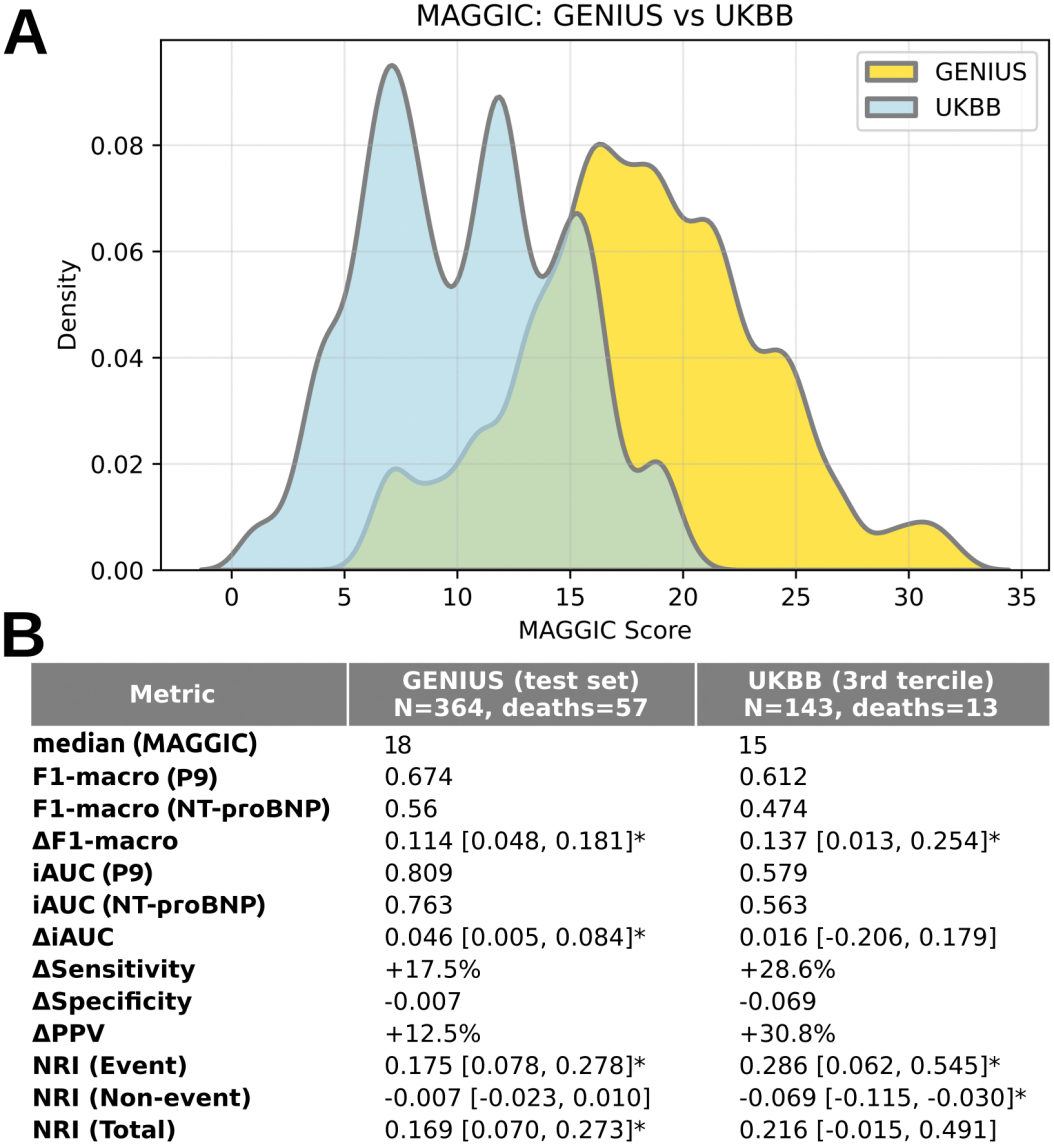
UK Biobank (UKBB) compared to GENIUS-HF. UK Biobank (UKBB) compared to GENIUS-HF, according to the MAGGIC score per sample; left ventricular ejection fraction (LVEF) was excluded from the analysis as a covariate because data were missing for more than 90% of the UKBB participants. **(A)** Distribution of UKBB HFrEF cohort (left) against GENIUS-HF (right). A total of 431 HFrEF samples (total deaths = 28) were found for UKBB; for the test set of GENIUS-HF the total is 364 samples (57 deaths). **(B)** Comparative classification performance of P9 vs. BNP models in the GENIUS-HF and UKBB cohorts, analyzed overall and within the highest-risk (3rd) MAGGIC tercile. Metrics include F1-macro, iAUC, sensitivity, specificity, positive predictive value (PPV), and net reclassification improvement (NRI total, by event, and by non-event). Δ values represent the change in performance achieved by P9 relative to BNP. Asterisks denote statistical significance based on 1,000 bootstrap-based 95% confidence intervals excluding zero.

Baseline clinical risk differed markedly between the GENIUS-HF and UKBB cohorts (Fig 4A–4B). Density plots showed a clear rightward shift of MAGGIC scores in GENIUS-HF, indicating a greater concentration of patients in higher predicted mortality strata (Fig 4A). In the GENIUS-HF test set (N = 364; 57 deaths), the median MAGGIC score was 18, consistent with a high-risk clinical HF population. In contrast, the UKBB cohort (N = 431; 28 deaths) had substantially lower baseline risk, with a median MAGGIC score of 10 (Fig 4A and S5 Table).

To improve comparability, external validation in UKBB was restricted to the highest-risk subgroup (third MAGGIC tertile; median = 15), which more closely approximates the risk distribution observed in GENIUS-HF (Fig 4A–4B). In this subset, P9 improved classification relative to BNP, with F1-macro scores of 0.612 vs. 0.474 (ΔF1-macro = 0.137; 95% CI: 0.013–0.254), corresponding to a 28% performance increase. Discrimination was similar between models (iAUC 0.579 for P9 vs. 0.563 for BNP; ΔiAUC = 0.016; 95% CI: −0.206–0.179). However, P9 substantially increased sensitivity (+28.6%) and positive predictive value (+30.8%), with a modest reduction in specificity (−6.9%).

Reclassification analyses showed improved identification of events (event NRI = 0.286; 95% CI: 0.062–0.545), partially offset by misclassification of non-events (non-event NRI = −0.069; 95% CI: −0.115 to −0.030), yielding a positive but non-significant total NRI (0.216; 95% CI: −0.015–0.491).

Overall, these findings indicate that the P9 proteomic signature improves mortality risk classification compared with BNP in a clinical HF cohort and demonstrates consistent external performance in an independent population-based cohort when analyses are restricted to patients with comparable baseline risk.

### Chagas: A unique HF case

The finding that NT-proBNP outperformed P9 in CCC (Fig 3 and Table 2), in contrast to all other HF etiologies, further supports the notion that Chagas cardiomyopathy has distinct pathophysiological features. Consistent with this observation, the volcano and upset plots (S2 Fig) showed that CCC had the largest number of uniquely significant proteins (n = 128), exceeding all other etiologies. This distinctive proteomic profile highlights the unique biochemical landscape of CCC and provides an opportunity to identify mechanisms and pathways driving disease progression, as explored in the pathway enrichment analyses presented below.

### Etiology-specific pathway enrichment

**Fig 5:**
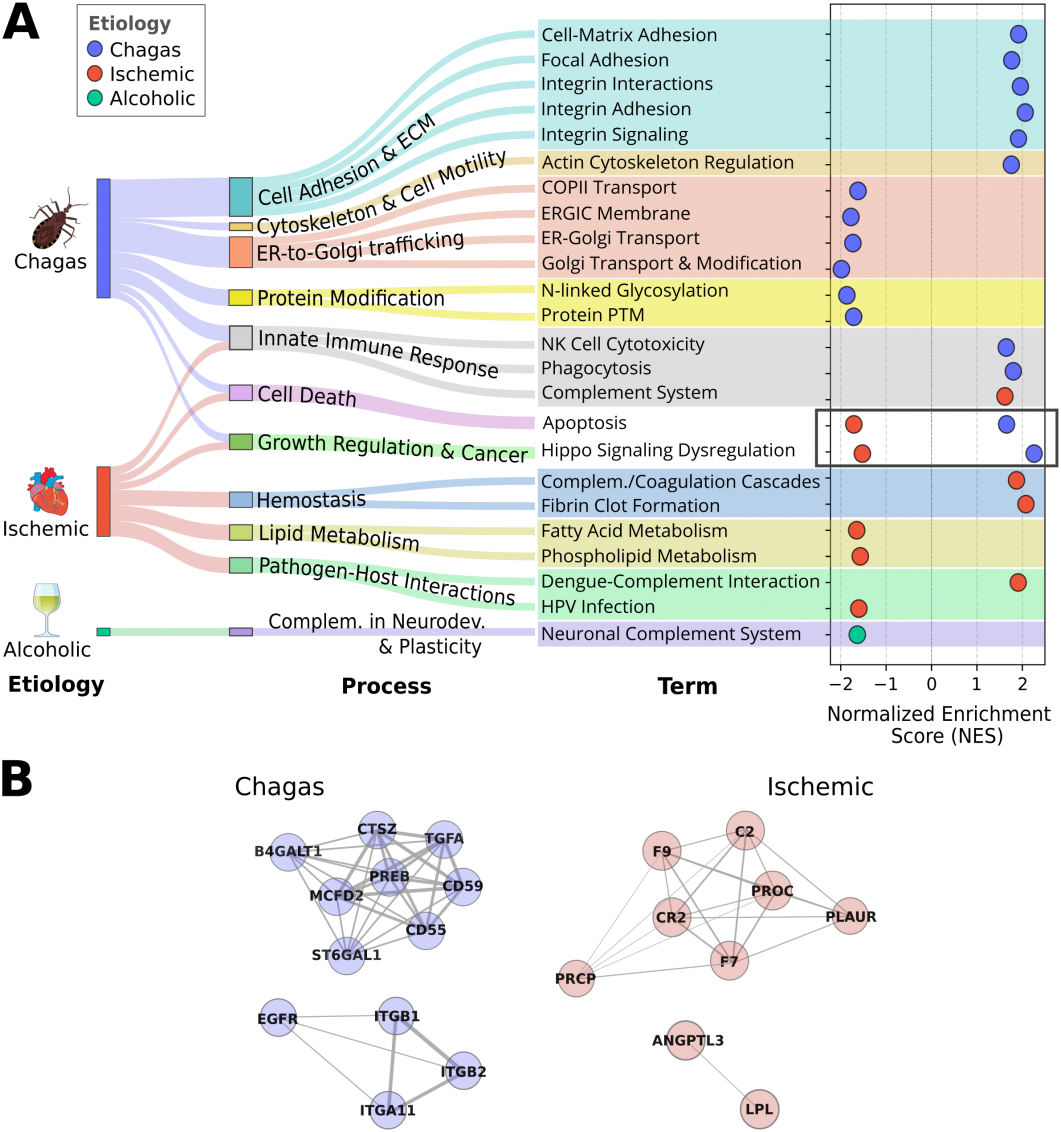
Enriched pathways from GSEA analysis of proteins. Enriched pathways from GSEA analysis of proteins. **(A)** Enriched pathways (“Term”) in the train set, that were further validated in the test set, for each etiology (hypertense and idiopathic groups had no validated pathways); main higher-level metabolic processes to the left (“Process”). NES values (normalized enrichment score, degree to which a term is over- or under-represented) to the right. **(B)** Network of genes present in more than one significant enriched pathway (“Term”) for Chagas and ischemic patients.

Gene set enrichment analysis (GSEA) identified 14 CCC-specific low-level pathways that were significant in both the training and test sets (Fig 5 and S6 Table). These pathways clustered into five higher-level functional categories: Cell Adhesion and Extracellular Matrix (cell–matrix adhesion, focal adhesion, integrin interactions, integrin adhesion, integrin signaling); Cytoskeleton and Cell Motility (actin cytoskeleton regulation); ER-to-Golgi trafficking (COPII transport, ERGIC membrane, ER–Golgi transport, Golgi transport and modification); Protein Modification (N-linked glycosylation, post-translational modification); and Innate Immune Response (NK cell cytotoxicity, phagocytosis). Pathways related to cell adhesion/extracellular matrix, cytoskeleton dynamics, and innate immune response were upregulated in CCC, whereas ER-to-Golgi trafficking and protein modification pathways were downregulated (Fig 5A).

In contrast, the ischemic-specific pathway Hemostasis (complement/coagulation cascades and fibrin clot formation) was upregulated, while Lipid Metabolism (fatty acid and phospholipid metabolism) was downregulated. Two Pathogen–Host Interaction pathways (Dengue–complement interaction and HPV infection) showed opposite enrichment directions (Fig 5A). Two low-level pathways, apoptosis and Hippo signaling dysregulation, were shared between CCC and ischemic HF but displayed opposite normalized enrichment score directions across etiologies (Fig 5A). The neuronal complement system pathway was downregulated in the alcoholic HF group; however, this finding was not further analyzed due to limited statistical power (n = 31; 3 deaths). Finally, protein interaction networks were constructed for CCC and ischemic HF (Fig 5B). Nodes represent proteins participating in more than one significantly enriched low-level pathway, and edges connect proteins co-occurring across these pathways, highlighting shared functional hubs within each disease-specific network.

### Druggable targets for CCC

Genes identified in the CCC interaction network (Fig 5B) were evaluated for druggability and known drug–target interactions using multiple databases, including DGIdb, ChEMBL, Pharos, DrugBank, and the Therapeutic Target Database. The initial screening generated a comprehensive list of candidate compounds (S7 Table). This list was subsequently filtered to exclude agents unlikely to be mechanistically relevant to the pathways identified in Fig 5 (e.g., laxatives, topical steroids, antimicrobials without cardiac relevance, insulin). The filtered results, summarizing the most biologically plausible targets and compounds, are presented in Table 3. Potential therapeutic repurposing candidates include cetuximab and panitumumab targeting fibrosis-related pathways, tretinoin associated with integrin signaling modulation, and prednisone, potentially relevant in the context of broader immunomodulation in CCC.

**Table 3:** Proteins in enriched Chagas pathways and associated putative therapeutical compounds.

Target/Pathway	Therapeutic Agent	Priority in Chagas HFrEF	Pros	Cons
Fibrosis (EGFR/TGFA)	*Cetuximab*/*Panitumumab*	High priority (Fibrosis-driven remodeling)	• Attenuates adverse remodeling • Preclinical cardiac evidence	• Cardiotoxicity risk (LV dysfunction) • High cost
Integrin Signaling (ITGB2/CD55)	*Tretinoin* (vitamin A)	High priority (Anti-fibrotic, immunomodulatory)	• Oral administration • Established safety profile • Reduces collagen deposition	• Limited clinical data in HF • Potential teratogenicity
Broad Immunosuppression	*Prednisone*	Limited role (only if autoimmune component confirmed)	• Rapid anti-inflammatory effects	• Worsens fluid retention • May accelerate myocardial damage

Consolidated information regarding combinations of proteins in enriched Chagas pathways and associated putative therapeutical compounds.

## Discussion

Heart failure remains a major cause of morbidity and mortality worldwide, and the accurate prediction of outcomes is critical for guiding management strategies. BNP and NT-proBNP have long been the gold-standard biomarkers for prognostication in HF across etiologies [44,45]. However, these markers often fail to capture etiology-specific pathophysiological nuances, particularly in non-ischemic HF syndromes. In this study, we show that proteomic-based models substantially improve mortality prediction in most HF subtypes, but with an important exception in CCC, where NT-proBNP alone performed better than the nine-protein model, P9. This finding underscores the singularity of CCC as a unique HF entity and provide a mechanistic rationale for why CCC may not conform to generalized HF biomarker models and open a window for etiology-specific diagnostic, prognostic, and therapeutic strategies.

**P9 performance in overall HF:** The improved predictive performance of the P9 panel over NT-proBNP across most HF etiologies prompted further evaluation of model reliability and clinical utility through calibration and decision-analytic performance.

Regarding calibration, the lower classification error observed with the raw NT-proBNP and P9 models compared with calibrated versions likely reflects the machine-learning framework used. Logistic regression employs log-odds through the canonical logit link and is trained by minimizing log-loss (cross-entropy), a proper scoring rule that promotes well-calibrated probability estimates. Empirical studies support this property: Niculescu-Mizil and Caruana [46] showed that logistic regression models are typically well calibrated without post-hoc adjustment, while Gutman et al. [47] demonstrated that optimization of log-loss under the canonical link confers the balance property, making well-specified models intrinsically calibrated. Consistent with this principle, raw models performed as well as, or slightly better than, isotonic-calibrated counterparts based on Brier score, suggesting that additional recalibration may introduce variance without improving probabilistic accuracy.

Decision Curve Analysis (DCA) showed that P9 consistently outperformed NT-proBNP across clinically relevant threshold probabilities. Within the 10–35% range, where P9 yielded positive Δ net benefit, the number of additional patients needing evaluation with P9 rather than NT-proBNP to correctly identify one additional death within two years ranged from 63 (10% threshold) to 26 (35% threshold). These findings indicate that incorporating P9 improves risk stratification and may support more individualized clinical decision-making in HFrEF.

Importantly, the superiority of P9 over NT-proBNP was also observed in the independent UKBB validation dataset, supporting robustness and generalizability. Although overall performance metrics were lower in UKBB, consistent with its community-based design and lower disease severity, P9 still achieved higher F1-macro scores, sensitivity, and positive predictive value than NT-proBNP alone. These findings support the clinical relevance of the P9 panel for risk stratification across diverse populations and may contribute to more efficient allocation of healthcare resources, particularly in universal health systems in low- and middle-income countries such as Brazil.

**Differences in performance between GENIUS-HF and UKBB and interpretation of F1-macro vs. iAUC:** The magnitude of improvement achieved by the P9 proteomic model differed between GENIUS-HF and UKBB, likely reflecting differences in baseline risk and event rates. GENIUS-HF represents a clinical HF population with higher risk (median MAGGIC score 18) and more events, conditions in which biomarker models typically show stronger discrimination because disease-related biological signals are more pronounced. In contrast, UKBB is population-based and healthier (median MAGGIC score 10), with fewer deaths and a narrower risk spectrum, producing a lower signal-to-noise ratio for biomarker prediction. The smaller number of events in UKBB also reduces statistical power to detect differences in iAUC, particularly given the event-to-variable requirements of Cox models.

The discrepancy between larger improvements in F1-macro and smaller changes in iAUC reflects the different performance dimensions captured by these metrics. iAUC evaluates global risk ranking across thresholds and is therefore relatively insensitive to improvements occurring primarily at clinically relevant decision points, especially in imbalanced datasets such as mortality prediction. By contrast, F1-macro combines precision and recall and directly assesses classification performance for both outcome classes, making it more sensitive to improvements in identifying deaths. In both cohorts, P9 primarily increased sensitivity and positive predictive value for patients who died within two years, yielding meaningful gains in F1-macro and event-specific NRI despite modest changes in global risk ordering.

**Linking the proteomic signature to heart failure biology:** The improved prognostic performance of the P9 panel likely reflects its ability to capture multiple biological pathways driving HF progression beyond the hemodynamic stress signal represented by NT-proBNP. While NT-proBNP reflects myocardial stretch and volume overload, circulating proteomic profiles integrate signals from several interconnected processes contributing to adverse cardiac remodeling and systemic deterioration. These include extracellular matrix remodeling, inflammation and immune activation, endothelial dysfunction, impaired cellular communication, and altered protein trafficking and metabolic stress within cardiomyocytes.

These mechanisms promote progressive myocardial dysfunction, arrhythmogenic substrate formation, and multiorgan involvement, increasing mortality risk. Because these processes evolve differently across disease stages and HF etiologies, multiplex proteomic signatures such as P9 may better represent the underlying disease state than single biomarkers. This systems-level capture of pathophysiology likely explains P9’s improved identification of patients at highest mortality risk, particularly those with ongoing remodeling and inflammatory activity despite similar clinical risk scores. Proteomic-based models may therefore represent an important step toward biologically informed risk stratification in HF.

**P9 biology and combined prognostic signal:** The nine-protein panel integrates complementary axes of HF biology beyond wall stress. NT-proBNP anchors the panel as a surrogate of ventricular stress and fibrosis-related load [48–51], while REN captures systemic and myocardial RAAS activation, a key driver of vasoconstriction and adverse remodeling [10]. EGLN1/PHD2 regulates HIF signaling, linking hypoxia to angiogenic and metabolic responses [50]. Immune activity is reflected by C1QA and CCL4, consistent with thrombo-inflammatory remodeling in HF and CCC [15,18,51]. GP1BA indexes platelet adhesion and thrombo-inflammatory risk following ischemic injury [51]. Structural remodeling is captured by COL9A1 and ITM2A, markers associated with extracellular matrix turnover and cellular differentiation programs [15]. Finally, CNPY2 reflects ER stress and protein trafficking processes relevant to circulating proteomic signals in advanced HF [18].

Together these proteins integrate wall stress, neurohormonal activation, hypoxia sensing, immune activation, thrombo-inflammation, and extracellular matrix remodeling. This multilayered representation of disease biology explains the improved prognostic performance of P9 in non-CCC HF etiologies.

The nine-protein panel integrates complementary axes of HF biology that extend beyond wall-stress alone and, together, sharpen mortality risk stratification. NT-proBNP anchors the panel as a robust surrogate of ventricular wall stress and fibrosis-driven load (EGLN1/PHD2 senses cellular oxygen tension and regulates HIF signaling, linking tissue hypoxia to angiogenic, metabolic, and fibrotic programs relevant to decompensation (**CCC as a Unique Case within HF:** CCC showed both higher mortality and the largest set of uniquely significant proteins (128), reinforcing its distinct biological profile. Pathway enrichment revealed CCC-specific processes including extracellular matrix remodeling, integrin-mediated adhesion, cytoskeletal regulation, and innate immune activation, consistent with the histopathological hallmarks of CCC, diffuse myocarditis, fibrosis, and chronic immune dysregulation [52,53]. Conversely, ER-to-Golgi trafficking and N-linked glycosylation pathways were downregulated, suggesting impaired protein processing and secretion.

Previous CCC biomarker studies have largely relied on targeted assays or alternative molecular approaches such as microRNA analysis, limiting direct comparison with our findings [54]. Nonetheless, a prior PBMC proteomic study similarly identified cytoskeletal disorganization and immune-cell activation pathways [55], supporting the central role of immune activation and structural remodeling in CCC.

**NT-proBNP retains predictive power in CCC through protein processing defects:** An intriguing observation was that P9 outperformed NT-proBNP in idiopathic, ischemic, and hypertensive HF but not in CCC. This may reflect the unique biochemical context revealed by pathway analysis. CCC displayed downregulation of ER-to-Golgi trafficking and N-linked glycosylation pathways, processes critical for the modification and secretion of many circulating proteins.

NT-proBNP secretion from ventricular cardiomyocytes appears relatively preserved despite trafficking disturbances, allowing it to maintain strong predictive power. In contrast, the broader proteomic signals captured by P9 depend on proper protein synthesis, modification, and secretion. Impairment of these processes in CCC may attenuate the incremental prognostic signal of multiplex proteomic markers.

These findings suggest that NT-proBNP retains particular value in CCC and highlight the importance of etiology-specific biomarker strategies. Integrating NT-proBNP with CCC-specific markers of fibrosis, integrin signaling, and immune activation may ultimately yield more effective prognostic tools.

**Comparison with ischemic HF:** Ischemic HF displayed enrichment of hemostasis and thrombo-inflammatory cascades alongside downregulation of lipid metabolism, consistent with the central role of coagulation-inflammation interplay and metabolic remodeling in ischemic disease [51,56]. Two pathways, apoptosis and Hippo signaling, were shared between CCC and ischemic HF but showed opposite enrichment directions, reinforcing divergent mechanisms despite similar clinical outcomes.

**Clinical implications and opportunities for diagnosis:** The combination of fibrosis, immune activation, and protein trafficking defects observed in CCC suggests actionable diagnostic opportunities. CCC-specific proteomic panels incorporating ECM/integrin, immune, and trafficking markers could complement imaging modalities sensitive to fibrosis and remodeling, such as CMR extracellular volume quantification or fibroblast-activation tracers. Because NT-proBNP retained strong predictive value in CCC, integrating it within CCC-specific biomarker panels may represent the most pragmatic approach.

**Therapeutic hypotheses and future directions:** Druggability analyses highlighted fibrosis- and immune-related pathways as potential therapeutic targets in CCC. EGFR-mediated remodeling emerged as a notable signal, consistent with its role in fibroblast activation and extracellular matrix deposition [57]. Pharmacologic EGFR inhibition through monoclonal antibodies such as cetuximab or panitumumab may represent a potential strategy to attenuate fibrotic remodeling, although translational investigation is required due to potential off-target effects.

Integrin-mediated fibrosis also emerged as an enriched axis. Tretinoin has been shown to modulate integrin signaling and reduce fibrosis in experimental models [58,59], suggesting potential therapeutic exploration. Dysregulated innate immune pathways further indicate opportunities for targeted immunomodulation.

These hypotheses remain exploratory and require rigorous preclinical validation before clinical investigation. Safety considerations are particularly important given potential cardiotoxicity of antitumoral agents and the risk of T. cruzi reactivation with immunosuppression.

**Novelty of findings:** This study provides several contributions. First, universal proteomic models outperform NT-proBNP across most HF etiologies. Second, CCC emerges as the only etiology in which NT-proBNP retains superior predictive performance. Third, we delineate key biological processes distinguishing CCC, including fibrosis, adhesion, immune activation, and trafficking defects. Finally, integrating proteomic enrichment with druggability databases generates testable therapeutic hypotheses for etiology-specific HF management.

**Limitations and future perspectives:** Limitations should be acknowledged. Although the cohort is large, the number of CCC patients remains smaller than other etiologies. Plasma proteomics reflects circulating signatures rather than tissue-level remodeling. The observational design precludes causal inference, and functional validation of candidate pathways is needed. In addition, EF < 50% was used to define HFrEF because patient inclusion preceded the recognition of HFmrEF [60]. Information on acute infection stage, prior treatment, or T. cruzi strain was unavailable.

These limitations highlight the need for further translational studies. Our findings position CCC as a biologically distinct HF entity and provide a roadmap for future biomarker development and mechanistic research.

## Conclusion

Altogether, these findings support the transition from universal biomarker strategies toward etiology-informed approaches in HF. The P9 proteomic panel improved risk prediction across several HF etiologies and demonstrated consistent performance in external validation using the UKBB populational cohort. In contrast, CCC displayed a distinct proteomic profile in which NT-proBNP retained strong prognostic value, suggesting that conventional models may not fully capture its biology. These results highlight the need for CCC-specific biomarker strategies and further mechanistic studies to advance precision medicine in heart failure.

## Supporting information

S1 Fig Principal component analysis (PCA) of proteomic markers vs. patients. Principal component analysis (PCA) of proteomic markers vs. patients, employing the two main principal components. Each point represents a marker (protein) from the train dataset, positioned according to how its expression pattern varies across the 848 patients after PCA dimension reduction. Points are colored according to the two Olink’s panel categories (Inflammation vs. Cardiometabolic).
(TIF)

S2 Fig Association of single proteins with etiologies. Association of single proteins with etiologies (based on the train set). (A-E) Volcano Plots with odds ratio in the x-axis, and -log10[adjusted p-value] in y-axis. Positive significant proteins in red, negative in blue. (F) Upset plot summarizing biomarker associations to one or more etiologies.
(TIF)

S3 Fig Distribution of F1-macro scores for each machine learning model. (A) Distribution of F1-macro scores for each machine learning model (ML) tested, presented as box plots. The evaluation methodology involved 1,000 random patient permutations in the Train set. For each run, 70% of patient data was randomly selected for model training, and the resulting F1-macro score was calculated on the held-out 30% validation set. Models are ranked top-to-bottom according to their median performance score. (B) Reassessing all ten ML models for P9 on the Test set for three different metrics (across 100 bootstraps).
(TIF)

S4 Fig Sequential machine learning pipeline for proteomic feature selection. (A) Genetic Algorithm-based logistic regression optimized F1-macro across 734 proteins using iterative selection, crossover, and mutation, yielding 322 pre-selected features. (B) Bayesian variable selection via Stochastic Search Variable Selection was applied to the reduced feature set using MCMC sampling, and proteins were ranked by Posterior Inclusion Probability (PIP). (C) Successively inclusive feature sets were evaluated to identify the optimal number of proteins maximizing F1-macro, resulting in a final panel of nine proteins (shaded area: permutation-based 95% CI). (D) P9 compared against 100 randomly generated protein sets composed of NT-proBNP plus eight randomly selected markers. For each set, logistic regression performance was evaluated across 10 independent stratified train/validation splits, and the median F1-macro recorded. (E) Stability of standardized logistic regression coefficients for the nine-protein panel across 1,000 repeated stratified resampling iterations. Coefficient distributions were summarized by median and 95% percentile intervals.
(TIF)

S5 Fig Clinical utility of P9 vs. BNP for 2-year mortality. (A) Calibration metrics for P9 and NT-proBNP in the overall study sample, Chagas subgroup and non-Chagas subgroup. (B) Calibration plot showing predicted versus observed mortality for the BNP model. The dotted diagonal line represents perfect calibration. The dotted red curve with squares shows raw predictions, while the solid blue curve with circles shows calibrated probabilities. Brier scores are also shown. (C) P9 Calibration plot, following the same strategy. (D) Decision Curve Analysis (DCA) comparing the P9 (orange) and BNP (blue) models. The “Treat All” strategy (red dotted line) starts and ends at the outcome prevalence (~16%), while the “Treat None” strategy (black line) remains at zero. A model is clinically useful when its curve sits above both baselines. (E) Δ Net Benefit across threshold probabilities: 95% CIs (shaded area) indicate significant superiority for P9 where the intervals exclude zero (based on 1,000 bootstraps). Numeric labels indicate the net reduction in unnecessary interventions per 100 patients achieved by using P9 instead of BNP without missing true cases. For example, at a 5% threshold, P9’s efficiency gain means avoiding 146.6 unnecessary interventions per 100 patients compared to BNP while maintaining equal safety.
(TIF)

S6 Fig Time-dependent cumulative/dynamic ROC curves for mortality prediction. Time-dependent cumulative/dynamic ROC curves for mortality prediction, using Cox proportional hazards models trained on the train set and validated on the test set, with predictive performance assessed at 6 months, 1 year, and 2 years of follow-up. The P9 Model (orange line) is compared against the BNP Model (blue line). Area under the curve (AUCt) metrics were estimated using the IPCW-based method of Uno’s et al. (2011) procedure to account for right-censoring, where sensitivity (TPR) and specificity (FPR) follow a cumulative/dynamic framework defining cases as events occurring up to time t, and controls as subjects remaining at risk beyond t. The grey dashed line represents the expected performance of a random risk classifier.
(TIF)

S7 Fig Effect of estimated glomerular filtration rate (eGFR). Effect of estimated glomerular filtration rate (eGFR) model compared to P9 model, with asymptotic influence-function variance and logit-transformed 95% confidence intervals (CI). Cohens’ d was calculated between P9 vs. eGFR, as the compound model “P9 + eGFR” bears no gains compared to P9 alone.
(TIF)

S1 Table Description of markers available from Olink. Description of markers available from Olink’s proteomic panels.
(XLSX)

S2 Table Survival risk table. Survival risk table, for the total patient set, and for individual etiology classes.
(XLSX)

S3 Table Exclusive significant proteins per etiology. Exclusive significant proteins per etiology, after FDR correction.
(XLSX)

S4 Table Descriptive statistics of UK Biobank. Descriptive statistics of UK Biobank (UKBB) individuals with Olink proteomic data and heart failure diagnosed before start of two-year follow-up. This is an external cohort to test for the general usefulness of P9 and NT-proBNP models.
(XLSX)

S5 Table UK Biobank (UKBB) compared to GENIUS-HF. UKBB compared to GENIUS-HF, with terciles according to the MAGGIC score per sample. Metrics include F1-macro, iAUC, sensitivity, specificity, positive predictive value (PPV), and net reclassification improvement (NRI total, by event, and by non-event). Δ values represent the change in performance achieved by P9 relative to BNP. Asterisks denote statistical significance based on 1,000 bootstrap-based 95% confidence intervals excluding zero.
(XLSX)

S6 Table Enriched pathways identified by etiology. Enriched pathways identified by etiology. Pathways significantly enriched in the training set were subsequently evaluated in the test set, and only those remaining significant in both datasets were retained for biological interpretation. The “Lead genes” column lists genes driving the enrichment signal that were consistently present in the significant pathways across both training and test sets.
(XLSX)

S7 Table First-pass results on putative repurposed drugs. First-pass results on putative repurposed drugs (before discarding “noisy” data).
(XLSX)

S1 File TRIPOD checklist file. Completed checklist according to the TRIPOD Statement (Transparent Reporting of a Multivariable Prediction Model for Individual Prognosis or Diagnosis), a reporting guideline designed to ensure transparent and complete reporting of studies describing the development and/or validation of multivariable prediction models. TRIPOD Checklist. Reproduced/adapted from the TRIPOD Statement (Transparent Reporting of a multivariable prediction model for Individual Prognosis Or Diagnosis), licensed under CC BY 4.0. Available at: https://www.tripod-statement.org/wp-content/uploads/2020/01/Tripod-Checlist-Prediction-Model-Development.pdf
(PDF)

S2 File Genetic Algorithm (GA) code. Feature selection (1st pass): Genetic Algorithm (GA) code.
(PY)

S3 File Stochastic Search Variable Selection (SSVS) code. Feature selection (2nd pass): Stochastic Search Variable Selection (SSVS) code.
(PY)
