# Supplementary material for: Plasma proteomics improves risk prediction in heart failure and reveals unique biology in chronic chagas cardiomyopathy
Source: PLoS Negl Trop Dis. 2026 Jun 8;20(6):e0014370. doi: 10.1371/journal.pntd.0014370 (PMC13245751; doi:10.1371/journal.pntd.0014370)
Supplement: S1 File — Completed checklist according to the TRIPOD Statement (Transparent Reporting of a Multivariable Prediction Model for Individual Prognosis or Diagnosis), a reporting guideline designed to ensure transparent and complete reporting of studies describing the development and/or validation of multivariable prediction models. TRIPOD Checklist. Reproduced/adapted from the TRIPOD Statement (Transparent Reporting of a multivariable prediction model for Individual Prognosis Or Diagnosis), licensed under CC BY 4.0. Available at: https://www.tripod-statement.org/wp-content/uploads/2020/01/Tripod-Checlist-Prediction-Model-Development.pdf (PDF) [file pntd.0014370.s015.pdf]

TRIPOD Checklist: Prediction Model Development and Validation

Section/Topic	Item		Checklist Item	Page
**Title and abstract**				
Title	1	D;V	Identify the study as developing and/or validating a multivariable prediction model, the target population, and the outcome to be predicted.	Title: Proteomic Risk Prediction in Chronic Chagas Cardiomyopathy Reveals Unique Biology. Target: HFrEF patients, including CCC. Outcome: 2-year mortality.
Abstract	2	D;V	Provide a summary of objectives, study design, setting, participants, sample size, predictors, outcome, statistical analysis, results, and conclusions.	Abstract includes objectives, design, setting, participants (n=1,212), predictors (734 proteins), outcome (2-year mortality), analyses (feature selection, Logistic Regression, Cox models), results (P9 improved prediction except in CCC), conclusions (etiology-specific biology).
**Introduction**				
Background and objectives	3a	D;V	Explain the medical context (including whether diagnostic or prognostic) and rationale for developing or validating the multivariable prediction model, including references to existing models.	HF prognosis improved for 4 etiologies; similar as BNP alone for CCC; proteomics improves death prediction overall.
	3b	D;V	Specify the objectives, including whether the study describes the development or validation of the model or both.	Develop proteomic models (P9), validate against BNP/MAGGIC, explore CCC-specific pathways.
**Methods**				
Source of data	4a	D;V	Describe the study design or source of data (e.g., randomized trial, cohort, or registry data), separately for the development and validation data sets, if applicable.	Single-center prospective cohort (GENIUS-HF, InCor, São Paulo, 2012–2015).
	4b	D;V	Specify the key study dates, including start of accrual; end of accrual; and, if applicable, end of follow-up.	Accrual: Aug 2012–Jan 2015; follow-up: 2 years.
Participants	5a	D;V	Specify key elements of the study setting (e.g., primary care, secondary care, general population) including number and location of centres.	Cardiology clinic, InCor/HCFMUSP, Brazil; n=1,212 patients with HFrEF.
	5b	D;V	Describe eligibility criteria for participants.	Adults with LVEF <50%, confirmed HF etiology; CCC confirmed by dual serology.
	5c	D;V	Give details of treatments received, if relevant.	Guideline-directed HF therapy >90% across groups.
Outcome	6a	D;V	Clearly define the outcome that is predicted by the prediction model, including how and when assessed.	All-cause mortality (or either death + heart transplantations) at 2 years, assessed via hospital records and national registry.
	6b	D;V	Report any actions to blind assessment of the outcome to be predicted.	Laboratory assays standardized; no blinding reported.
Predictors	7a	D;V	Clearly define all predictors used in developing or validating the multivariable prediction model, including how and when they were measured.	734 plasma proteins (Olink panels), plus age, sex, and LVEF as covariates.
	7b	D;V	Report any actions to blind assessment of predictors for the outcome and other predictors.	Laboratory assays standardized; no blinding reported.
Sample size	8	D;V	Explain how the study size was arrived at.	n=1,212; based on GENIUS-HF cohort enrollment in a 2-year follow-up.
Missing data	9	D;V	Describe how missing data were handled (e.g., complete-case analysis, single imputation, multiple imputation) with details of any imputation method.	Samples with <99% protein data excluded; missing values imputed by column medians.
Statistical analysis methods	10a	D	Describe how predictors were handled in the analyses.	Proteins z-scored; feature selection via genetic algorithm + stochastic search variable selection.
	10b	D	Specify type of model, all model-building procedures (including any predictor selection), and method for internal validation.	Feature selection; Logistic Regression classifier; Cox models; Validation via repeated shuffling and bootstrap.
	10c	V	For validation, describe how the predictions were calculated.	Predictions applied to independent 30% test set from same cohort, and to an external European cohort (UK BioBank).
	10d	D;V	Specify all measures used to assess model performance and, if relevant, to compare multiple models.	F1-macro, iAUC, Bootstraps, TOST equivalence, Cohen's d.
	10e	V	Describe any model updating (e.g., recalibration) arising from the validation, if done.	Recalibration employed, performing worse than raw model.
Risk groups	11	D;V	Provide details on how risk groups were created, if done.	Not applicable; continuous risk scores reported.
Development vs. validation	12	V	For validation, identify any differences from the development data in setting, eligibility criteria, outcome, and predictors.	Train/test split from same cohort; balanced by age, sex, LVEF, and BMI.
**Results**				
Participants	13a	D;V	Describe the flow of participants through the study, including the number of participants with and without the	n=1,212 enrolled; died=189; CCC n=191; 2-year mortality overall.

			outcome and, if applicable, a summary of the follow-up time. A diagram may be helpful.	16%, CCC 26%.
	13b	D;V	Describe the characteristics of the participants (basic demographics, clinical features, available predictors), including the number of participants with missing data for predictors and outcome.	CCC patients had lower BMI, fewer comorbidities, higher NYHA class, and lower income.
	13c	V	For validation, show a comparison with the development data of the distribution of important variables (demographics, predictors and outcome).	Train/test subsets balanced; CCC subgroup analyzed separately.
Model development	14a	D	Specify the number of participants and outcome events in each analysis.	1,212 patients; 191 CCC; deaths: 16% overall, 26% CCC.
	14b	D	If done, report the unadjusted association between each candidate predictor and outcome.	Logistic regression identified etiology-specific proteins; CCC had 128 unique markers.
Model specification	15a	D	Present the full prediction model to allow predictions for individuals (i.e., all regression coefficients, and model intercept or baseline survival at a given time point).	P9: C1QA, CCL4, REN, EGLN1, COL9A1, GP1BA, ITM2A, CNPY2, BNP; model type: Logistic Regression.
	15b	D	Explain how to the use the prediction model.	Apply Logistic Regression classifier in novel sample set with P9 inputs to predict 2-year mortality risk.
Model performance	16	D;V	Report performance measures (with CIs) for the prediction model.	P9 improved iAUC by 10.3% overall; CCC showed no gain over BNP; CIs reported where applicable.
Model-updating	17	V	If done, report the results from any model updating (i.e., model specification, model performance).	None.
**Discussion**				
Limitations	18	D;V	Discuss any limitations of the study (such as nonrepresentative sample, few events per predictor, missing data).	Modest CCC sample size; observational design; no causal inference.
Interpretation	19a	V	For validation, discuss the results with reference to performance in the development data, and any other validation data.	P9 outperformed BNP except in CCC.
	19b	D;V	Give an overall interpretation of the results, considering objectives, limitations, results from similar studies, and other relevant evidence.	A 9-Protein panel improves HF mortality prediction; CCC with unique biology across biochemical pathways; P9 retains predictive power overall in external cohorts.
Implications	20	D;V	Discuss the potential clinical use of the model and implications for future research.	HF mortality prediction improved with P9 model; CCC requires etiology-specific biomarkers; proteomics identifies putative therapeutic targets.
**Other information**				
Supplementary information	21	D;V	Provide information about the availability of supplementary resources, such as study protocol, Web calculator, and data sets.	Supplementary tables/figures provided; data available upon reasonable request, subject to approval from InCor.
Funding	22	D;V	Give the source of funding and the role of the funders for the present study.	FAPESP, CNPq, Zerbini Foundation, Foxconn Brazil; no funder role in study design or analysis.

*Items relevant only to the development of a prediction model are denoted by D, items relating solely to a validation of a prediction model are denoted by V, and items relating to both are denoted D;V. We recommend using the TRIPOD Checklist in conjunction with the TRIPOD Explanation and Elaboration document.
