# Supplementary material for: Plasma proteomics improves risk prediction in heart failure and reveals unique biology in chronic chagas cardiomyopathy
Source: PLoS Negl Trop Dis. 2026 Jun 8;20(6):e0014370. doi: 10.1371/journal.pntd.0014370 (PMC13245751; doi:10.1371/journal.pntd.0014370)
Supplement: Attachment — Submitted filename: 26_03_14_ResponsetoReview.pdf [file pntd.0014370.s018.pdf]

Response to Review

We thank the reviewers and editors for their careful evaluation of our manuscript and for their insightful comments and suggestions. The reviewers' feedback prompted important revisions that substantially strengthened the manuscript. In response, we have clarified several aspects of the study, expanded methodological descriptions, incorporated additional analyses and data, and included new analytical tools and figures to address the issues raised. These changes improved the transparency, robustness, and interpretation of the results. Below, we provide a detailed point-by-point response to each comment and describe the corresponding revisions made in the manuscript.

Editorial requirements:

1. **Please provide an Author Summary. This should appear in your manuscript between the Abstract (if applicable) and the Introduction, and should be 150-200 words long. The aim should be to make your findings accessible to a wide audience that includes both scientists and non-scientists. Sample summaries can be found on our website under Submission Guidelines:**

https://journals.plos.org/plosntds/s/submission-guidelines#loc-parts-of-a-submission

Author Summary.
Chagas disease remains a major cause of heart failure in Latin America and is increasingly recognized in other parts of the world. Heart failure occurs when the heart can no longer pump blood effectively, and doctors often rely on clinical scores and blood tests to estimate a patient's risk of complications or death. However, most of these tools were developed in patients with other types of heart failure and may not fully reflect the biological processes involved in Chagas cardiomyopathy. To address this question, we measured 734 proteins circulating in the blood of 1,212 patients with heart failure, including 191 with Chagas disease. We identified a group of nine proteins that improved prediction of death over two years in heart failure overall and confirmed this finding in an independent population cohort. When we examined patients with Chagas cardiomyopathy more closely, however, we observed a distinct set of protein signals linked to processes such as tissue scarring, immune system activation, and abnormalities in cellular protein handling. These results suggest that Chagas cardiomyopathy follows different biological pathways from other forms of heart failure and may require disease-specific biomarkers and treatment strategies. Understanding these differences may help guide more precise approaches to diagnosis and care for this neglected disease.

2. **We noticed that you used the phrase 'data not shown' in the manuscript. We do not allow these references, as the PLOS data access policy requires that all data be either published with the manuscript or made available in a publicly accessible database. Please amend the supplementary material to include the referenced data or remove the references.**

We appreciate the Editor’s comment regarding the use of the phrase “data not shown.” In accordance with the PLOS data access policy, we have removed all such references from the manuscript. The analyses previously referred to in this manner are now fully presented in the revised version of the manuscript and its supplementary materials.
To ensure transparency and completeness, we substantially expanded the supporting material and incorporated the corresponding data, figures, and analytical outputs. Specifically, the initial submission included 4 main figures, 3 tables, 2 supplementary figures, and 4 supplementary tables, whereas the revised manuscript now contains 5 main figures, 3 tables, 7 supplementary figures, 7 supplementary tables, and 3 additional supplementary files. These additions provide the underlying results and methodological details that were previously summarized, including expanded model performance analyses and machine-learning evaluation metrics.
We believe these changes fully address the journal’s data availability requirements and improve the transparency and reproducibility of the study.

3. **Tables should not be uploaded as individual files. Please remove these files and include the Tables in your manuscript file as editable, cell-based objects. For more information about how to format tables, see our guidelines: https://journals.plos.org/plosntds/s/tables**
   We included Tables as indicated.

4. **We notice that your supplementary figures are uploaded with the file type 'Figure'. Please amend the file type to 'Supporting Information'. Please ensure that each Supporting Information file has a legend listed in the manuscript after the references list.**
   We have uploaded the supplementary figures as indicated.

5. **Some material included in your submission may be copyrighted. According to PLOSu2019s copyright policy, authors who use figures or other material (e.g., graphics, clipart, maps) from another author or copyright holder must demonstrate or obtain permission to publish this material under the Creative Commons Attribution 4.0 International (CC BY 4.0) License used by PLOS journals. Please closely review the details of PLOSu2019s copyright requirements here: PLOS Licenses and Copyright. If you need to request permissions from a copyright holder, you may use PLOS's Copyright Content Permission form.**

Please respond directly to this email and provide any known details concerning your material's license terms and permissions required for reuse, even if you have not yet obtained copyright permissions or are unsure of your material's copyright compatibility. Once you have responded and addressed all other outstanding technical requirements, you may resubmit your manuscript within Editorial Manager.

Potential Copyright Issues:
- Figure 4. Please confirm whether you drew the images / clip-art within the figure panels by hand. If you did not draw the images, please provide (a) a link to the source of the images or icons and their license / terms of use; or (b) written permission from the copyright holder to publish the images or icons under our CC BY 4.0 license. Alternatively, you may replace the images with open source alternatives. See these open source resources you may use to replace images / clip-art:
- https://commons.wikimedia.org
- https://openclipart.org/.

We have now updated Figure 5 (previous Fig. 4) using icons from *Mind The Graph* software (https://mindthegraph.com/) paid subscription (fg0.lgcm@gmail.com; invoice number: 7850). Paid subscribers are recognized as authors and are entitled to full rights to their creations, with the rights and ability to transfer authorial rights to third-party publishers without previous consulting *Mind The Graph*.
"Fig. 5. Pathways and Gene Networks"

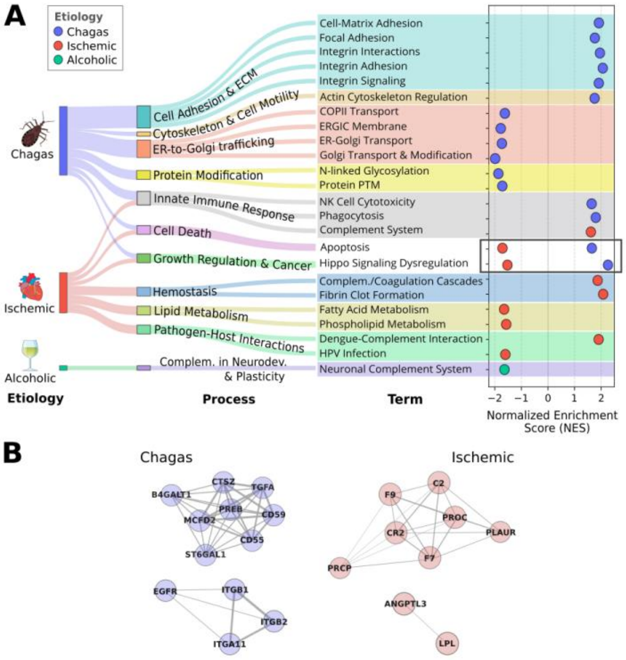

6. **In the online submission form, you indicated that "The data that support the findings of this study are available from the corresponding author upon reasonable request.". All**

PLOS journals now require all data underlying the findings described in their manuscript to be freely available to other researchers, either

1. In a public repository

2. Within the manuscript itself

3. Uploaded as supplementary information.

This policy applies to all data except where public deposition would breach compliance with the protocol approved by your research ethics board. If your data cannot be made publicly available for ethical or legal reasons (e.g., public availability would compromise patient privacy), please explain your reasons by return email and your exemption request will be escalated to the editor for approval. Your exemption request will be handled independently and will not hold up the peer review process, but will need to be resolved should your manuscript be accepted for publication. One of the Editorial team will then be in touch if there are any issues.

As indicated, the data are now available in the manuscript and the supplementary information.

Only the participant-level datasets cannot be made publicly available for legal and ethical reasons, in compliance with the protocol approved at the local IRB, as public availability could compromise patient privacy. We have now updated the *Data Availability Statement* to reflect this:

"Data Availability Statement:

Due to legal and ethical restrictions related to participant privacy, the participant-level datasets generated and analyzed during the current study are not publicly available and cannot be shared."

7. **Please amend your detailed Financial Disclosure statement. This is published with the article. It must therefore be completed in full sentences and contain the exact wording you wish to be published.**

- State the initials, alongside each funding source, of each author to receive each grant. For example: "This work was supported by the National Institutes of Health (####### to AM; ###### to CJ) and the National Science Foundation (###### to AM)."

- State what role the funders took in the study. If the funders had no role in your study, please state: "The funders had no role in study design, data collection and analysis, decision to publish, or preparation of the manuscript.".

If you did not receive any funding for this study, please simply state: "The authors received no specific funding for this work"

We have now updated the Funding statement as follows:

Funding: This work was supported by Fundação de Amparo à Pesquisa do Estado de São Paulo [INCT/FAPESP - 2014/50889-7, 2013/17368-0 and 2015/50216-5 to JEK], the Conselho Nacional

de Desenvolvimento Científico e Tecnológico – CNPq [INCT - 408940/2024-30, 309179/2013-0 and 442643/2020-9 to JEK], the Zerbini Foundation and Foxconn Brazil (Omics in Cardiovascular Medicine Grant to JEK). The funders had no role in study design, data collection and analysis, decision to publish, or preparation of the manuscript.

__Reviewer #1:__

Essential analyses / items to include in revision

1. **Table with N and number of deaths per etiology and by LVEF strata**
   We have now updated Table 1 as indicated.

2. **Add calibration plots and decision curve analysis (P9 vs NT-proBNP vs MAGGIC) in the test set, and present iAUC with 95% CI.**

We have now added a new supplementary figure with calibration, decision curve analysis and net benefit plots (Suppl. Fig. S5), and updated the Methods, Results and Discussion accordingly.

"Suppl. Fig. S5. Clinical utility of P9 vs NT-proBNP"

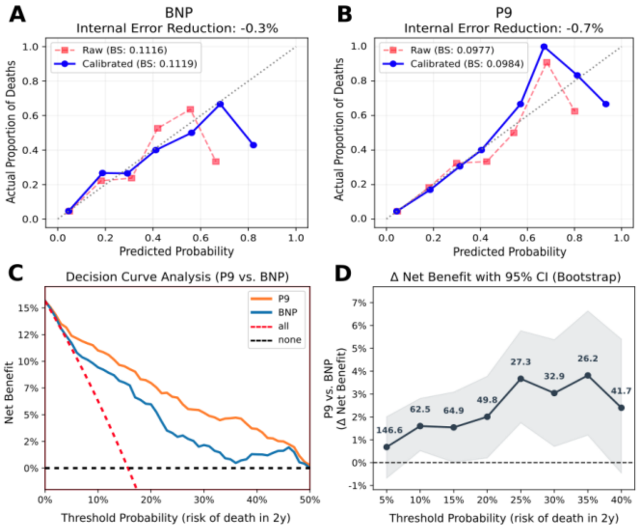

Legend: Clinical utility of P9 vs. BNP for 2-year mortality. (A) Calibration plot showing predicted versus observed mortality for the BNP model. The dotted diagonal line represents perfect calibration. The dotted red curve with squares shows raw predictions, while the solid blue curve with circles shows calibrated probabilities. Brier scores are also shown. (B) P9 Calibration plot, following the same strategy. (C) Decision Curve Analysis (DCA) comparing the P9 (orange) and BNP (blue) models. The "Treat All" strategy (red dotted line) starts and ends at the outcome prevalence (~16%), while the "Treat None" strategy (black line) remains at zero. A model is clinically useful when its curve sits above both baselines. (D) Δ Net Benefit across threshold probabilities: 95% CIs (shaded area) indicate significant superiority for P9 where the intervals exclude zero (based on 1,000 bootstraps). Numeric labels indicate the net reduction in unnecessary interventions per 100 patients achieved by using P9 instead of BNP without

missing true cases. For example, at a 5% threshold, P9's efficiency gain means avoiding 146.6 unnecessary interventions per 100 patients compared to BNP while maintaining equal safety.”

- section “Material and Methods”:

Calibration and Decision Curve Analysis

We employed *CalibratedClassifierCV* function from scikit-learn for calibration analysis. The training dataset was partitioned into a 5-fold cross-validation (CV) scheme. During the CV process, the model was iteratively trained on four folds and used to generate predicted values (scores) for the remaining held-out fold. Upon completion of all folds, these out-of-sample predictions were aggregated into a single set of calibrated values for all training samples. Subsequently, thresholding was applied to this combined, calibrated model to optimize the decision boundary for classification. Finally, the calibrated model with the optimized classification threshold was applied to the unseen test set. The predictive performance was evaluated by plotting test-set predicted values against their ground truth values, from which the final error metrics and the percentage of error reduction were derived (using Brier scores).

We further implemented Decision Curve Analysis (DCA), using the *dcurves* v1.1.7 library (34) to evaluate the clinical utility of two predictive models (BNP and P9) for 2-year mortality. Predicted probabilities of death were generated for the test cohort and used to compute net benefit across threshold probabilities ranging from 0 to 50%. Net benefit was calculated as the proportion of true positives adjusted for false positives weighted by the odds of the threshold probability. DCA curves were plotted for each model alongside “treat all patients” and “treat none” strategies.

- section “Results”:

Calibration procedures did not improve model performance relative to the raw models. The raw P9 model showed the lowest Brier score (0.0977), slightly better than the calibrated P9 (0.0984) and both NT-proBNP versions (raw: 0.1116; calibrated: 0.1119) (Suppl. Fig. S5A–B). In Decision Curve Analysis, the P9 model consistently provided greater net benefit than NT-proBNP and the default strategies of treating all or no patients (Suppl. Fig. S5C). Although NT-proBNP showed modest benefit over the “treat all” strategy at lower thresholds, it remained inferior to P9 across most clinically relevant ranges.

- section “Discussion”:

P9 performance in overall HF: The improved predictive performance of the P9 panel over NT-proBNP across most HF etiologies prompted further evaluation of model reliability and clinical utility through calibration and decision-analytic performance.

Regarding calibration, the lower classification error observed with the raw NT-proBNP and P9 models compared with calibrated versions likely reflects the machine-learning framework used. Logistic regression employs log-odds through the canonical logit link and is trained by minimizing log-loss (cross-entropy), a proper scoring rule that promotes well-calibrated probability estimates. Empirical studies support this property: Niculescu-Mizil and Caruana (46) showed that logistic regression models are typically well calibrated without post-hoc adjustment, while Gutman et al. (47) demonstrated that optimization of log-loss under the

canonical link confers the balance property, making well-specified models intrinsically calibrated. Consistent with this principle, raw models performed as well as, or slightly better than, isotonic-calibrated counterparts based on Brier score, suggesting that additional recalibration may introduce variance without improving probabilistic accuracy.

Decision Curve Analysis (DCA) showed that P9 consistently outperformed NT-proBNP across clinically relevant threshold probabilities. Within the 10–35% range, where P9 yielded positive Δ net benefit, the number of additional patients needing evaluation with P9 rather than NT-proBNP to correctly identify one additional death within two years ranged from 63 (10% threshold) to 26 (35% threshold). These findings indicate that incorporating P9 improves risk stratification and may support more individualized clinical decision-making in HFrEF.

Importantly, the superiority of P9 over NT-proBNP was also observed in the independent UKBB validation dataset, supporting robustness and generalizability. Although overall performance metrics were lower in UKBB, consistent with its community-based design and lower disease severity, P9 still achieved higher F1-macro scores, sensitivity, and positive predictive value than NT-proBNP alone. These findings support the clinical relevance of the P9 panel for risk stratification across diverse populations and may contribute to more efficient allocation of healthcare resources, particularly in universal health systems in low- and middle-income countries such as Brazil.

We have also incorporated confidence intervals to the iAUC curves in Figure 2C:
“Fig. 2. Comparative classification performance of the P9 panel and NT-proBNP”

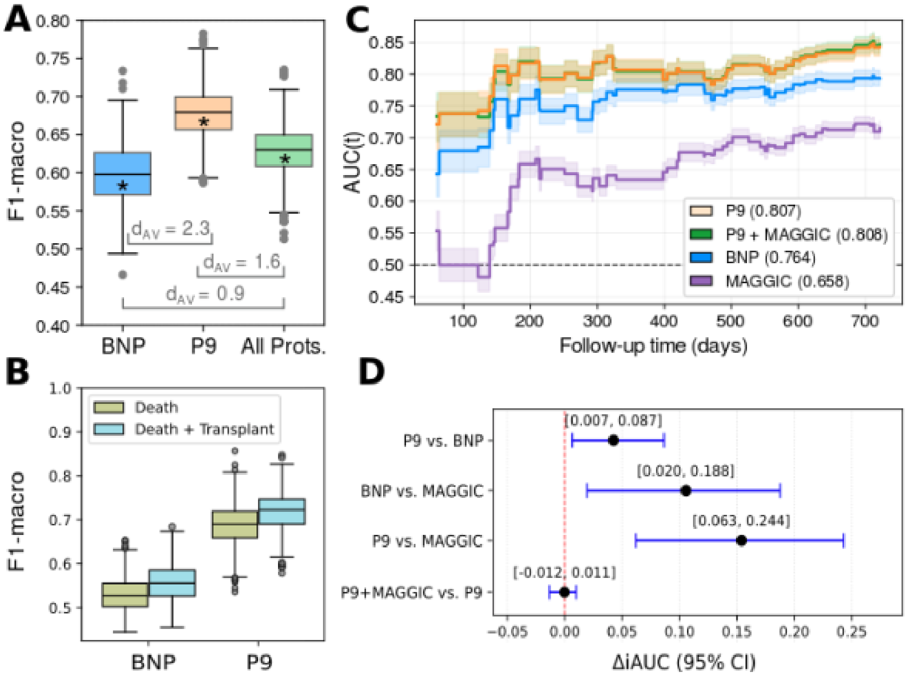

Legend: Distribution of predictive performance across biomarker models. (A) Boxplots show the F1-macro scores obtained from 1,000 random train-validation permutations (70/30 split, employing only the original train set) using logistic regression classifiers built with three feature sets: NT-proBNP ("BNP"), the 9-protein panel ("P9"), and the full proteomic panel with 734 proteins ("All Prots."). All effect size (Cohen's dAV) comparisons were of large magnitude (≥ 0.8) (B) Predictive performance for the endpoint of all-cause mortality ("Death") and for the composite outcome of death or heart transplantation between marker sets. Boxplots depict the distribution of F1-macro scores across 1,000 test set bootstraps using logistic regression models built with NT-proBNP (BNP) and the 9-protein panel (P9). A two one-sided tests (TOST) procedure was applied to formally assess equivalence between models for the same outcome (distributions were not significantly equivalent). (C) Time-dependent discrimination of biomarker models P9, P9 + MAGGIC, BNP, and MAGGIC for right-censored Cox proportional hazards survival prediction in the test set, under a cumulative/dynamic framework. Stepwise curves show AUC(t) with 95% highest density intervals (HDI) derived from 1,000 bootstraps, restricted to events occurring ≥ 50 days. (D) Forest plot comparing iAUC between models in the test set. Horizontal bars denote 95% highest density intervals (HDI), deemed significant in case they exclude zero (red dotted line)

"Suppl. Fig. S7. Impact of estimated glomerular filtration rate in the P9 model."

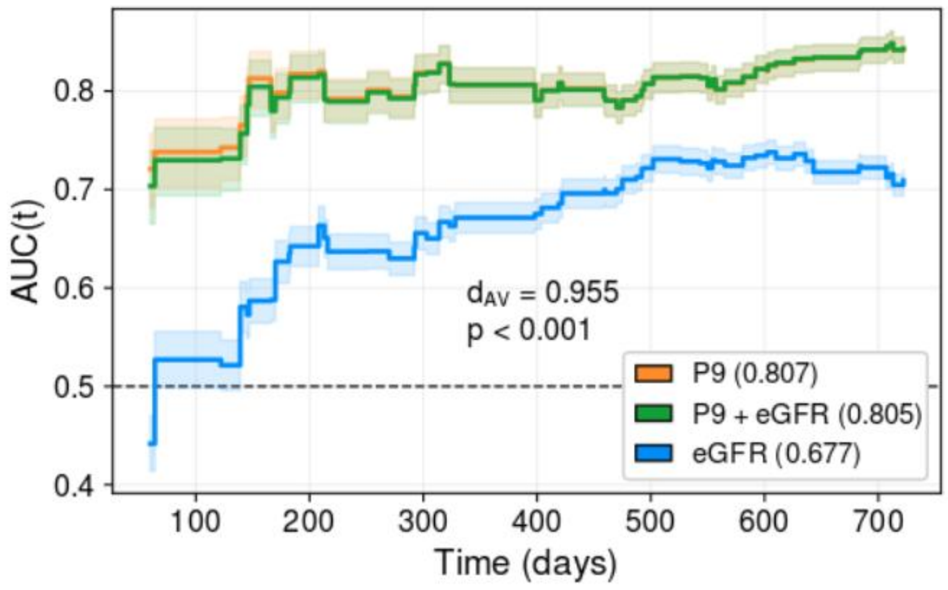

Legend: Effect of estimated glomerular filtration rate (eGFR) model compared to P9 model, with asymptotic influence-function variance and logit-transformed 95% confidence intervals

(CI). Cohens' dAV was calculated between P9 vs. eGFR, as the compound model "P9 + eGFR" bears no gains compared to P9 alone.

3. **Present event counts and EPV and perform optimism correction (bootstrap) or nested CV to estimate unbiased performance**

We have now updated the text to include EPV. We did not include optimism correction, as we have now provided external validation of the model.

Methods section, *Events Per Variable* subsection

"The EPV (Events Per Variable), here estimated as the number of deaths in the training set (where all analyses and models were estimated) divided by the number of predictor variables (i.e., independent variables and covariates), was checked for NT-proBNP (BNP hereafter) and P9 models, to check for statistical power of the model."

Results section

"We obtained the EPV for NT-proBNP and P9 models. The NT-proBNP model yielded EPV=47.25, while P9 yielded EPV=15.75, all above the threshold of 10 subjects per variable. This indicates the models are not impacted by lack of power."

4. **Provide stability analysis for feature selection (frequency of inclusion across resamples).**

We agree that evaluating feature selection stability, such as the frequency of variable inclusion across resampling iterations, can be informative when the primary objective is to identify reproducible biological markers. However, the primary aim of our study was to develop a parsimonious prognostic model optimized for predictive performance, rather than to define a stable set of mechanistically definitive biomarkers.

In predictive modeling, particularly in the presence of correlated predictors, multiple combinations of variables can yield comparable predictive performance. Consequently, the specific set of selected features may vary across resamples without materially affecting key model properties such as discrimination, calibration, or overall predictive accuracy. For this reason, the selected features should be interpreted as one effective solution within a broader set of predictors capable of achieving similar prognostic performance, rather than as uniquely stable biological determinants.

5. **Explicit description of BNP/NT-proBNP assay and Olink assay handling (batch, QC).**

We thank the reviewer for this suggestion and have expanded the Proteomic Panel Quantification subsection in the Methods to provide a clearer description of the Olink assay procedures, sample handling, and quality-control procedures. We also incorporated additional references and clarified that NT-proBNP measurements used in the analyses were obtained from the Olink Cardiometabolic panel, rather than from a separate clinical assay.

Briefly, baseline plasma samples were collected in EDTA tubes, processed under standardized conditions, aliquoted to prevent repeated freeze–thaw cycles, stored at −80 °C,

and shipped on dry ice for analysis. Proteomic profiling was performed using the Olink Explore 384 Cardiometabolic and Inflammation panels, which together quantify 734 proteins using the Proximity Extension Assay (PEA) coupled with next-generation sequencing readout. Protein levels were reported as normalized protein expression (NPX) values on a log2 scale following standard Olink normalization procedures.

We also added detailed quality-control metrics. QC procedures included thresholds for matched counts per sample, deviation limits for incubation and amplification controls, and assay-specific limits for negative controls. All samples were processed within a single Olink project run, minimizing potential batch effects; therefore, no additional post-hoc batch correction beyond standard NPX normalization was required.

Finally, we now report the QC performance metrics in the Results section, where 98% of datapoints passed QC for the Cardiometabolic panel and 97% for the Inflammation panel, with an average intra-assay coefficient of variation of 9% and inter-assay coefficient of variation of 17%. Detailed information on all quantified proteins is provided in Supplementary Table S1.

6. **Provide full details of PyMC model and genetic algorithm (priors, iterations, parameters), plus code availability (GitHub link) and TRIPOD checklist.**

We thank the reviewer for this important suggestion. In response, we have expanded the methodological description of the feature selection framework and provided the full computational documentation required for reproducibility.

First, we added a new supplementary figure (Suppl. Fig. S4) describing the complete proteomic feature selection workflow, including the Genetic Algorithm (GA) and the Bayesian Stochastic Search Variable Selection (SSVS) implemented in PyMC. The Methods section has also been updated to provide additional details on model configuration, parameter settings, and the sequential selection procedure.

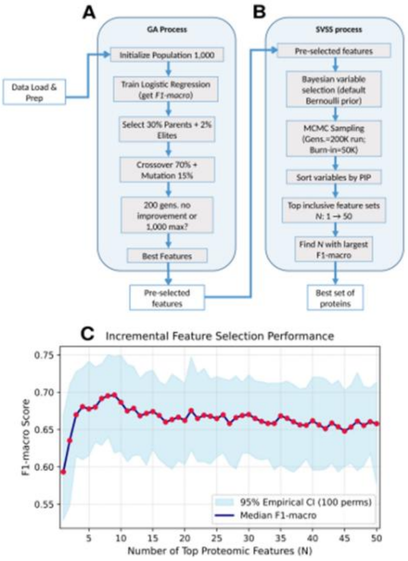

Briefly, the GA stage used logistic regression models optimized to maximize the F1-macro score across the 734 quantified proteins. The evolutionary strategy included 30% parent selection, 2% elite retention, 70% crossover probability, and 15% mutation rate, and was run for up to 1,000 generations, with early stopping triggered after 200 generations without improvement. This stage yielded 322 candidate features. These were subsequently refined through Bayesian variable selection using SSVS implemented in PyMC, with a Bernoulli prior on inclusion indicators and posterior sampling performed via 200,000 MCMC iterations with a 50,000-iteration burn-in to ensure chain stabilization. Variables were then ranked according to posterior inclusion probability (PIP).

To ensure full transparency and reproducibility, we have also provided the complete code used for feature selection and model development as supplementary material (Suppl. Files F2 and F3), and included the TRIPOD checklist and reporting documentation (Suppl. File F1). These additions provide full details of the modeling framework, parameterization, and computational workflow used in the study.

7. **Tone down drug repurposing claims, add explicity safety caveats and tempered language for repurposing suggestions (no clinical recommendations without preclinical/controlled validation). Also add explicit safety caveats regarding immunosuppression in CCC.**

We have revised the Results and Discussion sections to moderate the language regarding drug repurposing and to clearly emphasize the exploratory nature of these findings. The analyses identifying potential therapeutic targets are now explicitly framed as hypothesis-generating, rather than as clinical recommendations.

Specifically, the Results section has been revised to present the identified compounds only as potential pathway-linked candidates emerging from the druggability analysis. Correspondingly, the Discussion now explicitly states that any therapeutic implications remain speculative and require rigorous preclinical validation before consideration in clinical studies.

We also added explicit safety caveats, particularly regarding the potential risks of immunosuppression in patients with chronic Chagas cardiomyopathy, where immunomodulatory therapies may increase the risk of *Trypanosoma cruzi* reactivation. In addition, we note that some candidate compounds identified through pathway analysis (e.g., anti-tumoral agents) carry known cardiotoxicity risks, further underscoring that off-label clinical use cannot be recommended without appropriate experimental and controlled clinical validation.

These revisions aim to ensure that the interpretation of the druggability analysis remains cautious, biologically informative, and consistent with the standards for translational inference in observational omics studies.

Major concerns/actions needed:

8. **External validation and overfitting risk: conclusions rely on internal train/test split only. With high-dimensional proteomics there is substantial overfitting risk. CCC subgroup is moderate in size and may be underpowered for complex models. If an independent cohort exists, validate P9 and the CCC finding there and report iAUC, calibration, and reclassification. If no cohort is available, at minimum perform stronger internal validation: repeated/nested cross-validation or bootstrap optimism correction for iAUC and report optimism-corrected estimates with 95% CI. Temper claims about generalizability in the manuscript until external validation is done.**

To address this point, we conducted an external validation of the P9 model using data from the UK Biobank, a large independent population-based cohort with available Olink proteomic measurements. We have now expanded the manuscript to include these analyses and added a new main figure (Fig. 4) as well as two supplementary tables (Suppl. Tables S4 and S5) presenting the full validation results.

The Methods section now includes a dedicated **External Validation** subsection describing the UK Biobank cohort, the inclusion criteria for heart failure cases with proteomic data, and the analytical strategy used to evaluate model performance. Because the UK Biobank cohort represents a community-based population with generally lower disease severity than the GENIUS-HF cohort, we performed additional analyses restricted to individuals in the highest MAGGIC risk tertile to better approximate the clinical risk distribution observed in the derivation cohort.

The Results section has been expanded accordingly to report the external validation findings, including F1-macro, time-dependent discrimination (iAUC), sensitivity, specificity, positive predictive value, and net reclassification improvement (NRI). These analyses show that the P9 model improves classification performance compared with BNP in the independent cohort, particularly in identifying patients who died during follow-up.

We note, however, that external validation of the CCC-specific findings was not feasible, as to our knowledge no independent cohorts currently exist with both proteomic profiling and well-characterized chronic Chagas cardiomyopathy patients. We have clarified this limitation in the manuscript.

9. **Naive Bayes was chosen, but NB assumes conditional independence of predictors — unlikely with correlated proteins. The PyMC to genetic algorithm pipeline is not sufficiently described (priors, hyperparameters, convergence, selection stability). Please provide a clear pipeline diagram; report comparative performance (iAUC, calibration, Brier score) of other sensible methods (penalized Cox/logistic, XGBoost, random forest) in**

the test set. Give full PyMC model details (priors, iterations, diagnostics) and genetic algorithm parameters. Report selection stability (frequency each protein appears across resamples/bootstraps). If NB remains best, explain why and discuss limitations of the independence assumption.

We thank the reviewer for this comment regarding model choice and methodological transparency. In response, we have revised the analysis pipeline and expanded the methodological description.

First, we replaced the Naïve Bayes classifier with logistic regression, which is more appropriate for correlated predictors and avoids the conditional independence assumption inherent to Naïve Bayes. The results obtained with logistic regression were consistent with the original findings, confirming the robustness of the predictive signal.

Second, we expanded the evaluation of alternative modeling approaches. Comparative performance across multiple algorithms, including logistic regression, penalized regression, random forest, gradient boosting, and other machine-learning classifiers, is now reported in Supplementary Fig. S3, which presents the distribution of F1-macro scores across 1,000 resampling iterations. Logistic regression demonstrated competitive performance while maintaining interpretability and stability, supporting its selection as the primary model.

Third, we have substantially expanded the description of the feature selection pipeline, including the genetic algorithm and Bayesian stochastic search variable selection implemented in PyMC, and added a workflow diagram (Suppl. Fig. S4) to clarify the sequential steps of the procedure. Detailed parameter settings, model configuration, and code availability are provided as described in our response to comment #6, along with the TRIPOD checklist and reproducible code (Suppl. Files F1–F3).

Together, these revisions improve the transparency of the modeling strategy and address the reviewer's concerns regarding algorithm choice, methodological description, and reproducibility.

10. iAUC differences are reported as percent gains but without confidence intervals and clear statement whether gains are absolute points or relative percent. TOST equivalence margin needs justification. Methods for handling censoring in time-dependent ROC are not fully described. Please report absolute iAUC values with 95% CI and exact p-values; show time-dependent AUCs at 6/12/24 months. State the statistical method used for time-dependent AUC and how censoring was handled. Provide exact TOST results and justify the chosen equivalence margin (0.05 absolute iAUC).

We thank the reviewer for highlighting the need for greater clarity in the reporting of discrimination metrics and time-to-event methodology. In response, we have revised the manuscript to improve the presentation and statistical transparency of these analyses.

First, absolute iAUC values with corresponding 95% confidence intervals are now reported in the revised manuscript, rather than only relative percentage changes. The Results section and figure legends were updated accordingly to clearly distinguish absolute differences in iAUC from relative percentage improvements.

Second, we have added a new figure (Suppl. Fig S6) presenting time-dependent cumulative/dynamic ROC curves with AUC(t) values evaluated at 6 months, 12 months, and 24 months, allowing direct visualization of model discrimination across clinically relevant follow-up intervals.

Third, the Methods section has been expanded to explicitly describe the statistical approach used to estimate time-dependent AUC. Specifically, discrimination was assessed using the inverse probability of censoring weighting (IPCW) estimator proposed by Uno et al., which accounts for right-censoring under a cumulative/dynamic ROC framework in which cases are defined as events occurring up to time *t* and controls are individuals remaining at risk beyond that time point.

Fourth, the Results section now clarifies the relationship between AUC(t) and integrated AUC (iAUC). While AUC(t) measures discrimination at a specific follow-up time, iAUC represents a weighted average of discrimination across the entire follow-up interval, which explains minor differences between these metrics when evaluated at the 2-year horizon.

Finally, the equivalence testing results are now reported explicitly, including the TOST procedure parameters and statistical outputs, and the rationale for the pre-specified equivalence margin (0.05 absolute iAUC) has been clarified in the Methods section as representing a conservative threshold for clinically meaningful discrimination differences in prognostic models.

Together, these revisions provide a clearer statistical description of model discrimination, improve transparency in the reporting of uncertainty estimates, and allow readers to evaluate predictive performance across multiple clinically relevant time points.

11. Only discrimination metrics are shown. For clinical translation we need calibration and net benefit. The study will benefit by adding calibration plots and metrics (calibration-in-the-large, calibration slope) for P9 and BNP in the test set, overall and by etiology (esp. CCC). Please add decision-curve analysis to show net benefit across clinically relevant thresholds. Consider reporting NRI or clinical impact measures.

We thank the reviewer for this suggestion regarding the assessment of clinical utility. In response, we expanded the analyses beyond discrimination metrics and incorporated calibration and decision-analytic evaluations in the revised manuscript.

Specifically, we added calibration plots and Brier score comparisons for the P9 and NT-proBNP models in the test set, which are now presented in Supplementary Fig. S5. These analyses evaluate the agreement between predicted and observed risks and demonstrate that

the raw models were already well calibrated, with no improvement obtained through post-hoc recalibration.

In addition, we implemented Decision Curve Analysis (DCA) to assess the clinical net benefit of the models across a range of clinically relevant threshold probabilities. The results, also shown in Supplementary Fig. S5, indicate that the P9 model consistently provides higher net benefit than the NT-proBNP model across the 10–35% threshold range, supporting its potential clinical utility for mortality risk stratification.

We also expanded the reporting of reclassification metrics, including net reclassification improvement (NRI) and related classification measures (sensitivity, specificity, positive predictive value), which are now presented in the Results and in the external validation analyses.

These additions substantially strengthen the evaluation of model performance by addressing calibration, discrimination, and clinical decision utility, thereby improving the translational interpretation of the predictive models.

12. Samples with <99% protein data were excluded; that's strict and may bias results. Median imputation for missing proteomic values is simplistic and could be inappropriate if missingness is not random. The number and characteristics of excluded samples are not reported. Please report the N excluded and their characteristics. Compare included vs excluded. Use multiple imputation for clinical covariates and consider principled imputation approaches for proteomic data (or at least sensitivity analyses comparing median imputation with other approaches). Discuss missingness assumptions.

We thank the reviewer for raising these points regarding missing data handling and potential bias. In response, we have clarified the assumptions regarding missingness and expanded the description of preprocessing procedures in the Methods (Pre-processing subsection).

First, we note that the overall proportion of missing values in the dataset prior to imputation was low (~2%) and sparsely distributed, without evidence of systematic clustering of missingness across samples or variables. Under these conditions, median imputation was selected as a simple and robust approach, particularly given the skewed distributions and presence of extreme values in several clinical predictors, for which median-based imputation is less sensitive to outliers than mean-based methods.

Second, we conducted sensitivity analyses using an alternative imputation strategy (k-nearest neighbors, KNN) for the proteomic data. The predictive performance of the resulting models was essentially unchanged, with F1-macro values differing only at the fourth decimal place, indicating that the results were not sensitive to the choice of imputation method.

Third, because the fraction of missing data was very small (~2%) and exclusions due to incomplete proteomic data were minimal, comparisons between included and excluded samples were not expected to meaningfully affect the analysis. We have clarified this point and explicitly stated the missingness assumptions in the Methods section.

These revisions improve transparency regarding the handling of missing data and demonstrate that the study results are robust to alternative imputation approaches.

13. The manuscript uses BNP and NT-proBNP terms inconsistently. Since part of the mechanistic argument rests on peptide processing/glycosylation, assay details matter. Choose consistent nomenclature, and clearly describe the clinical natriuretic assay used (BNP or NT-proBNP: assay manufacturer, LOD, units). If both measurements or Olink NPX for that analyte were used, clarify how they relate.

We thank the reviewer for highlighting the need for consistent terminology and clearer assay description. In response, we have revised the manuscript to ensure consistent use of the term NT-proBNP throughout the text, removing any ambiguity between BNP and NT-proBNP.

We also clarified in the Methods (Proteomic Panel Quantification subsection) that the natriuretic peptide measurements used in the analyses were obtained from the Olink Explore Cardiometabolic panel, and therefore represent NT-proBNP quantified using the Olink proximity extension assay (PEA) platform. Protein levels were reported as normalized protein expression (NPX) values on a log2 scale, which are relative quantification units derived from the next-generation sequencing–based readout of the Olink assay.

No separate clinical immunoassay measurements of BNP or NT-proBNP were used in the study. We have updated the Methods section to clearly state this and to explain the relationship between the measured NT-proBNP NPX values and the biomarker used in the predictive models. These revisions ensure consistent nomenclature and clarify the analytical context of the NT-proBNP measurements used in the study.

14. Mapping from measured proteins to gene sets and to drugs is nontrivial. Also recommending prednisone or EGFR monoclonals for Chagas patients without strong caveats is risky: systemic immunosuppression can reactivate T. cruzi and EGFR inhibitors have cardiovascular toxicities. Include method details for protein to gene mapping, report number of proteins measured per pathway and full GSEA tables in supplement. Reframe druggability findings as hypothesis-generating and add explicit safety cautions (parasitic reactivation with immunosuppression, known cardiac toxicities of anticancer agents). Tone down direct therapeutic recommendations throughout the manuscript.

We thank the reviewer for raising these points regarding pathway mapping, interpretation of druggability analyses, and safety considerations. In response, we implemented several

revisions to improve methodological transparency and to ensure that therapeutic interpretations are appropriately cautious.

First, we expanded the reporting of the pathway enrichment analyses and now provide the complete results in Supplementary Table S6, which includes the enriched pathways, normalized enrichment scores (NES), false discovery rate (FDR q-values), gene coverage statistics, and the leading genes contributing to each signal. This table also reports the mapping between the measured proteins and the corresponding gene sets used in the enrichment analyses, allowing readers to assess the number and identity of proteins contributing to each pathway.

Second, we revised the Results and Discussion sections to substantially tone down the interpretation of druggability findings. The identification of compounds potentially linked to enriched pathways is now explicitly framed as hypothesis-generating, rather than as therapeutic recommendations.

Third, we incorporated explicit safety caveats in the Discussion. In particular, we highlight that systemic immunosuppressive strategies could pose risks in chronic Chagas cardiomyopathy, including the potential for *Trypanosoma cruzi* reactivation, and therefore cannot be considered without rigorous experimental and clinical validation. Similarly, we emphasize that EGFR-targeting monoclonal antibodies and other anticancer agents identified through pathway mapping are associated with known cardiovascular toxicities, further reinforcing that these findings should be interpreted strictly as exploratory biological signals rather than actionable treatment strategies.

Finally, we revised the language throughout the manuscript to ensure that the druggability analysis is presented as a systems-biology approach to identify pathways potentially amenable to future investigation, rather than as a basis for clinical repurposing recommendations. These revisions aim to maintain the biological insights provided by the pathway analysis while aligning the interpretation with appropriate translational caution.

15. The number of deaths per etiology is not clearly shown in the main text. Multi-protein models in small subgroups risk low events-per-variable. Include a table listing N and deaths per etiology and per LVEF stratum. Calculate events-per-variable for P9 and other models. Also discuss limitations where EPV is low and consider simpler models in small subgroups.

We thank the reviewer for highlighting the importance of clearly reporting event counts and considering events-per-variable (EPV) when evaluating multi-predictor models.

In response, we have updated Table 1 to explicitly report the number of patients and deaths by heart failure etiology, allowing readers to assess the distribution of events across clinically relevant subgroups. These additions improve the transparency of the cohort characteristics and address the concern regarding event counts in smaller etiological groups.

We also incorporated EPV calculations for the P9 model and related analyses in the main text, clarifying the relationship between the number of predictors and the number of observed events. Because the P9 panel contains nine predictors, the EPV remained within an acceptable range for the overall cohort used for model development and validation.

Finally, we expanded the Discussion section to acknowledge the limitations associated with subgroup analyses where the number of events is smaller, noting that predictive estimates in these strata should be interpreted with caution. We also clarify that the primary conclusions of the study are based on the overall cohort analyses, where the EPV is adequate and the model estimates are more robust.

These revisions improve transparency regarding event distribution and modeling constraints, while appropriately contextualizing the interpretation of subgroup analyses.

16. Concerns about potential confounding such as socioeconomic status, treatment differences, antiparasitic therapy, etc... CCC patients have different socioeconomic characteristics, less comorbidity, and possibly different treatments. Antiparasitic therapy (e.g, benznidazole) history is not reported. These factors could confound proteomic signals or outcomes. Please report antiparasitic treatment rates, device therapy (ICD/CRT), medication doses/adherence if available. In addition, include sensitivity analyses adjusting for socioeconomic markers and treatment differences.

We thank the reviewer for raising these concerns regarding potential confounding factors, including socioeconomic status, treatment differences, and antiparasitic therapy. We carefully reviewed the available data to address these points.

First, information on prior antiparasitic treatment (e.g., benznidazole) was not reliably available in our cohort. Most CCC patients were referred to our tertiary cardiology center many years after the acute infection phase, and historical treatment data were not systematically documented in the clinical records. For this reason, we were unable to include antiparasitic therapy as a covariate in the analyses, and we have now clarified this limitation in the manuscript.

Second, detailed data on device therapy (ICD or CRT) were not systematically collected at baseline in the dataset used for the present analysis. However, because patients were referred to the tertiary center primarily for diagnostic evaluation and specialized care, it is unlikely that a substantial proportion had device therapy already implanted at the time of enrollment. We have clarified this point in the limitations.

Third, although medication classes were available, reliable data on medication doses and adherence were not consistently documented, preventing robust adjustment for these variables. Importantly, pharmacologic treatment patterns did not differ substantially between the CCC and non-CCC heart failure groups, reducing the likelihood that treatment differences materially confounded the observed proteomic associations.

To further address potential socioeconomic confounding, we conducted additional sensitivity analyses adjusting for standardized income, which was included as an additional covariate in the predictive model. As described in the revised Methods (Feature Selection subsection) and Results, inclusion of standardized income resulted in only modest changes in predictive performance, with the P9 model F1-macro increasing from 0.678 to 0.688 (≈1.5%) in the GENIUS-HF cohort. Similar results were observed in the UK Biobank validation cohort, where the inclusion of income increased the F1-macro from 0.575 to 0.580 (≈0.9%).

Together, these analyses suggest that socioeconomic differences have only a limited impact on the predictive performance of the P9 model. Nonetheless, we have expanded the Discussion to acknowledge the potential influence of unmeasured treatment-related factors and historical antiparasitic therapy as limitations of the present study.

17. Please avoid any causal language. The manuscript sometimes implies causality from association (for instance: “trafficking defects attenuate proteomic signal”). Rewrite to emphasize associations and hypothesis generation; reserve causal claims for future mechanistic studies.

We thank the reviewer for this comment. In response, we carefully revised the manuscript, particularly the Discussion section, to avoid causal language and ensure that all interpretations are framed as associations or hypothesis-generating observations rather than mechanistic conclusions.

Specifically, statements that could be interpreted as implying causality (e.g., references suggesting that specific biological processes drive or attenuate proteomic signals) were rewritten to emphasize that the findings reflect statistical associations between proteomic signatures, pathways, and clinical outcomes. The revised text now explicitly states that these observations suggest potential biological mechanisms that warrant further investigation, but do not establish causality.

We also moderated the tone of several conclusions throughout the manuscript to clarify that the study provides systems-level insights that generate testable hypotheses, while mechanistic validation will require dedicated experimental and translational studies. These revisions ensure that the interpretation of the results remains appropriately cautious and aligned with the observational design of the study.

<u>Minor points, proofreading and presentation:</u>

18. Define NPX on first use.

We have defined it accordingly in the Methods.

19. Reconcile LVEF definitions with current nomenclature (HFrEF typically ≤40%; HFmrEF 41–49%). If you choose <50% to define inclusion, justify it up front and consider standard strata in analyses.

We thank the reviewer for pointing out the need to clarify the LVEF definitions in relation to current heart failure nomenclature. Patient inclusion in this study began in 2012, before the formal recognition of heart failure with mildly reduced ejection fraction (HFmrEF; EF 41–49%), which was introduced in the literature and guidelines in 2014 (PMID: 25210008). For this reason, the original study protocol defined the inclusion criterion as LVEF <50%, which was consistent with the clinical classification used at the time of cohort enrollment.

To address this point, we have clarified the rationale for this threshold in the manuscript and added a statement in the Limitations section acknowledging that the inclusion criterion predates the current HFrEF/HFmrEF distinction. This clarification ensures transparency regarding the historical context of cohort enrollment and aligns the interpretation of the results with contemporary heart failure classifications.

20. In the methods, give details on sample handling (collection, processing, storage, freeze-thaw cycles), batch randomization and batch-correction method for Olink NPX. Also, add software versions and random seeds for reproducibility.

We thank the reviewer for this suggestion and have expanded the Methods section to provide additional details on sample handling and computational reproducibility.

First, we added a more detailed description of sample collection, processing, storage conditions, and handling procedures, including the use of EDTA tubes, plasma separation by centrifugation, aliquoting to avoid repeated freeze–thaw cycles, storage at −80 °C, and shipment on dry ice for proteomic analysis. We also clarified that all samples were processed at a single center and analyzed within a single Olink project run, minimizing potential batch effects. Consequently, no additional post-hoc batch correction was applied beyond the standard Olink NPX normalization procedures.

Second, to improve reproducibility of the computational analyses, we updated the manuscript to include software versions whenever analytical libraries or tools are cited in the Methods section. These revisions ensure greater transparency regarding both biospecimen handling procedures and computational implementation.

21. Replace "shuffling's" with "shuffles." Fix spacing (e.g., "FDR-corrected"). Replace vague p-values like "p << 0.01" with exact values when possible.

We have revised the manuscript to correct the grammatical issues identified, including replacing "shuffling's" with "shuffles" and correcting spacing inconsistencies (e.g., "FDR-corrected"). In addition, vague p-value expressions such as "p << 0.01" have been replaced with exact p-values whenever possible, reported to three decimal places to improve precision and

consistency with standard reporting practices. These edits were applied throughout the manuscript.

For figures, add number-at-risk tables to KM plots, calibration plots, and decision-curve graphs. For performance figures include CIs and sample sizes used for each comparison.

To avoid making the multi-panel figures overly crowded, we chose not to embed number-at-risk tables directly within the Kaplan–Meier plots. Instead, we have provided the number of individuals at risk over time for each etiology in Supplementary Table S2, which reports the counts at predefined follow-up intervals (baseline, 6 months, 1 year, 1.5 years, and 2 years). This approach preserves figure readability while ensuring that the underlying risk-set information is fully available to readers.

In addition, we have updated the performance figures to include confidence intervals (CIs) for the relevant metrics, as described in our response to comment #2. Where applicable, the sample sizes used for each comparison are now explicitly reported in the figure legends and corresponding tables. These revisions improve the transparency of the performance analyses while maintaining visual clarity of the figures.

22. In S1–S4, ensure UniProt IDs, median NPX per group, and full GSEA tables (with # measured genes per pathway) are present. Include a table showing how often each P9 protein was selected across resamples.

We revised the supplementary files to improve the presentation of pathway analysis results and ensure reproducibility of the proteomic data.

Specifically, the pathway enrichment results are now reported in an updated Supplementary Table S6 (previously Suppl. Table S5), which presents the full set of GSEA outputs, including enrichment scores, false discovery rate (FDR) values, pathway annotations, and the leading genes contributing to each signal.

We also verified that Supplementary Table S1 includes the UniProt identifiers for all quantified proteins, ensuring traceability and reproducibility of the proteomic measurements. To preserve readability of the supplementary material, we did not duplicate UniProt identifiers across additional tables, as this would substantially increase table size and reduce clarity.

Regarding feature selection stability, we did not perform resampling-based stability analyses for the feature selection stage, as explained in our response to comment #4. The feature selection procedure was designed to identify a parsimonious predictive feature set optimized for model performance rather than to estimate the frequency of selection across resamples. This clarification has been incorporated into the response and the manuscript where relevant.

23. Finally, “Millipede analysis” needs a citation or brief explanation.

We have now added an appropriate citation and brief description of the Millipede approach in the Methods section to clarify the analytical framework used. This addition provides readers with a reference for the methodology and ensures that the analytical procedure is properly documented, as noted in our response to comment #6.

Reviewer #2

1. **Are the objectives of the study clearly articulated with a clear, testable hypothesis stated? Yes, the overarching objectives are clearly described: to evaluate whether a sparse proteomic panel can improve 2-year mortality prediction in HFrEF across etiologies, and to determine whether chronic Chagas cardiomyopathy (CCC) exhibits distinct prognostic or biological features. While the manuscript does not state a single explicit hypothesis sentence, the testable hypotheses are implicit and adequately operationalized (e.g., that proteomic signatures improve risk prediction beyond BNP, and that CCC displays etiology-specific pathways). Stating the hypotheses more explicitly would improve clarity.**

We thank the reviewer for this constructive suggestion. In response, we have revised the Introduction to more clearly articulate the study objectives and to explicitly state the underlying hypotheses. The aims of the study are now presented in a structured manner, and a concise hypothesis statement has been added to clarify the testable assumptions guiding the analyses.

Specifically, we now state that the study had three primary objectives: (i) to develop sparse multi-protein models optimized for predicting all-cause mortality in patients with HFrEF across etiologies, including CCC; (ii) to benchmark their prognostic performance against established biomarkers such as NT-proBNP and across different HF etiologies; and (iii) to identify molecular pathways specifically associated with CCC to provide insights into disease biology and potential therapeutic targets.

To further improve clarity, we added an explicit hypothesis statement indicating that multi-protein proteomic models would provide improved prognostic performance compared with existing single-biomarker approaches and that CCC would exhibit distinct biological pathway signatures relative to other HF etiologies. These revisions strengthen the conceptual framing of the study and make the testable hypotheses more explicit.

2. **Is the study design appropriate to address the stated objectives? Overall, yes. A large, prospective cohort (GENIUS-HF) with adjudicated HF etiology and longitudinal mortality follow-up is appropriate for prognostic modeling and proteomic comparison across etiologies. The use of Olink Explore panels is suitable for circulating biomarker discovery and risk prediction. However, for mechanistic interpretation—especially claims about "unique CCC biology"—the platform's limitations should be acknowledged. Because Olink measures secreted proteins and the study finds downregulation of ER-to-Golgi trafficking and N-glycosylation specifically in CCC, the method may introduce a structural bias in this etiology. This does not compromise the prognostic aim but affects mechanistic depth.**

We thank the reviewer for this thoughtful comment regarding the suitability of the study design and the interpretation of proteomic findings. We agree that while the prospective cohort design of GENIUS-HF and the use of Olink Explore proteomic panels are well suited for

prognostic modeling and biomarker discovery, the platform's characteristics impose limitations when drawing mechanistic conclusions about disease biology.

In response, we have expanded the Limitations section in the Discussion to more clearly acknowledge these methodological considerations. Specifically, we now emphasize that the Olink platform primarily measures circulating proteins enriched for secreted factors, which capture systemic biological signals but may not fully represent tissue-level remodeling processes or cellular heterogeneity, both of which are particularly relevant in chronic Chagas cardiomyopathy. As a result, while the platform is appropriate for identifying prognostic proteomic signatures, it may provide a partial view of underlying biological mechanisms.

The revised text in the Limitations section now states:
"Second, our reliance on plasma proteomics with panels enriched for secreted proteins captures circulating signatures but does not directly reflect tissue-level remodeling or cellular heterogeneity, both of which are critical in CCC."

These revisions clarify that the primary strength of the study lies in prognostic modeling, while the biological pathway findings should be interpreted as hypothesis-generating insights rather than definitive mechanistic conclusions.

3. **Is the population clearly described and appropriate for the hypothesis being tested? Yes. The cohort is well characterized, with detailed demographic, clinical, socioeconomic, and echocardiographic data. CCC diagnosis by dual T. cruzi serology is appropriate. Etiology definitions follow standard cardiology criteria. The population is appropriate for prognostic evaluation; however, it is restricted to a single center in Brazil, which may influence generalizability, particularly for CCC where regional variation in T. cruzi DTUs exists. This limitation should be noted more explicitly in the Methods or Limitations.**

We agree that although the cohort is well characterized and appropriate for the prognostic objectives of the study, its single-center design may limit broader generalization of the findings, particularly for chronic Chagas cardiomyopathy where regional variation in Trypanosoma cruzi discrete typing units (DTUs) may influence disease biology.

In response, we have expanded the Limitations section in the Discussion to explicitly acknowledge this point. The revised text now notes that the absence of independent CCC cohorts with comparable proteomic data limits the possibility of external validation and may constrain the generalizability of the CCC-specific findings.

The manuscript now states in the Limitations and Future Perspectives subsection:
"Moreover, the lack of available datasets on Chagas disease with proteomics preclude external validation and may limit the generalizability of our findings."

This addition clarifies the potential geographic and biological constraints on extrapolating the CCC-specific results while preserving the validity of the prognostic analyses within the study cohort.

4. **Is the sample size sufficient to ensure adequate power to address the hypothesis being tested?**

Yes for the primary prognostic objective. The full sample (n=1,212) provides adequate power for multivariable modeling, and the CCC subgroup (n=191; 26% 2-year mortality) is one of the largest published to date. However, power for pathway analyses in etiologies with fewer events (e.g., alcoholic HF) is limited, which the authors appropriately acknowledge. For CCC, the sample size is sufficient for prognostic modeling but places limits on deeper mechanistic inference—another reason to present findings as hypothesis-generating.

We thank the reviewer for this assessment of the study's statistical power and for highlighting the distinction between prognostic modeling and mechanistic inference. We agree that the overall cohort size (n = 1,212) provides adequate statistical power for the primary objective of developing and evaluating multivariable prognostic models. In addition, the CCC subgroup (n = 191), with a 2-year mortality of 26%, represents one of the largest CCC cohorts with proteomic profiling reported to date, supporting the robustness of the prognostic analyses in this subgroup.

At the same time, we acknowledge that pathway enrichment analyses in etiologies with fewer events (e.g., alcoholic HF) may be underpowered for detailed biological interpretation. In response to the reviewer's suggestion, we have revised the manuscript to tone down mechanistic interpretations and to frame pathway findings more explicitly as exploratory.

Accordingly, the Discussion (Therapeutic Hypotheses and Future Directions subsection) now emphasizes that these analyses should be interpreted as hypothesis-generating, requiring further experimental and translational validation before clinical application. The revised text states:

"However, these are exploratory analyses and should be considered hypothesis-generating, with preclinical validation required before clinical studies."

These revisions clarify the distinction between the well-powered prognostic objectives of the study and the more exploratory nature of the biological pathway analyses.

5. **Were the correct statistical analyses used to support the conclusions?**

In general, yes. […] However, two methodological clarifications would strengthen reproducibility:

a. Whether batch effects in Olink NPX values were corrected.

We thank the reviewer for this comment and for highlighting the importance of clarifying how potential batch effects in Olink NPX values were handled. We have expanded the

Methods (Proteomic Panel Quantification subsection) to provide a more detailed description of the normalization and quality-control procedures applied to the proteomic data.

Specifically, we now clarify that NPX values were generated using Olink's standard normalization pipeline, which includes normalization to extension controls, log2 transformation, and plate-level adjustment using internal plasma controls. Additional quality-control procedures were applied according to Olink guidelines, including thresholds for matched counts, deviation limits for incubation and amplification controls, and predefined limits for negative control variation.

Importantly, all samples in this study were collected and processed at a single center and shipped together for analysis in a single Olink project run, which minimizes technical variability between plates and batches. For this reason, no additional post-hoc batch-effect correction beyond Olink's standard NPX normalization procedures was applied.

These methodological clarifications have been incorporated into the manuscript to improve transparency and reproducibility of the proteomic analyses.

b. Rationale for median imputation of missing data versus more distribution-aware methods.

We have expanded the Methods (Pre-processing subsection) to explain why median imputation was used for handling missing values. Specifically, several clinical variables exhibited skewed distributions and occasional extreme values, making the median a more robust estimator than the mean or regression-based approaches, which can be disproportionately influenced by outliers. Median imputation therefore helps ensure that imputed values remain within a clinically plausible range without artificially inflating predictor variance.

In addition, the overall proportion of missing data was very small (~2%) and sparsely distributed, with no evidence of systematic clustering across variables or samples. Under these conditions, simple imputation approaches are generally appropriate and unlikely to materially influence model estimates.

These clarifications have now been incorporated into the Methods section, improving the transparency of the preprocessing pipeline and the rationale for the selected imputation strategy.

6. **Does the analysis presented match the analysis plan?**

Yes. The analyses reported follow the methodology described in the Methods section. The authors clearly apply the planned sequence of steps: data preprocessing, train/test splitting, repeated validation permutations, feature selection, Naive Bayes classification, Cox proportional hazards modeling with time-dependent AUC/iAUC comparison, and pathway enrichment via GSEA. The progression from single-marker analyses (BNP) to multi-protein models (P9) and then to etiology-specific pathway enrichment matches the stated analytical framework. There is no evidence of unplanned exploratory analyses that would undermine

the integrity of the results, although the therapeutic "druggability" screen—while informative—could be more explicitly presented as exploratory and hypothesis-generating.

We thank the reviewer for this positive assessment of the alignment between the analytical plan and the analyses presented in the manuscript. As noted, the study follows the planned methodological sequence, including data preprocessing, train/test partitioning, repeated validation procedures, feature selection, predictive modeling, survival analyses with time-dependent discrimination metrics, and pathway enrichment analyses.

To further address the reviewer's comment regarding the interpretation of the therapeutic "druggability" screen, we have revised the manuscript to explicitly frame these analyses as exploratory and hypothesis-generating. The Discussion (Therapeutic Hypotheses and Future Directions subsection) now emphasizes that the identification of pathway-linked compounds is intended to generate biological hypotheses rather than to support therapeutic recommendations.

Specifically, the revised text states:

"However, these are exploratory analyses and should be considered hypothesis-generating, with preclinical validation required before clinical studies. Off-label use is not recommended before robust data exist on safety and efficacy of these drugs for HF, particularly considering the known risk of cardiotoxicity with anti-tumoral drugs and *T. cruzi* reactivation with immunosuppression in CCC."

These revisions clarify the intended scope of the druggability analyses and ensure that their interpretation remains consistent with the exploratory nature of the approach.

7. **Are the results clearly and completely presented?**

Overall, yes. […] Some areas could be strengthened for clarity:

a. Several mechanistic interpretations appear in the Results section (e.g., impaired trafficking in CCC) and would be more appropriate in the Discussion.

We thank the reviewer for this suggestion regarding the separation between results presentation and interpretation. In response, we revised the manuscript to ensure that the Results section focuses on the objective reporting of findings, while biological interpretation is reserved for the Discussion.

Specifically, we reformatted Figure 4 (now Fig. 5) and Supplementary Table S5 (now Suppl. Table S6), which present the pathway enrichment analyses. The Results section now strictly reports which pathways and biological processes are significantly enriched or downregulated, without mechanistic interpretation. The Discussion section has been correspondingly expanded to examine the potential biological implications of these findings.

These changes improve the clarity of the manuscript by maintaining a clearer distinction between data presentation and interpretation.

b. Greater clarity on effect sizes or confidence intervals in Figures/Tables would improve clinical interpretability.

We have revised the manuscript to include effect sizes and corresponding confidence intervals in several figures and tables where applicable. These additions provide a clearer representation of the magnitude and uncertainty of the observed effects, thereby improving the transparency and clinical interpretability of the reported results.

c. The presentation of therapeutic candidate findings in Table 3 would benefit from explicit

We revised both the Results and Discussion sections to explicitly emphasize the hypothesis-generating nature of the druggability analyses.

In the Results (Druggable targets for CCC subsection), the text now presents the identified compounds strictly as potential pathway-linked candidates emerging from the exploratory analysis, rather than as validated therapeutic options.

In addition, the Discussion (Therapeutic Hypotheses and Future Directions subsection) now explicitly states that these findings should be interpreted with caution and require preclinical validation before any clinical investigation. We also added explicit safety considerations, noting that off-label use cannot be recommended, particularly given the known cardiotoxicity of some anti-tumoral agents and the potential risk of *Trypanosoma cruzi* reactivation with immunosuppressive therapies in chronic Chagas cardiomyopathy.

These revisions ensure that the druggability results are clearly framed as exploratory biological insights intended to guide future research, rather than as clinical recommendations.

8. **Are the figures (Tables, Images) of sufficient quality for clarity?**

Yes. […] A few minor suggestions for clarity:

a. In Fig. 3, variability in etiologies with small sample sizes could be more clearly annotated to avoid overinterpretation.

We have updated Figure 3 to include confidence intervals for the subgroup estimates, allowing readers to more clearly visualize the degree of variability associated with etiologies with smaller sample sizes. This modification improves the interpretation of the results by explicitly indicating the greater uncertainty in subgroups with fewer observations, thereby reducing the risk of overinterpretation.

b. In some figures, axis labels and legends could be enlarged slightly for readability, particularly for non-specialist readers.

We have increased the font sizes of axis labels and figure legends in the main figures to improve readability. These adjustments aim to enhance clarity and accessibility, particularly for readers who may be less familiar with the specific analytical approaches presented.

c. Where possible, including effect sizes or confidence intervals in tables would strengthen the interpretation of differences.

We have revised the manuscript to include confidence intervals alongside the reported effect estimates, which improves the interpretation of the magnitude and uncertainty of the observed differences. Specifically, confidence intervals were added to Figures 2 and 3 and to Table 2, thereby strengthening the statistical transparency and interpretability of the reported results.

9. **Are the conclusions supported by the data presented?**

The primary conclusions—namely that a sparse nine-protein panel (P9) improves mortality prediction in most HFrEF etiologies but not in chronic Chagas cardiomyopathy (CCC), and that CCC exhibits a distinct circulating proteomic profile—are supported by the data presented. The survival analyses, classification results, and pathway enrichment findings consistently demonstrate a divergence between CCC and the other HF etiologies.

However, some mechanistic interpretations, particularly those attributing the lack of incremental prognostic value of P9 in CCC to impaired ER–Golgi trafficking and N-linked glycosylation, should be framed more cautiously. While pathway enrichment analysis suggests these processes may be affected, the proteomic platform used (which measures only secreted proteins) does not allow definitive conclusions about intracellular trafficking defects. These findings should be presented as hypotheses rather than established mechanisms.

We agree that some mechanistic interpretations required more cautious framing. Accordingly, we revised the Discussion to avoid causal language, emphasize the associative nature of the findings, and explicitly frame mechanistic interpretations as hypothesis-generating. In particular, the interpretation linking the attenuated incremental prognostic value of P9 in CCC to alterations in ER–Golgi trafficking and N-linked glycosylation has been rephrased to reflect that these associations arise from pathway enrichment analyses rather than direct functional evidence.

We also expanded the Limitations section to clarify that the Olink platform measures circulating proteins and therefore cannot directly assess intracellular trafficking processes or tissue-level remodeling. Additionally, we now emphasize that the observational design precludes causal inference and that mechanistic hypotheses will require future experimental validation.

Relevant revisions include the following statements added or modified in the Discussion:

"However, these are exploratory analyses and should be considered hypothesis-generating, with preclinical validation required before clinical studies. Off-label use is not recommended before robust data exist on safety and efficacy of these drugs for HF, particularly considering the known

risk of cardiotoxicity with anti-tumoral drugs and T. cruzi reactivation with immunosuppression in CCC."
"Second, our reliance on plasma proteomics with panels enriched for secreted proteins captures circulating signatures but does not directly reflect tissue-level remodeling or cellular heterogeneity, both of which are critical in CCC."
"In CCC, defects in the secretory and post-translational modification machinery may be associated with distorted proteomic signatures and attenuated additive prognostic value. Thus, while P9 expands predictive capacity in other HF etiologies where protein trafficking is likely intact, in CCC its incremental performance appears limited, leaving NT-proBNP as an equally effective prognostic marker."

Together, these revisions ensure that the conclusions remain fully aligned with the data while avoiding overinterpretation and clearly presenting mechanistic implications as hypotheses for future investigation.

10. **Are the limitations of analysis clearly described?**
The manuscript contains a Limitations section, but additional clarification is warranted. Specifically:
a. The authors should more explicitly acknowledge that Olink Explore is a panel-based, secretome-dependent platform that may not fully capture the biology of an infectious cardiomyopathy such as CCC, especially when the same analyses show potential impairment of protein trafficking in this etiology.

We agree that the limitations related to the panel-based and secretome-focused nature of the Olink platform should be more explicitly acknowledged, particularly in the context of CCC, where our analyses suggested potential alterations in protein trafficking pathways.

Accordingly, we expanded the Limitations section of the Discussion to clarify that Olink Explore primarily quantifies circulating proteins and therefore may not fully capture intracellular biological processes or tissue-level remodeling that are relevant to infectious cardiomyopathies such as CCC. These limitations are now explicitly discussed, as indicated in responses #2 and #9, including the following statement:
"Second, our reliance on plasma proteomics with panels enriched for secreted proteins captures circulating signatures but does not directly reflect tissue-level remodeling or cellular heterogeneity, both of which are critical in CCC."

This revision clarifies that while the proteomic platform is appropriate for prognostic modeling based on circulating biomarkers, it has inherent limitations for mechanistic inference, particularly regarding intracellular processes such as ER–Golgi trafficking.

b. The lack of an external validation cohort, particularly for CCC, should be more prominently addressed.

We performed external validation of the P9 model using data from the UK Biobank, and the corresponding methods, results, and supporting tables and figures were added to the revised manuscript. This analysis demonstrates that the P9 model improves mortality risk classification compared with NT-proBNP in an independent cohort, particularly when analyses are restricted to participants with comparable baseline risk.

Specifically, we added a new subsection in the Methods (External Validation) describing the UK Biobank cohort, inclusion criteria, and statistical procedures, including the use of time-dependent AUC estimated via the inverse probability of censoring weighting (IPCW) method of Uno et al., as well as additional performance metrics (F1-macro, iAUC, sensitivity, specificity, PPV, and NRI). The Results section (External Validation) now presents the full analysis, supported by Fig. 5 and Supplementary Tables S4–S5, showing that P9 improved classification performance relative to NT-proBNP in this independent dataset.

At the same time, we acknowledge that no independent proteomic cohorts of chronic Chagas cardiomyopathy currently exist, which prevented external replication of the CCC-specific findings. This limitation is now explicitly stated in the Discussion (Limitations and Future Perspectives).

Together, these additions clarify that the prognostic performance of the P9 model was externally validated, while also transparently acknowledging the current lack of independent proteomic datasets for CCC, which limits external validation of the etiology-specific findings.

c. The single-center origin of the cohort and potential geographic variation in T. cruzi DTUs may limit generalizability.

We agree that the single-center origin of the cohort and potential geographic variation in *T. cruzi* discrete typing units (DTUs) may influence the external applicability of the findings.

Accordingly, we expanded the Limitations section of the Discussion to explicitly acknowledge these factors. In particular, we note that information on acute Chagas infection, prior antiparasitic treatment, and T. cruzi strain/DTU was not available for this cohort, which may limit the ability to account for geographic and parasitological variability. We also emphasize that the absence of independent proteomic datasets in CCC currently precludes external validation of the CCC-specific findings, which may further limit generalizability.

The revised manuscript now includes the following statement in the Discussion (Limitations and Future Perspectives subsection):

“Additionally, information on acute Chagas diagnosis or treatment or T. cruzi strain were not available for this cohort. Moreover, the lack of available datasets on Chagas disease with proteomics precludes external validation and may limit the generalizability of our findings.”

These revisions clarify the contextual and geographic limitations of the current study while highlighting the need for future multicenter cohorts and geographically diverse CCC datasets to confirm and extend these findings.

d. The current data availability statement ("upon reasonable request") does not meet PLOS requirements and should be revised.

Because the dataset contains participant-level clinical and proteomic information, public deposition is not permitted under the ethical approvals and informed consent governing the GENIUS-HF cohort, as unrestricted access could compromise participant privacy.

Accordingly, we have revised the Data Availability Statement to clarify these restrictions and ensure transparency regarding data access. The manuscript now states:

Data Availability Statement:

"Due to legal and ethical restrictions related to participant privacy, the datasets generated and analyzed during the current study are not publicly available and cannot be shared."

This revision clarifies the constraints imposed by the institutional review board (IRB) protocol and participant confidentiality requirements, which prevent public release of the underlying individual-level data.

11. **Do the authors discuss how these data can be helpful to advance our understanding of the topic under study?**

Yes. […] This contribution is valuable; however, the discussion should more clearly delineate which insights are robust (prognostic performance) versus exploratory (mechanistic inference and therapeutic suggestions). The section proposing potential drug repurposing candidates should be explicitly framed as hypothesis-generating.

We agree that the manuscript should more clearly distinguish robust findings related to prognostic performance from exploratory mechanistic interpretations and therapeutic hypotheses.

Accordingly, we revised the manuscript to clarify that the drug-repurposing analysis is exploratory and hypothesis-generating, and that any therapeutic implications require rigorous preclinical validation before clinical investigation. We also expanded the Discussion of the prognostic performance of the P9 panel to emphasize the robustness of the predictive findings across HF etiologies and in external validation.

The revised text now explicitly frames the repurposing results as exploratory. For example:

Results section – Druggable Targets for CCC subsection:

"The filtered data summarizing main findings are shown in Table 3. Potential therapeutic repurposing options included cetuximab and panitumumab for fibrosis, tretinoin for integrin signaling, and prednisone (in case of a patient's broad immunosuppression) for CCC."

Discussion section – Therapeutic Hypotheses and Future Directions subsection:
"These findings should be considered exploratory and hypothesis-generating, and therefore require rigorous preclinical validation before any consideration of clinical investigation. Accordingly, our results should not be interpreted as supporting off-label therapeutic use. This caution is particularly important given the potential safety concerns associated with some of the compounds identified, including the risk of cardiotoxicity with certain antitumoral agents and the possibility of T. cruzi reactivation in the setting of immunosuppression in CCC."

These revisions clarify the distinction between the validated prognostic findings and the exploratory biological or therapeutic interpretations, ensuring that conclusions remain appropriately aligned with the data.

12. **Is public health relevance addressed?**
Yes. The manuscript addresses the substantial public health burden of CCC, emphasizing its disproportionately high mortality, its status as a neglected tropical disease, and the clinical need for improved risk stratification tools in endemic and non-endemic regions due to migration. The findings are relevant to clinicians, policymakers, and researchers seeking to improve the management of CCC within broader HF frameworks. Further strengthening the link to public health—e.g., discussing how biomarker-based risk stratification could impact resource allocation or follow-up strategies in low-resource settings—would enhance the translational significance.

We have expanded the Discussion to better articulate the potential public health relevance of improved biomarker-based risk stratification in heart failure, particularly in settings with constrained healthcare resources.

Specifically, we added language emphasizing that more accurate mortality prediction tools could help guide follow-up intensity, prioritize high-risk patients, and support more efficient allocation of healthcare resources, which is especially relevant for universal healthcare systems in low- and middle-income countries where CCC remains a major contributor to heart failure burden.

The following sentence was added to the Discussion section (P9 performance in overall HF subsection):
"These findings are relevant from a public health perspective, as accurate risk prediction could contribute to optimize resources allocation, particularly in universal healthcare systems in low- and middle-income countries, such as Brazil."

This revision strengthens the link between the study's prognostic findings and their potential impact on healthcare planning and disease management at the population level.

Editorial and Data Presentation Modifications?

a. Data availability statement
The current statement ("available upon reasonable request") does not meet PLOS policies. The authors should deposit the anonymized NPX matrix and key derived variables (or provide a justified exception). This is an essential editorial modification.

Because the dataset includes participant-level clinical and proteomic data, public deposition of the anonymized NPX matrix and derived variables is not permitted under the ethical approvals and informed consent governing the GENIUS cohort, as unrestricted sharing could compromise participant privacy.

In accordance with the protocol approved by the local Institutional Review Board (IRB), individual-level data cannot be made publicly available. We have therefore revised the Data Availability Statement to clarify these legal and ethical restrictions.

The manuscript now includes the following statement:

Data Availability Statement:

"Due to legal and ethical restrictions related to participant privacy, the datasets generated and analyzed during the current study are not publicly available and cannot be shared."

This revision ensures transparency regarding the constraints imposed by the IRB protocol and participant confidentiality requirements, which prevent public release of the underlying participant-level data.

b. Clarify the distinction between BNP and NT-proBNP
Throughout the text, BNP/NT-proBNP terminology is used interchangeably. The authors should explicitly state which protein was quantified via Olink and maintain consistent terminology.

In the original version, the terms BNP and NT-proBNP were occasionally used interchangeably, which could create ambiguity. In this study, NT-proBNP was the biomarker measured, quantified through the Olink Explore Cardiometabolic panel, and values were analyzed using NPX (normalized protein expression) units.

We have therefore revised the manuscript to use consistent NT-proBNP terminology throughout the text and clarified the measurement method in the Methods section.

The manuscript now includes the following clarification:

Methods section – Proteomic Panel Quantification subsection:

"NT-proBNP measurements reported in the analyses were based on Olink measurements, as part of the Cardiometabolic panel."

This revision ensures consistent nomenclature and clearer description of the biomarker assay used in the analyses.

c. Improve clarity of figure legends and axis labels

c1. Some figures (particularly Fig. 2 and Fig. 3) would benefit from slightly enlarged fonts and clearer labeling of units and axes.

We revised the affected figures to improve readability, including increasing font sizes for axis labels and legends and clarifying axis units where applicable, particularly in Figures 2 and 3. These adjustments improve figure clarity and facilitate interpretation for both specialist and non-specialist readers.

c2. Variability in small subgroups (e.g., alcoholic HF) should be annotated to avoid overinterpretation.

To improve interpretation and avoid potential overinterpretation of estimates derived from smaller subgroups (e.g., alcoholic HF), we added confidence intervals to the relevant figures. This allows readers to clearly visualize the greater variability and uncertainty associated with estimates in smaller strata, thereby providing a more transparent representation of subgroup-level results.

d. Refine presentation of pathway analyses. Results in Fig. 4 and Suppl. Table S3 could be made more readable by: grouping pathways more clearly by higher-level biological categories, ensuring NES directionality is immediately visible, streamlining color schemes to help non-specialists.

To improve clarity and interpretability of the pathway enrichment results, we revised the presentation of the pathway analyses. Specifically, we updated Figure 4 (now Fig. 6) and Supplementary Table S3 (now Suppl. Table S6) to improve readability, including clearer grouping of pathways and a more structured presentation of enrichment results. These changes make the biological organization of the pathways more transparent and facilitate interpretation for both specialist and non-specialist readers.

e. Editorial restructuring of mechanistic interpretations
Portions of mechanistic interpretation currently appear in the Results section. These should be moved to the Discussion to maintain a clean separation between results and interpretation.

In the revised manuscript, we reorganized the relevant sections to maintain a clear separation between results and interpretation. Specifically, mechanistic interpretations that previously appeared in the Results section were moved to the Discussion, where their biological implications are more appropriately addressed.

This restructuring ensures that the Results section focuses on the presentation of observed findings, while interpretative and mechanistic considerations are discussed in the Discussion section, as detailed in response #7a.

f. Therapeutic repurposing table (Table 3)

Table 3 should be introduced explicitly as exploratory/hypothesis-generating to avoid overstating mechanistic certainty.

We revised the manuscript to explicitly frame the therapeutic repurposing findings presented in Table 3 as exploratory and hypothesis-generating, thereby avoiding any implication of mechanistic certainty or clinical recommendation.

Specifically, we toned down the language in both the Results and Discussion sections and added explicit cautionary statements regarding the preliminary nature of these findings and the need for further validation.

Results section – Druggable targets for CCC subsection:

“The filtered data summarizing main findings are shown in Table 3. Potential therapeutic repurposing options included cetuximab and panitumumab for fibrosis, tretinoin for integrin signaling, and prednisone (in case of a patient’s broad immunosuppression) for CCC.”

Discussion section – Therapeutic Hypotheses and Future Directions subsection:

“These findings should be considered exploratory and hypothesis-generating, and therefore require rigorous preclinical validation before any consideration of clinical investigation. Accordingly, our results should not be interpreted as supporting off-label therapeutic use. This caution is particularly important given the potential safety concerns associated with some of the compounds identified, including the risk of cardiotoxicity with certain antitumoral agents and the possibility of T. cruzi reactivation in the setting of immunosuppression in CCC.”

These revisions ensure that Table 3 is clearly presented as a hypothesis-generating analysis rather than a validated therapeutic framework.

Reviewer #3:

1. **Abstract: it is mentioned that "P9 improved integrated AUC over time (iAUC) by 10.3%, from 74% to 81% (p<0.05)." but it is not clear compared to what. Please specify.**

In the original version, the improvement in iAUC was not explicitly stated relative to the comparator. We have revised the Abstract to clarify that the comparison is against NT-proBNP alone.

In addition, following methodological revisions requested by reviewers, the Naive Bayes model was replaced by logistic regression, which resulted in updated performance estimates. The Abstract has been updated accordingly.

Revised Abstract text:

"Feature-selection models identified a nine-protein panel (P9: C1QA, CCL4, REN, EGLN1, COL9A1, GP1BA, ITM2A, CNPY2, NT-proBNP) that improved F1-macro by 20% compared with NT-proBNP alone (0.674 vs. 0.560), and improved integrated time-dependent discrimination for 2-year mortality relative to NT-proBNP."

This revision clarifies the reference comparator and reflects the updated modeling framework used in the revised manuscript.

2. **The abstract should also mention that the patient cohort is from Brazil.**

To improve clarity and contextualize the study population, we have updated the Abstract to explicitly state that the cohort is from Brazil.

The Abstract now includes the following sentence:

"Baseline plasma samples from 1,212 adults with heart failure with reduced ejection fraction (HFrEF, LVEF <50%) from Brazil were analyzed using the Olink Explore cardiometabolic and inflammation panels, quantifying 734 proteins."

This revision ensures that the geographic origin of the cohort is clearly identified in the Abstract, which is particularly relevant given the inclusion of patients with chronic Chagas cardiomyopathy.

3. **The title is poorly informative and could be improved.**

To improve clarity and better reflect the scope and key findings of the manuscript, we have revised the title to more clearly convey both the prognostic and biological aspects of the study.

The updated title is:

"Plasma Proteomics Improves Risk Prediction in Heart Failure and Reveals Unique Biology in Chronic Chagas Cardiomyopathy."

This revision highlights the two central contributions of the study: improved prognostic modeling in heart failure using proteomics and the identification of distinct biological features associated with chronic Chagas cardiomyopathy.

4. **Study cohort: please specify if "HF patients with EF below 50%" refers to LVEF.**

We have revised the Methods section to explicitly state that EF refers to left ventricular ejection fraction (LVEF).

The text now reads:

Methods section:

"This single-center prospective cohort study included HF patients with LVEF below 50% referred to the cardiology clinic at the Heart Institute (InCor) of the Hospital das Clínicas, University of São Paulo Medical School (HC-FMUSP), Brazil, between August 2012 and January 2015."

This revision ensures clear specification of the echocardiographic parameter used to define the study population.

5. **Please expand on "Chagas disease etiology was confirmed by serological evidence of IgG antibodies to Trypanosoma cruzi" by providing information on test performed. Also, since time may be a key factor affecting patient prognosis, please explain how time since infection/diagnosis was taken into account in the models for assessing risk. There is no mention of antiparasitic treatment for CCC patients. Had any of these patients been treated before? With what drug? When? These are critical confounding factors and these patients may need to be further stratified accordingly. Please explain why no healthy controls were included for comparison, as these may increase model performance to identify biomarkers.**

We thank the reviewer for these points regarding Chagas disease diagnosis, potential confounding factors, and study design. We have revised the manuscript to clarify the diagnostic methods and to acknowledge the limitations related to missing historical clinical information.

First, we expanded the description of Chagas disease diagnosis in the Methods section. In this cohort, positive serology was established using an immunoenzymatic assay and confirmed by an indirect immunofluorescence assay, consistent with standard diagnostic practice.

Methods section – Study design and cohort subsection:

"Chagas disease etiology was confirmed by serological evidence of IgG antibodies to Trypanosoma cruzi by immunoenzymatic and indirect immunofluorescence assays."

Second, regarding time since infection, prior diagnosis, and antiparasitic therapy, reliable information was unfortunately not available for this cohort. Most patients were referred to a tertiary cardiology center many years after the acute infection, which is frequently asymptomatic or nonspecific and therefore often not diagnosed at the time it occurs. Consequently, dates of infection or prior treatment (e.g., benznidazole) were not consistently documented, precluding their inclusion in the prognostic models. We have now explicitly acknowledged this limitation in the Discussion.

Discussion section – Limitations and Future Perspectives subsection:

“Additionally, information on acute Chagas diagnosis or treatment or T. cruzi strain were not available for this cohort.”

Finally, healthy controls were not included because the primary objective of the study was prognostic modeling in patients with established heart failure. Since healthy individuals are unlikely to experience mortality events during the relatively short follow-up period, their inclusion would not meaningfully contribute to the evaluation of mortality risk prediction models, which require outcome events for model training and validation.

Together, these revisions clarify the diagnostic methods used, acknowledge important clinical data limitations, and justify the cohort design in the context of the study’s prognostic objectives.

6. **Please provide details on what “Baseline” means, and how patient follow-up was performed and for how long.**

In this study, baseline refers to the first clinical visit after referral to the cardiology clinic, at which time the echocardiographic evaluation and plasma collection for proteomic analysis were performed. We have clarified this definition in the Methods section.

Patient follow-up was conducted through routine clinical care for a period of two years, and outcomes were ascertained through hospital electronic medical records and the Brazilian National Death Records system, where all deaths are legally registered. These details have now been explicitly described in the manuscript.

Methods section – Study design and cohort subsection:

“Blood samples were collected, and echocardiograms were performed by board-certified specialists as part of the first patients’ visit to the cardiology clinic. Patients with LVEF <50% assessed were enrolled.”

“Patients were followed for 2 years and deaths were ascertained continuously through hospital electronic records and the National Death Records repository, as all deaths in Brazil are legally required to be reported.”

These additions clarify both the definition of baseline and the follow-up procedures used to ascertain outcomes.

7. **Please provide details on when LVEF was measured as “before enrollment” is too vague, and again time may be a critical issue and confounding factor for disease progression and mortality.**

LVEF was measured at baseline, defined as the first clinical visit after referral to the cardiology clinic, when both echocardiographic evaluation and plasma sampling were performed. We have revised the manuscript to make this timing explicit.

Methods section – Study design and cohort subsection:

"Blood samples were collected, and echocardiograms were performed by board-certified specialists as part of the first patients' visit to the cardiology clinic. Patients with LVEF <50% assessed were enrolled."

This clarification ensures that the timing of LVEF measurement relative to study enrollment is clearly defined.

8. Please provide details on blood collection and sample processing for the proteomic analysis.

We have expanded the Methods section to more clearly describe the procedures for blood collection, processing, and storage prior to proteomic analysis.

The manuscript now includes the following description:

Methods section – Proteomic Panel Quantification subsection:

"Peripheral venous blood was collected into EDTA tubes, centrifuged to obtain plasma, aliquoted to avoid repeated freeze–thaw cycles, stored at −80 °C, and shipped on dry ice for biomarker quantification."

These additions clarify the pre-analytical handling of samples, ensuring transparency regarding procedures used to preserve sample integrity prior to proteomic profiling.

9. The proteomic dataset needs to be deposited in the appropriate public database (NCBI or similar).

The participant-level proteomic and clinical datasets cannot be deposited in a public repository because they contain sensitive health information and their unrestricted release would conflict with the ethical approvals and informed consent governing the GENIUS cohort, as approved by the local Institutional Review Board (IRB).

To ensure transparency, we have revised the Data Availability Statement in the manuscript to clearly explain these restrictions. The revised statement reads:

Data Availability Statement:

"Due to legal and ethical restrictions related to participant privacy, the datasets generated and analyzed during the current study are not publicly available and cannot be shared."

This revision clarifies that IRB and participant confidentiality requirements prevent public deposition of the individual-level dataset.

10. Tables should be provided as text files, not images.

In the revised manuscript, all tables have been converted to editable, cell-based table objects within the manuscript file, rather than embedded as images. This ensures that the tables are fully accessible, searchable, and compliant with journal formatting requirements.

Table 1: Please explain what * refers to in the table. It seems from the text that multiple student t tests were performed to compare groups, which is inappropriate. Please performed the appropriate statistical analysis (ANOVA/Kruskal Wallis with post hoc tests as appropriate). Does ejection fraction refer to LVEF? Please clarify.

We revised the statistical comparisons in Table 1 to ensure appropriate between-group testing. Specifically, continuous variables are now compared using one-way ANOVA followed by Tukey's HSD post hoc test, while categorical variables are analyzed using the Chi-square test.

We also clarified the meaning of the asterisks (*) and explicitly defined ejection fraction as left ventricular ejection fraction (LVEF) in the table legend.

The revised legend now reads:

Table 1 – Legend:

"Statistical differences between the five main etiologies were assessed using one-way ANOVA for continuous parameters and the Chi-square test for categorical counts. Asterisks () denote specific classes identified via post-hoc analysis (Tukey's HSD or standardized residuals) as significantly deviating from the respective expected distribution (p = 0.05). LVEF: left ventricular ejection fraction."*

11. Ethnic categories in the text (Methods) and Table 1 are different, please make consistent.

In the revised manuscript, we updated the ethnic category nomenclature in Table 1 to ensure full consistency with the terminology used in the Methods section. This correction harmonizes the classification across the manuscript and improves clarity for readers.

12. Complete performance results for the training and the testing datasets should be shown in Table 2 for a rigorous evaluation of the models. Also, while stratification according to LVEF is informative, additional stratifications would strengthen the analysis. For example according to NYHA functional class, or time since initial infection/diagnosis of heart disease.

In the revised manuscript, we expanded the presentation of the results to provide a more comprehensive evaluation of model performance. Specifically, Table 2 now includes more detailed performance metrics for the models, allowing clearer comparison between training and testing datasets.

In addition, to strengthen the clinical characterization of the cohort, we updated Table 1 to include stratification by NYHA functional class, alongside the previously reported stratifications by etiology and LVEF. This provides a more complete description of disease severity within the cohort and improves the clinical interpretability of the analyses.

Information on time since infection or diagnosis of Chagas disease was unfortunately not available for this cohort, as many patients were referred to the tertiary cardiology center many years after the acute infection phase. This limitation is now acknowledged in the Discussion.

13. **Table S3: It is not clear which pathways are associated with which etiologies. Please modify the table to clearly indicate etiologies and how the indicated pathways associate with these and their statistical significance for each etiology.**

To improve clarity, we restructured Supplementary Table S3 (now Suppl. Table S6) so that the associated etiology is clearly indicated in the first column for each pathway entry. This revision allows readers to more easily identify which pathways are enriched in each heart failure etiology and their corresponding statistical significance.

The updated table now explicitly reports etiology, pathway name, biological process category, normalized enrichment score (NES), FDR-adjusted q-values, and leading genes, facilitating interpretation of pathway–etiology associations.

14. **Additional limitations that should be mentioned in the discussion are (1) the lack of information on T. cruzi strains infecting the CCC cases, which may modulate disease severity and mortality and (2) that the extrapolation of the results to non-brazilian patients remains unclear.**

We expanded the Limitations section of the Discussion to explicitly acknowledge both the absence of information on T. cruzi strain diversity and the potential limitations in generalizing the findings beyond the Brazilian population studied.

The manuscript now includes the following statement in the Discussion (Limitations and Future Perspectives subsection):

"Additionally, information on acute Chagas diagnosis or treatment or T. cruzi strain were not available for this cohort. Moreover, the lack of available datasets on Chagas disease with proteomics precludes external validation and may limit the generalizability of our findings."

This revision clarifies that parasite genetic diversity and geographic variation, which are known to influence disease severity and progression in Chagas disease, could not be assessed in this cohort and may affect the extrapolation of the findings to other populations.

15. **Also the discussion would benefit from more extensive comparison of the results with previous studies on plasma biomarkers of CCC. See for example Garg et al, PLOS NTD 2016; Bautista Lopez et al, Am Heart J 2013; Ambrosio et al, Front Immunol 2024; Jaimes_Dueñez et al., Clin Appl Thromb Hemost 2024; Choudhuri et al, Microbiol Spectr 2021 among others.**

We agree that situating our findings within the context of prior CCC biomarker studies strengthens the interpretation of the results. Accordingly, we expanded the Discussion to

include a broader comparison with previously published studies investigating circulating biomarkers and molecular pathways in CCC.

We note that many prior studies have used different methodological approaches, including small targeted biomarker panels, analyses of peripheral blood mononuclear cells (PBMCs), extracellular vesicles, or microRNA profiling, which limits direct comparison with our large-scale plasma proteomic analysis. Nevertheless, the biological signals identified in our study are broadly consistent with prior reports highlighting immune activation, inflammatory signaling, and structural remodeling processes as key features of CCC pathophysiology.

The following text was added to the Discussion section (CCC as a Unique Case within HF subsection):

"Most prior studies evaluating biomarkers in chronic Chagas cardiomyopathy (CCC) have relied on small-scale targeted assays or alternative molecular approaches, such as analyses of microRNAs or extracellular vesicles, which limits direct comparison with our results. Nevertheless, our findings are broadly consistent with those of a prior study that applied mass-spectrometry–based proteomics to peripheral blood mononuclear cells (PBMCs) from patients with CCC. That study reported upregulation of pathways related to cytoskeletal disassembly and structural disorganization, as well as processes involved in immune cell aggregation, recruitment, activation, and migration. Together, these observations support a biological framework in which immune activation and cellular structural remodeling play central roles in the pathophysiology of CCC, and provide orthogonal evidence that aligns with the proteomic signals observed in our analysis."

This addition provides contextual comparison with prior CCC biomarker studies while acknowledging methodological differences that limit direct comparability.
