# Supplementary material for: Plasma proteomics improves risk prediction in heart failure and reveals unique biology in chronic chagas cardiomyopathy
Source: PLoS Negl Trop Dis. 2026 Jun 8;20(6):e0014370. doi: 10.1371/journal.pntd.0014370 (PMC13245751; doi:10.1371/journal.pntd.0014370)
Supplement: Attachment — Submitted filename: 26_05_09_Answers_Reviewers_jk.docx [file pntd.0014370.s019.docx]

**Response to Review**

We appreciate the insightful comments and suggestions from the Reviewers. This letter details the changes to our manuscript that we have made in response to each of the comments and suggestions provided.

**Reviewer #1**

1. Given the high-dimensional feature selection, the lack of bootstrap optimism correction or nested cross-validation leaves uncertainty about how much overfitting remains in the derivation cohort. External validation speaks to transportability, but not to optimism introduced during model development. This should either be addressed directly or more clearly acknowledged as a limitation.

*We have now included a Bootstrap optimism correction analysis, using 1,000 bootstrap resampling. Limited overfitting was evidenced by the small correction obtained (averaged ΔF1-macro difference of 0.013, based on the bootstrap resampling strategy). The test set performance (0.674) was within the corrected bootstrap estimate (95% IC = [0.603, 0.699]). We added the relevant information associated with this analysis in sections M&M (sub-section “Assessment of overfitting”), and Results (sub-section “P9: A Generalizable HFrEF Model”).*

Edits to the manuscript:

*Methods* section:

*Assessment of overfitting*

*Optimism correction of P9 model was performed using 1,000 bootstrap resampling, with each bootstrap used to train the model and then evaluated on both the bootstrapped and original train sets, separately. The difference in F1-macro scores provided an optimism estimate, averaged across iterations and subtracted from the apparent performance to obtain the corrected F1-macro. Validation was carried out on the test set.*

*Results* section:

*Bootstrap optimism correction for P9 demonstrated limited overfitting, as evidenced by the small correction obtained (averaged ΔF1-macro difference of 0.013, based on a 1,000 bootstrap resampling strategy). The test set performance (0.674) was within the corrected bootstrap estimate (95% IC = [0.603, 0.699]).*

2. Related to this, feature selection stability was not evaluated. I understand the authors’ focus on prediction rather than biomarker discovery, but with correlated proteomic predictors, instability in the selected panel remains a concern. Some indication of how often the selected proteins appear across resamples would help assess robustness.

*To address this, we performed 1,000 repeated stratified train/validation resampling iterations and evaluated the stability of the standardized logistic regression coefficients for all proteins included in the final nine-marker panel. Across resamples, the selected proteins showed consistent coefficient directions and relatively stable effect distributions, with median coefficients and 95% percentile intervals summarized in the revised Suppl. Fig. S4-E. These analyses support that the final panel is not driven by highly unstable or arbitrarily selected predictors, despite the expected redundancy and correlation structure inherent to proteomic data. We also show in Suppl. Fig. S4-D that P9’s F1-macro is superior than any other random set of [NT-proBNP + other eight markers]. The relevant information was included in section Results (sub-section “P9: A Generalizable HFrEF Model”), and in the updated Suppl. Fig. S4.*

Edits to the manuscript:

*Results* section:

*A comparison of P9 against other random subsets of nine proteins (one of them always being NT-proBNP) in the train set showed that its F1-macro was indeed superior to any of the 9-marker-sets tested (Suppl. Fig. S4-D). Standardized coefficients for all nine markers of the P9 model were also obtained, highlighting the relative importance of each protein to the model (Suppl. Fig. S4-E).*

**Suppl. Fig. S4. Sequential machine learning pipeline for proteomic feature selection.**

***Legend:*** *(A) Genetic Algorithm-based logistic regression optimized F1-macro across 734 proteins using iterative selection, crossover, and mutation, yielding 322 pre-selected features. (B) Bayesian variable selection via Stochastic Search Variable Selection was applied to the reduced feature set using MCMC sampling, and proteins were ranked by Posterior Inclusion Probability (PIP). (C) Successively inclusive feature sets were evaluated to identify the optimal number of proteins maximizing F1-macro, resulting in a final panel of nine proteins (shaded area: permutation-based 95% CI). (D) P9 compared against 1000 randomly generated protein sets composed of NT-proBNP plus eight randomly selected markers. For each set, logistic regression performance was evaluated across 10 independent stratified train/validation splits, and the median F1-macro recorded. (E) Stability of standardized logistic regression coefficients for the nine-protein panel across 1,000 repeated stratified resampling iterations. Coefficient distributions were summarized by median and 95% percentile intervals.*

3. The comparison with alternative modeling approaches is also still limited. Reporting F1-macro distributions is helpful, but not sufficient for the question at hand. The main performance metrics used in the manuscript (iAUC, calibration, Brier score) should be shown for competing models in the test set to better justify the choice of logistic regression.

*We included a performance analysis of the ten original machine learning models on the P9 marker set specifically, for each of three metrics across 100 bootstraps (F1-macro, iAUC, Brier score). The results showed that logistic regression (as well as naive Bayes) continued to be a strong choice regarding this smaller set of proteins, compared to the whole Proteomic set, hence reinforcing logistic regression as a reliable model. The relevant information was added in the main text, in section Results (sub-section “Machine Learning model”). Additionally, Suppl. Fig. S3 was extended to show the performance of these three metrics across the ten models.*

Edits to the manuscript:

*Results* section

*When all ten machine learning models were evaluated using the final marker set obtained after feature selection, logistic regression was identified as a strong-performing model across three different metrics (Suppl. Fig. S3-B).*

***Suppl. Fig. S3.*** ***Distribution of F1-macro scores for each machine learning model.***

***Legend:*** *(A) Distribution of F1-macro scores for each machine learning model (ML) tested, presented as box plots. The evaluation methodology involved 1,000 random patient permutations in the Train set. For each run, 70% of patient data was randomly selected for model training, and the resulting F1-macro score was calculated on the held-out 30% validation set. Models are ranked top-to-bottom according to their median performance score. (B) Reassessing all ten ML models for P9 on the Test set for three different metrics (across 100 bootstraps).*

4. Finally, calibration reporting still feels a bit incomplete. The addition of calibration plots and Brier scores is appreciated, but standard summaries such as calibration-in-the-large and calibration slope are not clearly reported, and it is not obvious whether calibration was assessed in clinically relevant subgroups (particularly CCC), where miscalibration risk is higher.

*In the revised manuscript, we now explicitly report calibration-in-the-large (CITL) and calibration slope values for both the overall cohort and clinically relevant etiological subgroups, including CCC/Chagas cardiomyopathy and non-Chagas patients (Suppl. Fig. S5-A). Overall, the P9 model showed CITL and slope values closer to the ideal references of 0 and 1, respectively, compared with NT-proBNP alone. In the Chagas subgroup, both models showed positive CITL values and slopes >1, whereas in the non-Chagas subgroup both models showed negative CITL values and slopes <1, although deviations were less pronounced for P9. These additions were included to provide a more complete assessment of subgroup-specific calibration behavior and potential miscalibration risk. The relevant information was added to section Results (sub-section “P9: A Generalizable HFrEF Model”). Besides, Suppl. Fig. S5 was extended to include the results for these metrics.*

Edits to the manuscript:

*Results* section

*Calibration metrics indicated better agreement between predicted and observed risks for the P9 model than for NT-proBNP alone. In the full cohort, P9 had CITL and calibration slope values closer to the ideal references of 0 and 1, respectively (CITL: −0.210 vs. −0.500; slope: 0.870 vs. 0.663). In the Chagas subgroup, both models showed positive CITL values and slopes >1, suggesting systematic calibration deviation with under-dispersed predictions. In the non-Chagas subgroup, both models showed negative CITL values and slopes <1, indicating risk miscalibration with overfitted predictions; however, these deviations were less pronounced for P9 (CITL = −0.463; slope = 0.761) than for NT-proBNP alone (CITL = −0.833; slope = 0.514) (Suppl. Fig. S5-A).*

***Suppl. Fig. S5. Clinical utility of P9 vs. BNP for 2-year mortality.***

***Legend:*** *(A) Calibration metrics for P9 and NT-proBNP in the overall study sample, Chagas subgroup and non-Chagas subgroup. (B) Calibration plot showing predicted versus observed mortality for the BNP model. The dotted diagonal line represents perfect calibration. The dotted red curve with squares shows raw predictions, while the solid blue curve with circles shows calibrated probabilities. Brier scores are also shown. (C) P9 Calibration plot, following the same strategy. (D) Decision Curve Analysis (DCA) comparing the P9 (orange) and BNP (blue) models. The "Treat All" strategy (red dotted line) starts and ends at the outcome prevalence (~16%), while the "Treat None" strategy (black line) remains at zero. A model is clinically useful when its curve sits above both baselines. (E) Δ Net Benefit across threshold probabilities: 95% CIs (shaded area) indicate significant superiority for P9 where the intervals exclude zero (based on 1,000 bootstraps). Numeric labels indicate the net reduction in unnecessary interventions per 100 patients achieved by using P9 instead of BNP without missing true cases. For example, at a 5% threshold, P9's efficiency gain means avoiding 146.6 unnecessary interventions per 100 patients compared to BNP while maintaining equal safety.*
