# Supplementary material for: Plasma proteomics improves risk prediction in heart failure and reveals unique biology in chronic chagas cardiomyopathy
Source: PLoS Negl Trop Dis. 2026 Jun 8;20(6):e0014370. doi: 10.1371/journal.pntd.0014370 (PMC13245751; doi:10.1371/journal.pntd.0014370)
Supplement: Peer review history

# Peer review: Decision Letter 0

**DOI:** 10.1371/journal.pntd.0014370.r001

**Authors:** Guilherme L Werneck, Karina Gómez

7 Jan 2026

PNTD-D-25-01654

Proteomic Risk Prediction in Chronic Chagas Cardiomyopathy Reveals Unique Biology

PLOS Neglected Tropical Diseases

Dear Dr. Krieger

Thank you for submitting your manuscript to PLOS Neglected Tropical Diseases. After careful consideration, we feel that it has merit but does not fully meet PLOS Neglected Tropical Diseases's publication criteria as it currently stands. Therefore, we invite you to submit a revised version of the manuscript that addresses the points raised during the review process.

Please submit your revised manuscript within by 90 days. If you will need more time than this to complete your revisions, please reply to this message or contact the journal office at plosntds@plos.org. When you're ready to submit your revision, log on to https://www.editorialmanager.com/pntd/ and select the 'Submissions Needing Revision' folder to locate your manuscript file.

Please include the following items when submitting your revised manuscript:

* A letter that responds to each point raised by the editor and reviewer(s). You should upload this letter as a separate file labeled 'Response to Reviewers'. This file does not need to include responses to any formatting updates and technical items listed in the 'Journal Requirements' section below.

* A marked-up copy of your manuscript that highlights changes made to the original version. You should upload this as a separate file labeled 'Revised Manuscript with Track Changes'.

* An unmarked version of your revised paper without tracked changes. You should upload this as a separate file labeled 'Manuscript'.

If you would like to make changes to your financial disclosure, competing interests statement, or data availability statement, please make these updates within the submission form at the time of resubmission. Guidelines for resubmitting your figure files are available below the reviewer comments at the end of this letter.

We look forward to receiving your revised manuscript.

Kind regards,

Karina Andrea Gómez, Ph. D.

Academic Editor

PLOS Neglected Tropical Diseases

Guilherme Werneck

Section Editor

PLOS Neglected Tropical Diseases

Shaden Kamhawi

co-Editor-in-Chief

PLOS Neglected Tropical Diseases

orcid.org/0000-0003-4304-636XX

Paul Brindley

co-Editor-in-Chief

PLOS Neglected Tropical Diseases

orcid.org/0000-0003-1765-0002

**Journal Requirements:**

1) Please provide an Author Summary. This should appear in your manuscript between the Abstract (if applicable) and the Introduction, and should be 150-200 words long. The aim should be to make your findings accessible to a wide audience that includes both scientists and non-scientists. Sample summaries can be found on our website under Submission Guidelines:

https://journals.plos.org/plosntds/s/submission-guidelines#loc-parts-of-a-submission

2) We noticed that you used the phrase 'data not shown' in the manuscript. We do not allow these references, as the PLOS data access policy requires that all data be either published with the manuscript or made available in a publicly accessible database. Please amend the supplementary material to include the referenced data or remove the references.

3) Tables should not be uploaded as individual files. Please remove these files and include the Tables in your manuscript file as editable, cell-based objects. For more information about how to format tables, see our guidelines:

https://journals.plos.org/plosntds/s/tables

4) We notice that your supplementary figures are uploaded with the file type 'Figure'. Please amend the file type to 'Supporting Information'. Please ensure that each Supporting Information file has a legend listed in the manuscript after the references list.

5) Some material included in your submission may be copyrighted. According to PLOSu2019s copyright policy, authors who use figures or other material (e.g., graphics, clipart, maps) from another author or copyright holder must demonstrate or obtain permission to publish this material under the Creative Commons Attribution 4.0 International (CC BY 4.0) License used by PLOS journals. Please closely review the details of PLOSu2019s copyright requirements here: PLOS Licenses and Copyright. If you need to request permissions from a copyright holder, you may use PLOS's Copyright Content Permission form.

Please respond directly to this email and provide any known details concerning your material's license terms and permissions required for reuse, even if you have not yet obtained copyright permissions or are unsure of your material's copyright compatibility. Once you have responded and addressed all other outstanding technical requirements, you may resubmit your manuscript within Editorial Manager.

Potential Copyright Issues:

- Figure 4. Please confirm whether you drew the images / clip-art within the figure panels by hand. If you did not draw the images, please provide (a) a link to the source of the images or icons and their license / terms of use; or (b) written permission from the copyright holder to publish the images or icons under our CC BY 4.0 license. Alternatively, you may replace the images with open source alternatives. See these open source resources you may use to replace images / clip-art:

- https://commons.wikimedia.org

- https://openclipart.org/.

6) In the online submission form, you indicated that "The data that support the findings of this study are available from the corresponding author upon reasonable request.". All PLOS journals now require all data underlying the findings described in their manuscript to be freely available to other researchers, either

1. In a public repository

2. Within the manuscript itself

3. Uploaded as supplementary information.

This policy applies to all data except where public deposition would breach compliance with the protocol approved by your research ethics board. If your data cannot be made publicly available for ethical or legal reasons (e.g., public availability would compromise patient privacy), please explain your reasons by return email and your exemption request will be escalated to the editor for approval. Your exemption request will be handled independently and will not hold up the peer review process, but will need to be resolved should your manuscript be accepted for publication. One of the Editorial team will then be in touch if there are any issues.

7) Please amend your detailed Financial Disclosure statement. This is published with the article. It must therefore be completed in full sentences and contain the exact wording you wish to be published.

- State the initials, alongside each funding source, of each author to receive each grant. For example: "This work was supported by the National Institutes of Health (####### to AM; ###### to CJ) and the National Science Foundation (###### to AM)."

- State what role the funders took in the study. If the funders had no role in your study, please state: "The funders had no role in study design, data collection and analysis, decision to publish, or preparation of the manuscript.".

If you did not receive any funding for this study, please simply state: u201cThe authors received no specific funding for this work.u201d

**Reviewers' Comments:**

Reviewer's Responses to Questions

**Key Review Criteria Required for Acceptance?**

As you describe the new analyses required for acceptance, please consider the following:

**Methods**

-Are the objectives of the study clearly articulated with a clear testable hypothesis stated?

-Is the study design appropriate to address the stated objectives?

-Is the population clearly described and appropriate for the hypothesis being tested?

-Is the sample size sufficient to ensure adequate power to address the hypothesis being tested?

-Were correct statistical analysis used to support conclusions?

-Are there concerns about ethical or regulatory requirements being met?

Reviewer #1: Essential analyses / items to include in revision

1) Table with N and number of deaths per etiology and by LVEF strata

2) Add calibration plots and decision curve analysis (P9 vs BNP vs MAGGIC) in the test set, and present iAUC with 95% CI.

3) Present event counts and EPV and perform optimism correction (bootstrap) or nested CV to estimate unbiased performance

4) Provide stability analysis for feature selection (frequency of inclusion across resamples).

5) Explicit description of BNP/NT-proBNP assay and Olink assay handling (batch, QC).

6) Provide full details of PyMC model and genetic algorithm (priors, iterations, parameters), plus code availability (GitHub link) and TRIPOD checklist.

7) Tone down drug repurposing claims, add explicity safety caveats and tempered language for repurposing suggestions (no clinical recommendations without preclinical/controlled validation). Also add explicit safety caveats regarding immunosuppression in CCC.

Reviewer #2: Are the objectives of the study clearly articulated with a clear, testable hypothesis stated?

Yes, the overarching objectives are clearly described: to evaluate whether a sparse proteomic panel can improve 2-year mortality prediction in HFrEF across etiologies, and to determine whether chronic Chagas cardiomyopathy (CCC) exhibits distinct prognostic or biological features. While the manuscript does not state a single explicit hypothesis sentence, the testable hypotheses are implicit and adequately operationalized (e.g., that proteomic signatures improve risk prediction beyond BNP, and that CCC displays etiology-specific pathways). Stating the hypotheses more explicitly would improve clarity.

Is the study design appropriate to address the stated objectives?

Overall, yes. A large, prospective cohort (GENIUS-HF) with adjudicated HF etiology and longitudinal mortality follow-up is appropriate for prognostic modeling and proteomic comparison across etiologies. The use of Olink Explore panels is suitable for circulating biomarker discovery and risk prediction. However, for mechanistic interpretation—especially claims about “unique CCC biology”—the platform’s limitations should be acknowledged. Because Olink measures secreted proteins and the study finds downregulation of ER-to-Golgi trafficking and N-glycosylation specifically in CCC, the method may introduce a structural bias in this etiology. This does not compromise the prognostic aim but affects mechanistic depth.

Is the population clearly described and appropriate for the hypothesis being tested?

Yes. The cohort is well characterized, with detailed demographic, clinical, socioeconomic, and echocardiographic data. CCC diagnosis by dual T. cruzi serology is appropriate. Etiology definitions follow standard cardiology criteria. The population is appropriate for prognostic evaluation; however, it is restricted to a single center in Brazil, which may influence generalizability, particularly for CCC where regional variation in T. cruzi DTUs exists. This limitation should be noted more explicitly in the Methods or Limitations.

Is the sample size sufficient to ensure adequate power to address the hypothesis being tested?

Yes for the primary prognostic objective. The full sample (n=1,212) provides adequate power for multivariable modeling, and the CCC subgroup (n=191; 26% 2-year mortality) is one of the largest published to date. However, power for pathway analyses in etiologies with fewer events (e.g., alcoholic HF) is limited, which the authors appropriately acknowledge. For CCC, the sample size is sufficient for prognostic modeling but places limits on deeper mechanistic inference—another reason to present findings as hypothesis-generating.

Were the correct statistical analyses used to support the conclusions?

In general, yes. The authors employ appropriate analyses including:

– train/test split with repeated permutations,

– Naive Bayes classifier selected objectively,

– Cox proportional hazards models,

– time-dependent AUC and integrated AUC (iAUC),

– TOST equivalence testing,

– FDR-adjusted logistic regression for etiology-specific proteins,

– GSEA with multiple databases.

These methods are rigorous and well aligned with the study aims.

However, two methodological clarifications would strengthen reproducibility:

1. Whether batch effects in Olink NPX values were corrected.

2. Rationale for median imputation of missing data versus more distribution-aware methods.

Neither issue invalidates the results but should be clarified.

Are there concerns about ethical or regulatory requirements being met?

No concerns. The study reports IRB approval (CAAE - 70162117.0.0000.0068) and informed consent.

Reviewer #3: see general comments

**********

**Results**

-Does the analysis presented match the analysis plan?

-Are the results clearly and completely presented?

-Are the figures (Tables, Images) of sufficient quality for clarity?

Reviewer #1: (No Response)

Reviewer #2: Does the analysis presented match the analysis plan?

Yes. The analyses reported follow the methodology described in the Methods section. The authors clearly apply the planned sequence of steps: data preprocessing, train/test splitting, repeated validation permutations, feature selection, Naive Bayes classification, Cox proportional hazards modeling with time-dependent AUC/iAUC comparison, and pathway enrichment via GSEA. The progression from single-marker analyses (BNP) to multi-protein models (P9) and then to etiology-specific pathway enrichment matches the stated analytical framework. There is no evidence of unplanned exploratory analyses that would undermine the integrity of the results, although the therapeutic “druggability” screen—while informative—could be more explicitly presented as exploratory and hypothesis-generating.

Are the results clearly and completely presented?

Overall, yes. The results are presented in a logical and comprehensive manner. Key outcomes such as differential mortality across etiologies, performance metrics of BNP vs. P9, and the identification of 128 CCC-specific proteins are described clearly. The survival analyses, classification performance, and enrichment results are internally consistent and easy to follow. Pathway findings are well summarized, and the distinction between CCC and non-CCC etiologies is effectively demonstrated.

Some areas could be strengthened for clarity:

1. Several mechanistic interpretations appear in the Results section (e.g., impaired trafficking in CCC) and would be more appropriate in the Discussion.

2. Greater clarity on effect sizes or confidence intervals in Figures/Tables would improve clinical interpretability.

3. The presentation of therapeutic candidate findings in Table 3 would benefit from explicit framing as exploratory rather than validated biological conclusions.

Are the figures (Tables, Images) of sufficient quality for clarity?

Yes. Figures and tables are generally of high technical quality and appropriate for the content. Kaplan–Meier curves (Fig. 1), classifier performance plots (Fig. 2), and etiology-specific comparisons (Fig. 3) are clear and visually interpretable. The pathway enrichment plot and network visualization (Fig. 4) are informative and help summarize complex results. Supplementary tables are comprehensive and contain the necessary detail.

A few minor suggestions for clarity:

– In Fig. 3, variability in etiologies with small sample sizes could be more clearly annotated to avoid overinterpretation.

– In some figures, axis labels and legends could be enlarged slightly for readability, particularly for non-specialist readers.

– Where possible, including effect sizes or confidence intervals in tables would strengthen the interpretation of differences.

These are refinements rather than substantive limitations.

Reviewer #3: see general comments

**********

**Conclusions**

-Are the conclusions supported by the data presented?

-Are the limitations of analysis clearly described?

-Do the authors discuss how these data can be helpful to advance our understanding of the topic under study?

-Is public health relevance addressed?

Reviewer #1: (No Response)

Reviewer #2: Are the conclusions supported by the data presented?

The primary conclusions—namely that a sparse nine-protein panel (P9) improves mortality prediction in most HFrEF etiologies but not in chronic Chagas cardiomyopathy (CCC), and that CCC exhibits a distinct circulating proteomic profile—are supported by the data presented. The survival analyses, classification results, and pathway enrichment findings consistently demonstrate a divergence between CCC and the other HF etiologies.

However, some mechanistic interpretations, particularly those attributing the lack of incremental prognostic value of P9 in CCC to impaired ER–Golgi trafficking and N-linked glycosylation, should be framed more cautiously. While pathway enrichment analysis suggests these processes may be affected, the proteomic platform used (which measures only secreted proteins) does not allow definitive conclusions about intracellular trafficking defects. These findings should be presented as hypotheses rather than established mechanisms.

Are the limitations of analysis clearly described?

The manuscript contains a Limitations section, but additional clarification is warranted. Specifically:

– The authors should more explicitly acknowledge that Olink Explore is a panel-based, secretome-dependent platform that may not fully capture the biology of an infectious cardiomyopathy such as CCC, especially when the same analyses show potential impairment of protein trafficking in this etiology.

– The lack of an external validation cohort, particularly for CCC, should be more prominently addressed.

– The single-center origin of the cohort and potential geographic variation in T. cruzi DTUs may limit generalizability.

– The current data availability statement (“upon reasonable request”) does not meet PLOS requirements and should be revised.

Addressing these points would strengthen the transparency and scientific balance of the conclusions.

Do the authors discuss how these data can be helpful to advance our understanding of the topic under study?

Yes. The authors argue convincingly that their findings highlight the need for etiology-specific biomarker strategies rather than universal HF models. They emphasize how CCC’s atypical proteomic signature challenges existing prognostic tools and opens avenues for precision medicine. They also describe how the unique pathways enriched in CCC may guide future mechanistic and translational studies.

This contribution is valuable; however, the discussion should more clearly delineate which insights are robust (prognostic performance) versus exploratory (mechanistic inference and therapeutic suggestions). The section proposing potential drug repurposing candidates should be explicitly framed as hypothesis-generating.

Is public health relevance addressed?

Yes. The manuscript addresses the substantial public health burden of CCC, emphasizing its disproportionately high mortality, its status as a neglected tropical disease, and the clinical need for improved risk stratification tools in endemic and non-endemic regions due to migration. The findings are relevant to clinicians, policymakers, and researchers seeking to improve the management of CCC within broader HF frameworks. Further strengthening the link to public health—e.g., discussing how biomarker-based risk stratification could impact resource allocation or follow-up strategies in low-resource settings—would enhance the translational significance.

Reviewer #3: see general comments

**********

**Editorial and Data Presentation Modifications?**

Use this section for editorial suggestions as well as relatively minor modifications of existing data that would enhance clarity. If the only modifications needed are minor and/or editorial, you may wish to recommend “Minor Revision” or “Accept”.

Reviewer #1: (No Response)

Reviewer #2: Overall, the manuscript is clearly written and well organized, but several editorial and presentation refinements would substantially enhance clarity and alignment with PLOS NTDs standards:

1. Data availability statement

The current statement (“available upon reasonable request”) does not meet PLOS policies. The authors should deposit the anonymized NPX matrix and key derived variables (or provide a justified exception). This is an essential editorial modification.

2. Clarify the distinction between BNP and NT-proBNP

Throughout the text, BNP/NT-proBNP terminology is used interchangeably. The authors should explicitly state which protein was quantified via Olink and maintain consistent terminology.

3. Improve clarity of figure legends and axis labels

- Some figures (particularly Fig. 2 and Fig. 3) would benefit from slightly enlarged fonts and clearer labeling of units and axes.

- Variability in small subgroups (e.g., alcoholic HF) should be annotated to avoid overinterpretation.

4. Refine presentation of pathway analyses

Results in Fig. 4 and Suppl. Table S3 could be made more readable by:

- grouping pathways more clearly by higher-level biological categories,

- ensuring NES directionality is immediately visible,

- streamlining color schemes to help non-specialists.

5. Editorial restructuring of mechanistic interpretations

Portions of mechanistic interpretation currently appear in the Results section. These should be moved to the Discussion to maintain a clean separation between results and interpretation.

6. Therapeutic repurposing table (Table 3)

Table 3 should be introduced explicitly as exploratory/hypothesis-generating to avoid overstating mechanistic certainty.

These changes are editorial in nature and do not require new data, but they are important to ensure clarity and compliance with journal standards.

Reviewer #3: see general comments

**********

**Summary and General Comments**

Use this section to provide overall comments, discuss strengths/weaknesses of the study, novelty, significance, general execution and scholarship. You may also include additional comments for the author, including concerns about dual publication, research ethics, or publication ethics. If requesting major revision, please articulate the new experiments that are needed.

Reviewer #1: This is an important and novel paper. The authors used deep plasma proteomics (Olink Explore panels) in a large Brazilian HFrEF cohort (n=1212; 191 with CCC) and derived a P9 predicting 2-yr mortality. P9 improves discrimination over BNP and the MAGGIC score in most HF etiologies, especially idiopathic and hypertensive HF, but not in CCC; there BNP alone performs as well. Pathway analyses show CCC has a distinct signature (fibrosis/integrin signaling, innate immune activation, and downregulation of ER-to-Golgi trafficking and glycosylation). The findings are interesting and clinically relevant, but several methodological and interpretative issues must be addressed before this is ready for publication.

Major strengths include: It addresses a high-need, under-studied problem in a large (single-center though) cohort with careful outcome ascertainment (national death registry). Also the deep proteomic coverage (∼734 proteins) with a translational pipeline (feature selection, time-dependent modeling, and pathway enrichment, and potential therapeutic-target screening). A provocative and useful observation. It shows good translational thinking (nominating pathways/druggable targets) while keeping the study hypothesis-generating.

Major concerns/actions needed:

- External validation and overfitting risk: conclusions rely on internal train/test split only. With high-dimensional proteomics there is substantial overfitting risk. CCC subgroup is moderate in size and may be underpowered for complex models. If an independent cohort exists, validate P9 and the CCC finding there and report iAUC, calibration, and reclassification. If no cohort is available, at minimum perform stronger internal validation: repeated/nested cross-validation or bootstrap optimism correction for iAUC and report optimism-corrected estimates with 95% CI. Temper claims about generalizability in the manuscript until external validation is done.

- Naive Bayes was chosen, but NB assumes conditional independence of predictors — unlikely with correlated proteins. The PyMC to genetic algorithm pipeline is not sufficiently described (priors, hyperparameters, convergence, selection stability). Please provide a clear pipeline diagram; report comparative performance (iAUC, calibration, Brier score) of other sensible methods (penalized Cox/logistic, XGBoost, random forest) in the test set. Give full PyMC model details (priors, iterations, diagnostics) and genetic algorithm parameters. Report selection stability (frequency each protein appears across resamples/bootstraps). If NB remains best, explain why and discuss limitations of the independence assumption.

- iAUC differences are reported as percent gains but without confidence intervals and clear statement whether gains are absolute points or relative percent. TOST equivalence margin needs justification. Methods for handling censoring in time-dependent ROC are not fully described. Please report absolute iAUC values with 95% CI and exact p-values; show time-dependent AUCs at 6/12/24 months. State the statistical method used for time-dependent AUC and how censoring was handled. Provide exact TOST results and justify the chosen equivalence margin (0.05 absolute iAUC).

- Only discrimination metrics are shown. For clinical translation we need calibration and net benefit. The study will benefit by adding calibration plots and metrics (calibration-in-the-large, calibration slope) for P9 and BNP in the test set, overall and by etiology (esp. CCC). Please add decision-curve analysis to show net benefit across clinically relevant thresholds. Consider reporting NRI or clinical impact measures.

- Samples with <99% protein data were excluded; that’s strict and may bias results. Median imputation for missing proteomic values is simplistic and could be inappropriate if missingness is not random. The number and characteristics of excluded samples are not reported. Please report the N excluded and their characteristics. Compare included vs excluded. Use multiple imputation for clinical covariates and consider principled imputation approaches for proteomic data (or at least sensitivity analyses comparing median imputation with other approaches). Discuss missingness assumptions.

- The manuscript uses BNP and NT-proBNP terms inconsistently. Since part of the mechanistic argument rests on peptide processing/glycosylation, assay details matter. Choose consistent nomenclature, and clearly describe the clinical natriuretic assay used (BNP or NT-proBNP: assay manufacturer, LOD, units). If both measurements or Olink NPX for that analyte were used, clarify how they relate.

- Mapping from measured proteins to gene sets and to drugs is nontrivial. Also recommending prednisone or EGFR monoclonals for Chagas patients without strong caveats is risky: systemic immunosuppression can reactivate T. cruzi and EGFR inhibitors have cardiovascular toxicities. Include method details for protein to gene mapping, report number of proteins measured per pathway and full GSEA tables in supplement. Reframe druggability findings as hypothesis-generating and add explicit safety cautions (parasitic reactivation with immunosuppression, known cardiac toxicities of anticancer agents). Tone down direct therapeutic recommendations throughout the manuscript.

- The number of deaths per etiology is not clearly shown in the main text. Multi-protein models in small subgroups risk low events-per-variable. Include a table listing N and deaths per etiology and per LVEF stratum. Calculate events-per-variable for P9 and other models. Also discuss limitations where EPV is low and consider simpler models in small subgroups.

- Concerns about potential confounding such as socioeconomic status, treatment differences, antiparasitic therapy, etc... CCC patients have different socioeconomic characteristics, less comorbidity, and possibly different treatments. Antiparasitic therapy (e.g, benznidazole) history is not reported. These factors could confound proteomic signals or outcomes. Please report antiparasitic treatment rates, device therapy (ICD/CRT), medication doses/adherence if available. In addition, include sensitivity analyses adjusting for socioeconomic markers and treatment differences.

Please avoid any causal language. The manuscript sometimes implies causality from association (for instance: “trafficking defects attenuate proteomic signal”). Rewrite to emphasize associations and hypothesis generation; reserve causal claims for future mechanistic studies.

Minor points, proofreading and presentation:

- Define NPX on first use.

- Reconcile LVEF definitions with current nomenclature (HFrEF typically ≤40%; HFmrEF 41–49%). If you choose <50% to define inclusion, justify it up front and consider standard strata in analyses.

- In the methods, give details on sample handling (collection, processing, storage, freeze-thaw cycles), batch randomization and batch-correction method for Olink NPX. Also, add software versions and random seeds for reproducibility.

- Replace “shuffling’s” with “shuffles.” Fix spacing (e.g., “FDR-corrected”). Replace vague p-values like “p << 0.01” with exact values when possible.

- For figures, add number-at-risk tables to KM plots, calibration plots, and decision-curve graphs. For performance figures include CIs and sample sizes used for each comparison.

- In S1–S4, ensure UniProt IDs, median NPX per group, and full GSEA tables (with # measured genes per pathway) are present. Include a table showing how often each P9 protein was selected across resamples.

- Finally, “Millipede analysis” needs a citation or brief explanation.

Overall, this is a high-value study with strong translational potential. The main scientific claim that CCC is biologically distinct and that universal proteomic models may not generalize to Chagas disease is plausible and interesting. Addressing the major issues above (especially validation, model transparency, calibration and safety wording around repurposing) will make the paper much stronger and more convincing.

Reviewer #2: This manuscript presents a well-executed and clinically relevant proteomic analysis in a large HFrEF cohort, with particular emphasis on chronic Chagas cardiomyopathy (CCC), a neglected and highly lethal etiology. The study identifies a nine-protein panel (P9) that improves 2-year mortality prediction in most HF etiologies but not in CCC, where BNP alone retains similar predictive value. This is a significant and novel contribution to the field, highlighting the need for etiology-specific risk stratification and drawing important attention to CCC as a biologically distinct condition.

Strengths of the study include:

– a large, well-characterized prospective cohort,

– rigorous analytical methodology (multiple ML techniques, Cox models, iAUC, TOST tests),

– detailed proteomic assessment (734 proteins),

– identification of 128 CCC-specific protein associations and 14 enriched pathways.

Weaknesses and limitations:

– The proteomic platform used (Olink Explore) is panel-based and secretome-dependent. This introduces potential systematic bias in CCC, since the study itself reports downregulation of ER–Golgi trafficking and N-glycosylation specifically in this etiology. These findings make mechanistic interpretations less definitive and should be reframed as hypotheses rather than conclusions.

– Generalizability is limited by the single-center nature of the cohort and lack of external validation.

– The drug repurposing component is exploratory and should be clearly presented as such.

– Data availability must be aligned with PLOS requirements.

Novelty and significance:

The demonstration that CCC uniquely fails to benefit from multi-protein prognostic models is both novel and highly relevant, especially given the disproportionate mortality burden of Chagas disease. The study advances understanding by showing that CCC’s proteomic profile diverges sharply from other HF etiologies and that universal biomarker strategies may be inadequate.

Recommendation regarding revision level:

I recommend Major Revision, primarily to adjust the interpretive framing:

– soften mechanistic claims about “unique CCC biology,”

– explicitly acknowledge potential bias introduced by the secretome-dependent platform,

– clarify limitations and generalizability,

– moderate the therapeutic implications.

No new laboratory experiments are required. The requested changes are conceptual, interpretive, and editorial ones.

Reviewer #3: The study entitled “Proteomic Risk Prediction in Chronic Chagas Cardiomyopathy Reveals Unique Biology” aims at identifying plasma biomarker for the prediction of mortality associated with Chronic Chagas Cardiomyopathy, compared with unrelated heart failure. It is of key relevance given the lack of prognostic biomarkers, which complicates patient care. However, the study presents multiple shortcomings that need to be addressed to ensure more rigorous conclusions.

Specific comments:

1. Abstract: it is mentioned that “P9 improved integrated AUC over time (iAUC) by 10.3%, from 74% to 81% (p<0.05).” but it is not clear compared to what. Please specify.

2. The abstract should also mention that the patient cohort is from Brazil.

3. The title is poorly informative and could be improved.

4. Study cohort: please specify if “HF patients with EF below 50%” refers to LVEF.

Please expand on “Chagas disease etiology was confirmed by serological evidence of IgG antibodies to Trypanosoma cruzi” by providing information on test performed. Also, since time may be a key factor affecting patient prognosis, please explain how time since infection/diagnosis was taken into account in the models for assessing risk. There is no mention of antiparasitic treatment for CCC patients. Had any of these patients been treated before? With what drug? When? These are critical confounding factors and these patients may need to be further stratified accordingly. Please explain why no healthy controls were included for comparison, as these may increase model performance to identify biomarkers.

5. Please provide details on what “Baseline” means, and how patient follow-up was performed and for how long.

6. Please provide details on when LVEF was measured as “before enrollment” is too vague, and again time may be a critical issue and confounding factor for disease progression and mortality.

7. Please provide details on blood collection and sample processing for the proteomic analysis.

8. The proteomic dataset needs to be deposited in the appropriate public database (NCBI or similar).

9. Tables should be provided as text files, not images.

10. Table 1: Please explain what * refers to in the table. It seems from the text that multiple student t tests were performed to compare groups, which is inappropriate. Please performed the appropriate statistical analysis (ANOVA/Kruskal Wallis with post hoc tests as appropriate). Does ejection fraction refer to LVEF? Please clarify.

11. Ethnic categories in the text (Methods) and Table 1 are different, please make consistent.

12. Complete performance results for the training and the testing datasets should be shown in Table 2 for a rigorous evaluation of the models. Also, while stratification according to LVEF is informative, additional stratifications would strengthen the analysis. For example according to NYHA functional class, or time since initial infection/diagnosis of heart disease.

13. Table S3: It is not clear which pathways are associated with which etiologies. Please modify the table to clearly indicate etiologies and how the indicated pathways associate with these and their statistical significance for each etiology.

14. Additional limitations that should be mentioned in the discussion are (1) the lack of information on T. cruzi strains infecting the CCC cases, which may modulate disease severity and mortality and (2) that the extrapolation of the results to non-brazilian patients remains unclear.

15. Also the discussion would benefit from more extensive comparison of the results with previous studies on plasma biomarkers of CCC. See for example Garg et al, PLOS NTD 2016; Bautista Lopez et al, Am Heart J 2013; Ambrosio et al, Front Immunol 2024; Jaimes_Dueñez et al., Clin Appl Thromb Hemost 2024; Choudhuri et al, Microbiol Spectr 2021 among others.

**********

PLOS authors have the option to publish the peer review history of their article (what does this mean?). If published, this will include your full peer review and any attached files.

If you choose “no”, your identity will remain anonymous but your review may still be made public.

**Do you want your identity to be public for this peer review?** For information about this choice, including consent withdrawal, please see our Privacy Policy.

Reviewer #1: **Yes:** Giovane R. Sousa

Reviewer #2: No

Reviewer #3: No

[NOTE: If reviewer comments were submitted as an attachment file, they will be attached to this email and accessible via the submission site. Please log into your account, locate the manuscript record, and check for the action link "View Attachments". If this link does not appear, there are no attachment files.]

**Figure resubmission:**

While revising your submission, we strongly recommend that you use PLOS’s NAAS tool (https://ngplosjournals.pagemajik.ai/artanalysis) to test your figure files. NAAS can convert your figure files to the TIFF file type and meet basic requirements (such as print size, resolution), or provide you with a report on issues that do not meet our requirements and that NAAS cannot fix.

After uploading your figures to PLOS’s NAAS tool - https://ngplosjournals.pagemajik.ai/artanalysis, NAAS will process the files provided and display the results in the "Uploaded Files" section of the page as the processing is complete. If the uploaded figures meet our requirements (or NAAS is able to fix the files to meet our requirements), the figure will be marked as "fixed" above. If NAAS is unable to fix the files, a red "failed" label will appear above. When NAAS has confirmed that the figure files meet our requirements, please download the file via the download option, and include these NAAS processed figure files when submitting your revised manuscript.

**Reproducibility:**

To enhance the reproducibility of your results, we recommend that authors of applicable studies deposit laboratory protocols in protocols.io, where a protocol can be assigned its own identifier (DOI) such that it can be cited independently in the future. Additionally, PLOS ONE offers an option to publish peer-reviewed clinical study protocols. Read more information on sharing protocols at https://plos.org/protocols?utm_medium=editorial-email&utm_source=authorletters&utm_campaign=protocols

# Author comment: Author response to Decision Letter 1

**DOI:** 10.1371/journal.pntd.0014370.r002

16 Mar 2026

Attachment Submitted filename: 26_03_14_ResponsetoReview.pdf

# Peer review: Decision Letter 1

**DOI:** 10.1371/journal.pntd.0014370.r003

**Authors:** Guilherme L Werneck, Karina Gómez

24 Apr 2026

PNTD-D-25-01654R1

Plasma Proteomics Improves Risk Prediction in Heart Failure and Reveals Unique Biology in Chronic Chagas Cardiomyopathy

PLOS Neglected Tropical Diseases

Dear Dr. Krieger,

Thank you for submitting your manuscript to PLOS Neglected Tropical Diseases. After careful consideration, we feel that it has merit but does not fully meet PLOS Neglected Tropical Diseases's publication criteria as it currently stands. Therefore, we invite you to submit a revised version of the manuscript that addresses the points raised during the review process.

Please submit your revised manuscript by May 24 2026 11:59PM. If you will need more time than this to complete your revisions, please reply to this message or contact the journal office at plosntds@plos.org. When you're ready to submit your revision, log on to https://www.editorialmanager.com/pntd/ and select the 'Submissions Needing Revision' folder to locate your manuscript file.

Please include the following items when submitting your revised manuscript:

* A letter that responds to each point raised by the editor and reviewer(s). You should upload this letter as a separate file labeled 'Response to Reviewers'. This file does not need to include responses to any formatting updates and technical items listed in the 'Journal Requirements' section below.

* A marked-up copy of your manuscript that highlights changes made to the original version. You should upload this as a separate file labeled 'Revised Manuscript with Track Changes'.

* An unmarked version of your revised paper without tracked changes. You should upload this as a separate file labeled 'Manuscript'.

If you would like to make changes to your financial disclosure, competing interests statement, or data availability statement, please make these updates within the submission form at the time of resubmission. Guidelines for resubmitting your figure files are available below the reviewer comments at the end of this letter.

We look forward to receiving your revised manuscript.

Kind regards,

Karina Andrea Gómez, Ph. D.

Academic Editor

PLOS Neglected Tropical Diseases

Guilherme Werneck

Section Editor

PLOS Neglected Tropical Diseases

Shaden Kamhawi

co-Editor-in-Chief

PLOS Neglected Tropical Diseases

orcid.org/0000-0003-4304-636XX

Paul Brindley

co-Editor-in-Chief

PLOS Neglected Tropical Diseases

orcid.org/0000-0003-1765-0002

**Reviewers' comments:**

Reviewer's Responses to Questions

**Key Review Criteria Required for Acceptance?**

As you describe the new analyses required for acceptance, please consider the following:

**Methods**

-Are the objectives of the study clearly articulated with a clear testable hypothesis stated?

-Is the study design appropriate to address the stated objectives?

-Is the population clearly described and appropriate for the hypothesis being tested?

-Is the sample size sufficient to ensure adequate power to address the hypothesis being tested?

-Were correct statistical analysis used to support conclusions?

-Are there concerns about ethical or regulatory requirements being met?

Reviewer #1: Mostly strong methods, but internal validation, feature-selection stability, and fuller reporting of competing models need addressing, and a few clarifications on population and power would help.

Reviewer #3: The authors have substantially modified the manuscript and addressed comments adequately.

**********

**Results**

-Does the analysis presented match the analysis plan?

-Are the results clearly and completely presented?

-Are the figures (Tables, Images) of sufficient quality for clarity?

Reviewer #1: Results are generally clear and well-presented, with high-quality figures, but some key metrics for competing models and calibration subgroups should be added to fully match the analysis plan.

Reviewer #3: The authors have substantially modified the manuscript and addressed comments adequately.

**********

**Conclusions**

-Are the conclusions supported by the data presented?

-Are the limitations of analysis clearly described?

-Do the authors discuss how these data can be helpful to advance our understanding of the topic under study?

-Is public health relevance addressed?

Reviewer #1: The conclusions are generally supported by the data, though some claims about model robustness should be a bit more cautious given the lack of internal validation and assessment of feature-selection stability. The limitations are mentioned, but the potential impact of overfitting and subgroup miscalibration could be clearer. The discussion does a good job showing how these findings add to understanding of proteomic risk prediction in heart failure. Public health relevance is touched on, but could be emphasized more by explaining how these models might eventually help guide personalized care—while noting they aren’t ready for clinical use yet. Overall, the conclusions are reasonable, but a few tweaks would make them better aligned with the results.

Reviewer #3: The authors have substantially modified the manuscript and addressed comments adequately.

**********

**Editorial and Data Presentation Modifications?**

Use this section for editorial suggestions as well as relatively minor modifications of existing data that would enhance clarity. If the only modifications needed are minor and/or editorial, you may wish to recommend “Minor Revision” or “Accept”.

Reviewer #1: A few minor phrasing tweaks could help readability, but nothing that would affect interpretation.

Reviewer #3: The authors have substantially modified the manuscript and addressed comments adequately.

**********

**Summary and General Comments**

Use this section to provide overall comments, discuss strengths/weaknesses of the study, novelty, significance, general execution and scholarship. You may also include additional comments for the author, including concerns about dual publication, research ethics, or publication ethics. If requesting major revision, please articulate the new experiments that are needed.

Reviewer #1: The manuscript has improved substantially, particularly with the addition of external validation, calibration analyses, and decision-curve results. Many of the prior concerns have been addressed. That said, a few issues remain that still affect the strength of the modeling claims.

The main one is internal validation and overfitting. The UK Biobank analysis is a useful addition, but it does not replace proper internal validation of the full modeling pipeline. Given the high-dimensional feature selection, the lack of bootstrap optimism correction or nested cross-validation leaves uncertainty about how much overfitting remains in the derivation cohort. External validation speaks to transportability, but not to optimism introduced during model development. This should either be addressed directly or more clearly acknowledged as a limitation.

Related to this, feature selection stability was not evaluated. I understand the authors’ focus on prediction rather than biomarker discovery, but with correlated proteomic predictors, instability in the selected panel remains a concern. Some indication of how often the selected proteins appear across resamples would help assess robustness.

The comparison with alternative modeling approaches is also still limited. Reporting F1-macro distributions is helpful, but not sufficient for the question at hand. The main performance metrics used in the manuscript (iAUC, calibration, Brier score) should be shown for competing models in the test set to better justify the choice of logistic regression.

Finally, calibration reporting still feels a bit incomplete. The addition of calibration plots and Brier scores is appreciated, but standard summaries such as calibration-in-the-large and calibration slope are not clearly reported, and it is not obvious whether calibration was assessed in clinically relevant subgroups (particularly CCC), where miscalibration risk is higher.

These points do not require a major reworking of the study, but they do matter for how confidently the model can be interpreted.

Reviewer #3: The authors have substantially modified the manuscript and addressed comments adequately.

**********

PLOS authors have the option to publish the peer review history of their article (what does this mean?). If published, this will include your full peer review and any attached files.

If you choose “no”, your identity will remain anonymous but your review may still be made public.

**Do you want your identity to be public for this peer review?** For information about this choice, including consent withdrawal, please see our Privacy Policy.

Reviewer #1: No

Reviewer #3: No

[NOTE: If reviewer comments were submitted as an attachment file, they will be attached to this email and accessible via the submission site. Please log into your account, locate the manuscript record, and check for the action link "View Attachments". If this link does not appear, there are no attachment files.]

**Figure resubmission:**

While revising your submission, we strongly recommend that you use PLOS’s NAAS tool (https://ngplosjournals.pagemajik.ai/artanalysis) to test your figure files. NAAS can convert your figure files to the TIFF file type and meet basic requirements (such as print size, resolution), or provide you with a report on issues that do not meet our requirements and that NAAS cannot fix.

After uploading your figures to PLOS’s NAAS tool - https://ngplosjournals.pagemajik.ai/artanalysis, NAAS will process the files provided and display the results in the "Uploaded Files" section of the page as the processing is complete. If the uploaded figures meet our requirements (or NAAS is able to fix the files to meet our requirements), the figure will be marked as "fixed" above. If NAAS is unable to fix the files, a red "failed" label will appear above. When NAAS has confirmed that the figure files meet our requirements, please download the file via the download option, and include these NAAS processed figure files when submitting your revised manuscript.

**Reproducibility:**

To enhance the reproducibility of your results, we recommend that authors of applicable studies deposit laboratory protocols in protocols.io, where a protocol can be assigned its own identifier (DOI) such that it can be cited independently in the future. Additionally, PLOS ONE offers an option to publish peer-reviewed clinical study protocols. Read more information on sharing protocols at https://plos.org/protocols?utm_medium=editorial-email&utm_source=authorletters&utm_campaign=protocols

# Author comment: Author response to Decision Letter 2

**DOI:** 10.1371/journal.pntd.0014370.r004

11 May 2026

Attachment Submitted filename: 26_05_09_Answers_Reviewers_jk.docx

# Editor report: Decision Letter 2

**DOI:** 10.1371/journal.pntd.0014370.r005

**Authors:** Guilherme L Werneck, Karina Gómez

12 May 2026

Dear Authors,

We are pleased to inform you that your manuscript 'Plasma Proteomics Improves Risk Prediction in Heart Failure and Reveals Unique Biology in Chronic Chagas Cardiomyopathy ' has been provisionally accepted for publication in PLOS Neglected Tropical Diseases.

Before your manuscript can be formally accepted you will need to complete some formatting changes, which you will receive in a follow up email. A member of our team will be in touch with a set of requests.

Please note that your manuscript will not be scheduled for publication until you have made the required changes, so a swift response is appreciated.

IMPORTANT: The editorial review process is now complete. PLOS will only permit corrections to spelling, formatting or significant scientific errors from this point onwards. Requests for major changes, or any which affect the scientific understanding of your work, will cause delays to the publication date of your manuscript.

Should you, your institution's press office or the journal office choose to press release your paper, you will automatically be opted out of early publication. We ask that you notify us now if you or your institution is planning to press release the article. All press must be co-ordinated with PLOS.

Thank you again for supporting Open Access publishing; we are looking forward to publishing your work in PLOS Neglected Tropical Diseases.

Best regards,

Karina Andrea Gómez, Ph. D.

Academic Editor

PLOS Neglected Tropical Diseases

Guilherme Werneck

Section Editor

PLOS Neglected Tropical Diseases

Shaden Kamhawi

co-Editor-in-Chief

PLOS Neglected Tropical Diseases

orcid.org/0000-0003-4304-636XX

Paul Brindley

co-Editor-in-Chief

PLOS Neglected Tropical Diseases

orcid.org/0000-0003-1765-0002

***********************************************************

p.p1 {margin: 0.0px 0.0px 0.0px 0.0px; line-height: 16.0px; font: 14.0px Arial; color: #323333; -webkit-text-stroke: #323333}span.s1 {font-kerning: none

# Editor report: Acceptance letter

**DOI:** 10.1371/journal.pntd.0014370.r006

**Authors:** Guilherme L Werneck, Karina Gómez

Dear Dr. Krieger,

We are delighted to inform you that your manuscript, "Plasma Proteomics Improves Risk Prediction in Heart Failure and Reveals Unique Biology in Chronic Chagas Cardiomyopathy ," has been formally accepted for publication in PLOS Neglected Tropical Diseases.

We have now passed your article onto the PLOS Production Department who will complete the rest of the publication process. All authors will receive a confirmation email upon publication.

The corresponding author will soon be receiving a typeset proof for review, to ensure errors have not been introduced during production. Please review the PDF proof of your manuscript carefully, as this is the last chance to correct any scientific or type-setting errors. Please note that major changes, or those which affect the scientific understanding of the work, will likely cause delays to the publication date of your manuscript. Note: Proofs for Front Matter articles (Editorial, Viewpoint, Symposium, Review, etc...) are generated on a different schedule and may not be made available as quickly.

Soon after your final files are uploaded, the early version of your manuscript will be published online unless you opted out of this process. The date of the early version will be your article's publication date. The final article will be published to the same URL, and all versions of the paper will be accessible to readers.

For Research Articles, you will receive an invoice from PLOS for your publication fee after your manuscript has reached the completed accept phase. If you receive an email requesting payment before acceptance or for any other service, this may be a phishing scheme. Learn how to identify phishing emails and protect your accounts at https://explore.plos.org/phishing.

Thank you again for supporting open-access publishing; we are looking forward to publishing your work in PLOS Neglected Tropical Diseases.

Best regards,

Shaden Kamhawi

co-Editor-in-Chief

PLOS Neglected Tropical Diseases

Paul Brindley

co-Editor-in-Chief

PLOS Neglected Tropical Diseases
